# Supplementary material for: Health risk communication and infodemic management in Iran: development and validation of a conceptual framework
Source: BMJ Open. 2023 Jul 30;13(7):e072326. doi: 10.1136/bmjopen-2023-072326 (PMC10387647; doi:10.1136/bmjopen-2023-072326)
Supplement: Supplementary data [file bmjopen-2023-072326supp001.pdf]

Interview guide

Part I: Demographic information

Age ……….

Gender Female / Male

Educational level ……….

Academic discipline ………

What organizational position were you involved in at the time of the Covid 19 epidemic? ………..

Part II: Perceptions, experiences, future directions

The main purpose of the questions in this section is to identify effective strategies and successful experiences in the field of infodemic management related to Covid 19 in Kerman province. Please answer the following questions based on your experiences or field observations.

1- The spread of misleading, inaccurate, and fake information about COVID-19 disease and vaccination has been one of the consequences of the COVID-19 epidemic, which affects the behavior of society and trust in the health system. What experience did you have in managing misinformation? What did you do in a situation in the province where accurate information was not yet available? Can you explain your own experiences in this field?
2- 2. What challenges and obstacles did you face in combating inaccurate information and infodemic management?
3- What did you do in response to the obstacles and challenges?
4- How did you find out about the effectiveness of your interventions and actions?
5- 5- If the pandemic situation is repeated, what is your approach to managing infodemic?
