# Supplementary material for: Health risk communication and infodemic management in Iran: development and validation of a conceptual framework
Source: BMJ Open. 2023 Jul 30;13(7):e072326. doi: 10.1136/bmjopen-2023-072326 (PMC10387647; doi:10.1136/bmjopen-2023-072326)
Supplement: Supplementary data [file bmjopen-2023-072326supp002.pdf]

Informed Consent

Hi,

My name is Azam Bazrafshan. My colleague and I are from the Kerman University of Medical Sciences. We interview executives, technical experts, decision-makers, and leaders of public health initiatives who had participated in the provincial, or national COVID-19 prevention and control programs to use the results to improve health interventions during epidemics, pandemics, and global health crises. We are intended to investigate processes, infrastructure, strategies, successful experiences and challenges in the field of infodemic management related to Covid 19 in Kerman province. You are being invited to take part in this research because we feel that your experience as a public health leader can contribute much to our understanding and knowledge of processes and infrastructure of infodemic management during health epidemics.

Your participation in this research is entirely voluntary. It is your choice whether to participate or not. If you choose not to participate all the services you receive at this Centre will continue and nothing will change.

In this interview, I will not ask your name, nor will I need your address. All your answers will be completely confidential. We only use the total responses for statistical survey. During this interview, private questions may also be asked and I have to emphasize that although your honest cooperation is valuable, you can answer any question you think appropriate. The estimated time of interview is about 30 minutes and the interview is recorded by tape recorder.

You do not have to take part in this research if you do not wish to do so, and choosing to participate will not affect your job or job-related evaluations in any way. You may stop participating in the interview at any time that you wish without your job being affected. I will give you an opportunity at the end of the interview to review your remarks, and you can ask to modify or remove portions of those, if you do not agree with my notes or if I did not understand you correctly.

I have read the foregoing information, or it has been read to me. I have had the opportunity to ask questions about it and any questions I have been asked have been answered to my satisfaction. I consent voluntarily to be a participant in this study

Signature of Participant ___________________

Date ___________________________Day/month/year

Signature
