# Supplementary material for: Health risk communication and infodemic management in Iran: development and validation of a conceptual framework
Source: BMJ Open. 2023 Jul 30;13(7):e072326. doi: 10.1136/bmjopen-2023-072326 (PMC10387647; doi:10.1136/bmjopen-2023-072326)
Supplement: Supplementary data [file bmjopen-2023-072326supp003.pdf]

Appendix 3. Themes and subthemes related to the challenges and successful activities of infodemic management across provincial/national public health settings in Iran

**Theme**	**Sub-theme**	**Sample Quotes**
**Leadership & governance**		
Ethics	Transparency	When I report falsely on the radio, people would understand. If I have this transparency at the national and university level, people will gain their trust, but this strategy does not exist, it certainly does not exist, neither at the national level, nor at the university or provincial level. (Man, 47 years old)<br><br>There is a level of transparency that the government should have, the officials should have, I would say that it is extremely small considering the structure of our country. (Man, 42 years old)
	Accountability	No one was responsible, a disaster happened, I learned by myself, it was unknown to us, we could have managed this if the ministry had interfered less, the ministry acted badly and this bad behavior spread everywhere.(Man, 55 years old)
	Conflicts of interests	Conflict of interests is one of the most important challenges in the Ministry of Health, which hinders transparency. Profit seeking of companies that produce personal protective equipment, diagnostic and therapeutic equipment, and vaccines, is One of the most important examples of conflicts of interests during the pandemic management. (Man, 70 years old)
Capacity	Infrastructures	We need a quick reaction team that is in contact with academic centers, regularly monitors and examines community's needs and concerns. (Man, 49 years old)<br><br>... and can quickly identify rumours and false information and design an answer to them. (Man, 42 years old)<br><br>A multi-disciplinary risk communication team should be formed to be responsible for informing and making decisions. (Man, 58 years old)
	Rules & regulations	During the pandemic, there were people who published false and contradictory information, and there was no authority to deal with this issue and deal with them judicially, while in many countries, when the issue of people's health is discussed, false information is not allowed to be published. (Man, 70 years old)
	Policies and strategies	There is no specific strategy and program to deal with infodemic and manage risk communication in the country. (Man, 70 years old)
	Role definition	The duties and responsibilities of people in crisis situations should be clear so that everyone does not act and speak as they wish... The goals of the programs should be clear and the responsibilities of each member of the committees should be clearly stated. (Man, 53 years old)
Operational isation	Timeliness	In order to prepare in crisis conditions, it is necessary to make necessary plans before every crisis. (Man, 45 years old)
	Coordination	One of our most important problems was the lack of coordination between the government and the officials in the matter of information. There were several voices and no coordination between different sources in the health department. (Man, 58 years old)

Institutional/provincial design	Knowledge exchange	In the discussion of pandemic management, the provinces were left to their own devices, and no province was aware of the activities of other provinces in the management of the infodemic. (Man, 55 years old)
**Information**		
**Theme**	**Sub-theme**	**Sample quotes**
Surveillance	Timeliness	No real-time data were available about the mortality of COVID-19 cases. Even now, if we refer to the statistical systems, we will not get a correct and reliable information and coordination so that we can inform the community. (Man, 48 years old)<br><br>Unfortunately, many of the events that happened in the country have not been documented and, for example, we do not know how many patients were infected with Corona, how many died. Even the medical and nursing systems used to come and give general information. It showed that either they don't have this information or they don't want to publish it.(Man, 49 years old)
	Data quality	No consensus data were available as mortality data reported by the hospitals, forensic systems, and cemeteries, were very contradictory. (Man, 48 years old)
	Surveillance systems	Surveillance data were extremely out of date, with low quality and consistency. (Man, 54 years old)<br><br>Our surveillance data were not accurate and real-time. (Man, 48 years old)<br><br>Therefore, it seems that we need a system that collects information from the environmental levels in a database in the form of software that has the power of analysis to give us alarms in different places. Its infrastructure in the country is planned as a syndromic care system, but I don't know if it has actually been implemented. (Man, 49 years old)
Knowledge translation	Evidence-informed policy making	No evidence about the effectiveness of interventions were synthesised and published for decision making. (Man, 54 years old)<br><br>The next problem was that the correct information did not reach those who should manage infodemic, for example, the number of patients at any moment, the number of deaths, what was the cause of death. (Man, 52 years old)
	Knowledge translation Capacity	Most of the statistics and information will be based on taste and subjective and this will cause individual perceptions and people will allow themselves to give any statistics. (Man, 48 years old)
Health workforce		
**Theme**	**Sub-theme**	**Sample Quote**
Capacity building	Diversity and flexibility	That's why we have to find an entry in the educational fields and teach this issue seriously in the form of workshops for groups close to graduation or students in the form of refresher courses regularly and continuously. Let's define a retraining unit for it and implement it operationally, not just theoretically. (Man, 48 years old)
Challenges	Limited resources	One of our most important challenges in risk communication and infodemic management is the lack of trained and expert people in this field. (Man, 70 years old)

Engagement	Community involvement	The non-participation of experts from different scientific fields in the pandemic management and informing the people was a big challenge, which caused people who had good experience or knowledge to be ignored, and therefore, unqualified people were in charge of informing the people. (Man, 42 years old)
**Financial resources**		
**Theme**	**Sub-theme**	**Sample Quote**
Government resources	Planning	Particular financial sources should be allocated to the risk communication activities. (Man, 66 years old)
**Media**		
**Theme**	**Sub-theme**	**Sample Quote**
Communication channels	Mass media	National TV and Radio channels are still the biggest and most influential communication channels in Iran. (Man, 46 years old)
	Government & health authorities' websites	We used both video media such as radio and television, as well as written media such as magazines, newspapers, and government websites, which were very active during the Corona era, to communicate the data related to the incidence of the disease in the province and recommend preventive measures. (Man, 49 years old)
	Social media platforms	We have established a social media platform (Instagram) named Dr+ to communicate with people and held online discussion panels with contribution of clinical physicians to address the community's needs and concerns. (Man, 53 years old)
Trust	Source credibility	Communication channels should be used that are highly credible and people trust to them. Some brand communication channels should be developed to maximize the impact and penetration of information among people. (Man, 47 years old)
spokespersons	Competence	For a person to be a spokesperson and to give information, to know how to give information, not to be too hopeful, not to speak too hopelessly, this is real information when we say not only to report numbers... For example, when the pandemic came, someone said that there is nothing, someone said Wow, we are unfortunate, which one of these people should accept when they look at it? (Man, 58 years old)
**Community**		
**Theme**	**Sub-theme**	**Example Strategies**
Diversity	Social context	The penetration rate of scientific issues in our society is low, which is related to various issues, so if we ever want to increase this penetration rate, we have to approach from different social and cultural aspects. (Man, 54 years old)
Engagement & empowerment	interventions	We have developed a community engagement facility to listen the community's needs and expectations and answer to their concerns and questions interactively. (Man, 55 years old)<br><br>Safiran-e-Salamat was a group of trained volunteers who communicate health information with their families and their neighbourhood. (Man, 55 years old)

Trust	Trust to government and health officials	Public opinion has no trust in the government, especially in matters that are officially announced. (Man, 47 years old)<br><br>Anyone, any scientist, any distinguished person, any accepted person comes and says something, the first time people do not accept it, especially if it is actually what the government says or emphasizes, people will definitely look for the opposite and say that there is something fishy about it. (Man, 53 years old)
