# Supplementary material for: Health risk communication and infodemic management in Iran: development and validation of a conceptual framework
Source: BMJ Open. 2023 Jul 30;13(7):e072326. doi: 10.1136/bmjopen-2023-072326 (PMC10387647; doi:10.1136/bmjopen-2023-072326)
Supplement: Author's manuscript [file bmjopen-2023-072326.draft_revisions.pdf]

BMJ Open

BMJ Open is committed to open peer review. As part of this commitment we make the peer review history of every article we publish publicly available.

When an article is published we post the peer reviewers' comments and the authors' responses online. We also post the versions of the paper that were used during peer review. These are the versions that the peer review comments apply to.

The versions of the paper that follow are the versions that were submitted during the peer review process. They are not the versions of record or the final published versions. They should not be cited or distributed as the published version of this manuscript.

BMJ Open is an open access journal and the full, final, typeset and author-corrected version of record of the manuscript is available on our site with no access controls, subscription charges or pay-per-view fees (http://bmjopen.bmj.com).

If you have any questions on BMJ Open's open peer review process please email
info.bmjopen@bmj.com

BMJ Open

Health risk communication and infodemic management in Iran: Development and validation of a conceptual framework

Journal:	*BMJ Open*
Manuscript ID	bmjopen-2023-072326
Article Type:	Original research
Date Submitted by the Author:	01-Feb-2023
Complete List of Authors:	Bazrafshan, Azam; Kerman University of Medical Sciences, HIV/STI Surveillance Research Center, and WHO Collaborative Center for HIV Surveillance<br>Sadeghi, Azadeh; Kerman University of Medical Sciences, HIV/STI Surveillance Research Center, and WHO Collaborative Center for HIV Surveillance; Kerman University of Medical Sciences, Deputy of Health, Department of Communicable Diseases<br>Bazrafshan, Maliheh Sadat; Kerman University of Medical Sciences, HIV/STI Surveillance Research Center, and WHO Collaborative Center for HIV Surveillance<br>Mirzaie, Hossein; Kerman University of Medical Sciences, HIV/STI Surveillance Research Center, and WHO Collaborative Center for HIV Surveillance<br>Shafiee, Mehdi; Kerman University of Medical Sciences, HIV/STI Surveillance Research Center, and WHO Collaborative Center for HIV Surveillance; Kerman University of Medical Sciences, Deputy of Health, Department of Communicable Diseases<br>Geerts, Jaason; Canadian College of Health Leaders; City University of London, Bayes Business School<br>Sharifi, Hamid; Kerman University of Medical Sciences, HIV/STI Surveillance Research Center, and WHO Collaborating Center for HIV Surveillance
Keywords:	COVID-19, Health policy < HEALTH SERVICES ADMINISTRATION & MANAGEMENT, INFECTIOUS DISEASES

SCHOLARONE™
Manuscripts

I, the Submitting Author has the right to grant and does grant on behalf of all authors of the Work (as defined in the below author licence), an exclusive licence and/or a non-exclusive licence for contributions from authors who are: i) UK Crown employees; ii) where BMJ has agreed a CC-BY licence shall apply, and/or iii) in accordance with the terms applicable for US Federal Government officers or employees acting as part of their official duties; on a worldwide, perpetual, irrevocable, royalty-free basis to BMJ Publishing Group Ltd ("BMJ") its licensees and where the relevant Journal is co-owned by BMJ to the co-owners of the Journal, to publish the Work in this journal and any other BMJ products and to exploit all rights, as set out in our licence.

The Submitting Author accepts and understands that any supply made under these terms is made by BMJ to the Submitting Author unless you are acting as an employee on behalf of your employer or a postgraduate student of an affiliated institution which is paying any applicable article publishing charge ("APC") for Open Access articles. Where the Submitting Author wishes to make the Work available on an Open Access basis (and intends to pay the relevant APC), the terms of reuse of such Open Access shall be governed by a Creative Commons licence – details of these licences and which Creative Commons licence will apply to this Work are set out in our licence referred to above.

Other than as permitted in any relevant BMJ Author's Self Archiving Policies, I confirm this Work has not been accepted for publication elsewhere, is not being considered for publication elsewhere and does not duplicate material already published. I confirm all authors consent to publication of this Work and authorise the granting of this licence.

For peer review only

Health risk communication and infodemic management in Iran: Development and validation of a conceptual framework

Authors: Azam Bazrafshan[1], Azadeh Sadeghi[1,2], Maliheh Sadat Bazrafshan[1], Hossein Mirzaie[1], Mehdi Shafiee[1,2], Jaason M. Geerts[3,4], Hamid Sharifi[1*]

1 HIV/STI Surveillance Research Center, and WHO Collaborative Center for HIV Surveillance, Kerman University of Medical Sciences

2 Department of Communicable Diseases, Deputy of Health, Kerman University of Medical Sciences, Kerman, Iran

3 Honorary Visiting Fellow, Bayes Business School, City, University of London, UK.

4 Director of Research and Leadership Development, The Canadian College of Health Leaders, Ottawa, Canada.

*Corresponding author: Hamid Sharifi (Sharifihami@gmail.com)

Abstract

Objective: The COVID-19 pandemic has demonstrated the current gaps in Iran's and other health systems' risk communication. The COVID-19 infodemic has undermined policy responses and amplified distrust and concerns among the Iranian population. This study aims to develop a conceptual framework for health risk communication and infodemic management (RCIM) during epidemics and health emergencies in Iran that could have applications in other contexts.
Design:this study was designed in two phases. Phase 1 involved semi-structured qualitative interviewto explore RCIMstrategies and programs across public health settingsin Iran and develop a conceptual framework. Subsequently, an online expert panel was arranged to evaluate the trustworthiness and credibility of the proposed framework.
Setting: provincial/national public health settings.
Participants:20 key informants from provincial and national public health authorities who contributed to COVID-19 response programs and participated in interviews. Nine experts from diverse academic disciplines, provincial and national settings, and geography who participated in an online expert panel.
Results:the conceptual model adapted from the qualitative interviews and expert panel discussions and was characterized by using the World Health Organization (WHO) health system framework, including leadership and governance, information, health workforce, financial resources, media, and community. Leadership and governance was recognized as the first unique component for developing RCIM in Iran. Developing information infrastructures including high quality surveillance system, training quality health workforce, financial resources, communication channels and community engagement were recognized as other dimensions for developing health risk communication in Iran.
Conclusion: the proposed framework was a step toward establishing a national health risk communication in Iran but more needs to be done to bridge the gap between research, policy and practice. Further investigations are recommended to assess the validity or usefulness of the conceptual framework. This model has the potential to be applied in other contexts or to serve as the foundation for a locally-created equivalent.

Keywords: risk communication, leadership, infodemic,COVID-19, preparedness, Iran

For peer review only - http://bmjopen.bmj.com/site/about/guidelines.xhtml

'Strengths and limitations of this study'

- This study represents field experiences of public health professionals across provincial/national settings in risk communication and infodemic management (RCIM) during the COVID-19 pandemic in Iran.
- Inspecting experiences and perceptions of academics, health professionals and policy-makers ensures validity of the results by approaching the RCIM topic from different perspectives, and establish credibility of the proposed framework by giving an overarching landscape of RCIM across provincial/national public health settings.
- This study validated an overarching conceptual framework with expert panel discussions for establishing a risk communication and infodemic management system during epidemics and health emergencies in Iran.

Introduction

The COVID-19 pandemic has impacted many aspects of population life globally and has led to serious consequences in health, economic, social, cultural, and informational fields (1). Among the social implications of the COVID-19 pandemic,theconstant releases of immediate and extensive healthor other misinformation by non-expert sources, from anonymous social media posts to non-stringently reviewed rapid academic publications, contributed significantly to misguidedhealth policies and a host of deleterious public consequences(2, 3). This phenomenon is called infodemic. According to the World Health Organization (WHO) definition, an infodemic refers tothe distribution of false or misleading information in digital and physical environments during a disease outbreak (4).Infodemic makes populations more vulnerable to disease infection, their side effects, and other related harms (5).

Information overload during the COVID-19 pandemic represents a parallel pandemic whose` transmission rate is much faster than the disease itself, since the rampant prejudicial and erroneous information can trigger wild and accelerated waves of fear to spread in the general population (6).Before the advent of the Internet,one of the main reasons for deaths during epidemics was the lack of sufficient information on the prevention, care, and treatment of the disease(7). But now, social and electronic mediaand instant messaging are the primarysources of misinformation during epidemics and health emergencies, which can have a heightened impact,particularly when people are in lockdown or isolation(8).

The infodemic may severely change the pandemic's course by interfering with population adherence to public health interventions such as wearing masks, social distancing, and vaccination. In particular, the impact of the infodemic on vaccination is critical because it is key to reverting to pre-pandemic normalcy. The risk and experience of infodemic for economically disadvantaged countrieshas been reported to be higher than in developed and richer countries due to a range of inequalities. Lower rates of health literacy,access to reliable health information, and public trust in public health authorities(9) can make people from underdeveloped and developing countries more susceptible to believing fake news and misinformation upon exposure(10, 11). This is compounded by existing inequalities in terms of comparatively limitedhealthcare infrastructure andreduced access to healthcare facilities and public health professionals,make people from these countries more prone to sporadic and ill-advised health behaviors(12). In this context, the spread of health mis-information as part of the infodemic can pose higher distress to populations in underdeveloped and developing countries during epidemics and health emergencies(13), negatively influence public risk perceptions(14-16), and undermine policy and response, increase the spread and burden of the pandemic, and widen global health disparities.

For peer review only - http://bmjopen.bmj.com/site/about/guidelines.xhtml

The infodemic impacts citizens in every country and addressing it is a new and centrally important challenge in managing and responding to the COVID-19 pandemic—and will be so for future epidemics and health emergencies. To understand and counter the rapidly changing nature of the COVID-19 infodemic and develop effective strategies to mitigate its negative effects such as the further spread of misinformation,a number of novel strategies and initiatives have been established across global public health settings. The WHO has been realised asa pioneer in developing guidelines and initiatives to combat misinformation and infodemic management across the world(17). From early in the COVID-19 response, the WHO began to develop internationalstrategies for infodemic management across the countries. These strategieswere developed in cooperation with other organisations, including US Centers for Disease Control and Prevention (CDC) and the Africa Centre for Disease Control and Prevention.To track and address rumours and misinformation surrounding COVID-19 and HIV, UNAIDS and Africa CDC have been running a rumour management system—software that uses machine learning combined with human expertise to collect and analyse rumour data from open-source traditional media (web-based, broadcast), as well as social media (Facebook, Twitter, WhatsApp).The system enables the identification of false and misleading narratives and sentiments related to COVID-19 and HIV(17). In addition, a framework for infodemic management was developed through crowd-sourcing and online consultation with a wide range of global public health professionals(18). Ghana Health Services (GHS) together with the United Nations International Children's Emergency Fund (UNICEF) Country Office have developed a systematic process that effectively identifies, analyses, and responds to COVID-19 and vaccine-related misinformation in Ghana(19).

Risk communication and infodemic management (RCIM) are the core of risk management in epidemics and health emergencies(20). Meanwhile, information monitoring, building e-health literacy and science literacy capacity, encouraging knowledge refinement and quality improvement processes such as fact-checking and accelerating the academic peer-review process to ensure accurate and timely knowledge translation, and minimizing distorting factors such as political or commercial issues are the main pillars of the infodemic management (21).Combating mis- or disinformation online for populations is as critical as ensuring much-needed medical equipment and supplies for health workers are readily available (22). However, in underdeveloped and developing countries, with their existing health information inequalities and public health vulnerabilities, innovative RCIM approaches are needed for combating the infodemic and reducing its effect on population health conditions. In this regard, participatory engagement, and stricter regulations are necessary (23). While some contexts may be more susceptible to the dangerous potential impacts of mis- and dis-information, none is immune and the consequences of failing to combat it strategically and head-on can be dire.

In this study, we aimed to build on and extend previous conceptualisations of capacity building and strengthening relevant to RCIM by describing an overarching conceptual framework of RCIM in Iran by using the World Health Organization (WHO) health system framework. The RCIM field comprises multiple and diverse actors such as researchers, educators, advocates, practitioners, funders and policymakers. We, therefore, hope that this paper will be of interest and relevance to all these groups, with multiple lessons also potentially being transferable to building similar capacities in other underdeveloped and developing countries.

Methods

This sequential mixed-method exploratory study was conducted in two phases. Phase 1involved semi-structured interviews with key informants from provincial and national public health authorities to

For peer review only - http://bmjopen.bmj.com/site/about/guidelines.xhtml

informasetof RCIM components across provincial/ national settings. Phase 2 involvedan onlinepanel of experts from relevant scientific domains to consult and evaluate the conceptual framework's validity, credibility, and transformability. This study followed Standards for reporting qualitative research (SRQR) checklist.

Phase 1: semi-structured interviews

This phase involvedsemi-structured interviews with a purposive sample of 20 Iranian public health professionals across provincial and national health authorities.Study participants included leaders from community health sectors, epidemiology, public health, social medicine, health communication, and sociology disciplines. This phase included a wide range of stakeholders, academics, decision-makers, and leaders from the community and national public health settings whomet the inclusion criteria. Participants were from eight pre-specified provinces:Kerman, Tehran, Fars, Isfahan, Mazandaran, West-Azerbaijan, and Sistanva Baloochestan. Inclusion criteria were 1) having at least one year of experience in either COVID-19 prevention and control programs or decision-making in provincial or national public health settings, and 2) willingness to participate in the study.

An interview guide was developed according to previous studies (Appendix 1). The interview guide focused on the processes, infrastructures,challenges encountered, and best practices relevant to RCIM during the COVID-19 pandemic in Iran. The interview guide was primarily assessed by two expert reviewers. It was subsequently pre-tested with three target population members before the implementation.

The interviews followed a semi-structured design. However, the order of the questions and answers varied according to the participant's responses. The objectives and the activities that were involved in the study were explained to the participants. The principalinvestigator's contact details were provided, and participants' confidentiality was guaranteed. Written consent was sought before the interview, and the participants were asked to email the completed form to the principal investigator (Appendix 2). An experienced interviewer with a background in qualitative research and interviewing expertise conducted the interviews in the Farsi language. Due to COVID-19 social distancing, all interviews were conducted by telephone, audio-recorded, and transcribed verbatim. Interviews ranged between 20–55 minutes (mean = 34 minutes).Interviews were continueduntil the researchers realised they have reached content saturation.

Braun and Clarke's framework for content analysis was appliedto qualitative analysis. MAXQDA 12 (VERBI GmbH, USA) was used for manual coding and content analysis. According to the qualitative interviews and extracted themes, a preliminary list of the RCIM model's components was created. This list comprised 33componentsand was divided into sixpillars (building blocks). The list served as a basis for the conceptual model, which was then discussed with the expert panelin Phase 2.

Phase 2: an expert panel and nominal group technique

In the secondphase, a group of nine participants was purposively selected to verify and prioritise key components of the RCIM model and to evaluate the credibility and transformability of the proposed conceptual model. The panel included a diverse set of stakeholders, academics, decision-makers, and leaders from the community and national public health settings. The inclusion criteria for this phase were 1) having at least three years of professional experience or established research expertise in the fields of public health, epidemiology, crisis management, infodemiology, social media studies, and health communication; and 2) willingness to participate in the study. Potential panel members (n = 9)

For peer review only - http://bmjopen.bmj.com/site/about/guidelines.xhtml

were identified through their academic/consulting/leadership roles in health risk communication or risk management activities across provincial or national health authorities during the COVID-19 pandemic. The expert panel members were primarily approached by email with a short statement of the purpose of the meeting. The conceptual model created following the qualitative interviews was subsequently discussed in the expert panel to evaluate its completenessand trustworthiness in terms of adequately representingessentialcomponentsof anRCIMmodel forthe country. Validating the original model with experts was also intended to augment its quality,reliability, and validity(24, 25).
Following this phase, several modifications were added to the original conceptual model, but no factor was excluded. The requisite consensus level for thisphase was determined as at least 75% agreement.

Patient and Public Involvement statement
No patient or community member was involved in this study.

Results
Phase 1: semi-structured interviews

Participants
Most participants were 51-60 years-old (n=11, 55%) and men (n=19, 95%). Most participants were from medical and public health disciplines (n=17, 85%) with a history of working as a provincial or national health officer (n=14, 70%). Participants were mostly from Tehran (n=7, 35%) and Kerman provinces (n=5, 25%)(Table 1).

Table 1 Demographic characteristic of the participants in the interviews (Phase 1) to inform the development of a conceptual framework for health risk communication and infodemic management in Iran

Demographic characteristics	Frequency (%)
Residence at the time of interviews	
Tehran	7(35)
Kerman	5 (25)
Fars	2 (10)
Isfahan	2 (10)
Mazandaran	1 (5)
West Azerbaijan	1 (5)
SistanvaBaluchestan	1 (5)
Kermanshah	1 (5)
Age	
40-49	6 (30)
50-59	11 (55)
60>	3 (15)
Gender	
Men	19 (95)
Women	1 (5)
Academic Discipline	
General medicine	4 (20)

For peer review only - http://bmjopen.bmj.com/site/about/guidelines.xhtml

Epidemiology	6 (30)
Social medicine	2 (10)
Health education & promotion	1 (5)
Health policy	2 (10)
Sociology	3 (15)
Infectious disease	2 (10)

The analysis of the qualitative data collected during the key informant interviews revealed 948 open codes and 84 sub-themes. Sub-themes were subsequently classified into 33 components(Appendix 3).The next step involved organising thesecomponents according tothe six Iranian health system's pillars: leadership and governance, information, health workforce, financial resources, media, and community, which formed the initial RCIM conceptual model.

Theme 1: Leadership and Governance

The pandemic has highlighted that effective leadership andtransparent decision-making and communication are essential to any successful public health strategy (25). Commensurately, all respondents mentioned leadership and governance as essential foundations. Respondents frequently emphasised transparency in decision-making and communication and accountability as important characteristics of effective leadership and governance. Lack of transparency and accountability among health officials and government authorities were among the country's substantial weaknesses in risk communication. More thanhalf of the respondents suggestedthat financial and competing interests of public health officials intentionally caused the lack of transparency in information communication during the COVID-19 pandemic. The lack of transparency posed major negative consequences to public trust.

Almost all respondents emphasised that the health system needs a robust risk communication strategyand increasedinfodemic management capacity bydeveloping infrastructures for monitoring, infodemiology (information epidemiology), infoveillance(26), social listening, communicating with the public, and guidance distillation based on the best available science. The purpose of building this capacity is tobe able to detect outbreaks of potentially harmfulmisinformation, rumours, and falsehoods and to counter them with facts or other reliable information in a targeted way for each audience. One respondent emphasized thatbuilding capacity should involve designing an infodemic management system that defines national and provincial responsibilities that is based on lessons learnt from credible global guidelines, local failures, challenges, successes, and leading practices – locally and elsewhere.A multi-disciplinary and team-based approach should establish an independent rapid-response core team with clear roles and accountabilities to screen and identify community needs, concerns, and misinformation sources, rapidly respond to the potential risks, and prevent or mitigate the viral spread of misinformation across the communities.

Developing, implementing, and evaluating communication policies and strategies for potential risks were frequently mentioned by respondents. According to respondents, the lack of national and provincial policies and programs for RCIMcontributed tomajor negative consequences to the national COVID-19 control and management efforts. Poormanagement of the COVID-19 infodemic, poor communication with the pubic and other stakeholders, and a lack of national andprovincial strategies to address misinformation were major shortcomings of risk communication and infodemic management in Iran. Two respondents stated that providing people with tools forfiltering, assessing and fact-checking information isessential to combat misinformation during the pandemic and health emergencies. Five

For peer review only - http://bmjopen.bmj.com/site/about/guidelines.xhtml

respondents believed that using a well-known and reliable communication channel and technology-based interventions would maximise the spread of valid information and impact of communication efforts and strategies.

The general publicand multi-sector involvement were emphasised for RCIM during a pandemic. Seven respondents believed that pharmaceutical companies were a major source of spreading misinformation during the pandemic. Participants believed that the financial and competing interests of public health officialsdeterred them from spreading of valid information about the efficacy of some new and underdeveloped medications and vaccines. As a number of health officials were among shareholders of pharmaceutical industries, theyadvertised some drugs or public health products and subsequently caused a fake and unrealistic demand among the population. Over half of the respondents indicatedthat top-down interventions with a lack of community-based approacheswere among major barriers to acceptance ofCOVID-19 prevention and control interventions during the pandemic. Therefore, multi-sector and community involvement could reduce the potential risk of existing conflicts of interest and improve motivation to participate in information communication and management of the crisis actively. In this context, community-based approaches, such as Safiran-e-Salamat (a community-based intervention established in Tehran), and social influencers were reported as facilitators for effective risk communication and infodemic management across provincial settings.

Lack of crowd-sourcing and ineffective use of institutional and provincial potential capacities and infrastructures during the crisis werecitedby four participants as major barriers to proper COVID-19 infodemic management during the pandemic. These respondents elaboratedthat medical universities within the provinces were isolated from the national health authorities and not supported by the Ministry of Health in planning and decision-making. Therefore, it was recommended by the respondents to establish a network capacity to share experiences, challenges, andbest practices, of information communication during the potential risks.

Theme 2: Information

Developing a network platform to systematically collect, analyse, and interpretepidemiologic data from the community and quickly disseminate the resulting information was considered an important characteristic of risk communication by the respondents. Almost all respondents emphasised that lack of access to real-time, valid, and high-quality data about the incidence, mortality, and burden of the COVID-19 disease in different provinces intensified the potential risk of misinformation among the population.

Lack of access to high-quality surveillance data for research activities and to inform responses to potential and emergent challenges reduced transparency. It raised dramatic social concerns about the government's ability to estimatethe spread of the disease or evaluatethe effect of specific policies on population health. The respondents frequently reported the lack of evidence-based policies and practices as a major barrier to effective risk communication and infodemic management. In addition, the lack of substantial resources to handle the multiplication of data sources and information producers, to monitor disease trends regularly, and to appraise the quality of data sources were reported as major barriers to the effective use of surveillance data for decision-making during the pandemic.

According to one respondent, misinterpretation of facts and available low-value data by health officials was considered as a source of misinformation during the pandemic. Some politicians, health officials, and media interpreted data according to their financial, commercial, and political interests, as well as selective reporting and misunderstanding. Therefore, developing and facilitating accurate knowledge

For peer review only - http://bmjopen.bmj.com/site/about/guidelines.xhtml

translation is required to prevent misinformation across different sectors of society. People's political, commercial, and financial interests can lead them to distort scientific messages; therefore, respondents believed that knowledge translation efforts are necessary to minimise these factors or at least clearly disclose and call out.

Theme 3: Health workforce

All respondents highlighted the need for a well-trained workforce with a mix of skills that can contribute to risk communication and infodemic management activities. According to respondents, the lack of qualified and well-trained spokespersons greatly diminishedthe quality of risk communication activities during the pandemic. Respondents referenceda range of competenciesnecessary to improve the quality of the health workforce education and practice, which can also be used to design new educational courses and curriculums.

All respondents agreed that the Iranian scientists and experts have not helped substantially to mitigate the effects of the infodemic and prevent misinformation. In some cases, scientists and academic experts in infodemic management were considered sources of misinformation, seen ascontributingto the infodemic trends bypublishing low-quality scientific papers and providing sensational or exaggerated information about new treatments. In addition, over half of the respondents highlighted the need to involve professional councils, NGOs, health volunteers, and interested experts as additional workforce sources to improve the speed and effectiveness of the response to misinformation and infodemic. Therefore, involving public health agencies, epidemiologists, data scientists, and sociologists who have unique expertise and credibility to guide risk communication and infodemic management is recommended.

Theme 4: Financing

Three respondents highlighted effective financial resource allocation to risk communication and infodemic management to support communication strategies during the crisis. One respondent argued that multi-sector collaborations could reduce the risk of underfunding communication responses. Using technology-based interventions, such as text-messaging approaches, could improve the cost-effectiveness of communication strategies.

Theme 5: Media

All respondents mentioned characteristics related to media. About half of the respondents argued that TV and mass media contributed to the COVID-19 infodemic. Broadcasting misleading and low-value information, inviting unprofessional experts, and raising debates about the performance of public health agencies reduced public trust and led the community to informal and social media channels. However, with the broad coverage and penetration of radio and TV as dominant communication channels in most parts of the country, involvement, and collaboration with mass media, including radio and TV, could improve the effectiveness of risk communication strategies.

The need to improve government and health authorities' websites to disseminate real-time and high-quality information was mentioned by three respondents. Governmental websites are considered the source of credible information for most people. Still, the weak contribution of these media in risk communication and infodemic management was considered as a barrier to preventing misinformation.

Respondents frequently mentioned social media platforms as important communication channels for most communities. According to two respondents, dissemination of valid and high-quality data through social media channels influenced the impact of local interventions and improved vaccination coverage for vulnerable and ethnic populations, particularly in Sistan-va-Baluchestan and West Azerbaijan. Due to

For peer review only

For peer review only - http://bmjopen.bmj.com/site/about/guidelines.xhtml

higher accessibility, social media platforms were highly utilised by younger adults and geographically distanced locations; therefore, using these platforms to address distanced populations can improve the speed and effectiveness of interventions. In addition, reduced public trust in formal and government communication channels highlighted the perceived credibility and trustworthiness of social media platforms. This can affect the community's acceptance of public health interventions.

Theme 6: Community

The community is frequently reported as an important characteristic of risk communication and infodemic management. Listening to the community's concerns, identifying their information needs, communicating with communities, and adjusting based on their diversity, culture, and ethnicity are the cornerstone components of risk communication during the crisis. However, available experiences suggested that the lack of community-based approaches reduced the effectiveness of risk communication efforts during the COVID-19 pandemic in most provincial settings. Training critical thinking, media, and health literacy should be considered and highly prioritised to improve engagement and active contribution of people in risk communication and infodemic management. Competent and well-empowered communities could minimise misinformation and infodemic consequences and enable communities to develop their own solutions. In this context, building and maintaining public trust in public health agencies, health professionals, and government authorities couldmaximise social cohesion and successfully respond to potential risks during the crisis.

Phase 2: Expert panel using the NGT approach

In this phase, the proposed conceptual model of RCIM in Iran was discussed by the online expert panel, and the trustworthiness of the model was finally approved by the entire panel members (100% agreement) (Figure1).

Figure 1: A conceptual model of components and infrastructures of health risk communication and infodemic management system in Iran

The panellistsrecommended taking a comprehensive communication risk approach, which considersthe characteristics of potential audiences and stakeholders, including the community, scientists and experts, and pharmaceutical industries through physical and virtual communication channels (Figure 2).

Figure2: Elements of the COVID-19 risk communication and infodemic management in Iran, source: own production

Discussion

The present research examined RCIM processes and infrastructure in Iran during the COVID-19 pandemic and outlined key components of an effective RCIM model as an essential component of health emergency readiness and response activities. Further, a multi-disciplinary expert panel confirmed the credibility and trustworthiness of the proposed model. Following are some insights and

For peer review only - http://bmjopen.bmj.com/site/about/guidelines.xhtml

attention points on effective RCIMthat emerged from the qualitative interviews and expert panel discussions. Our findings suggest that establishing an integrated national RCIM program, strengthening existing fundamental capacities of RCIM, and systematic listening to social concerns are essential for health emergency readiness and response activities. Epidemics and health emergencies are usually accompanied by a wave of disinformation that undermines policy responses and amplifies community distrust and concerns. Understanding the source of disinformation and responding rapidly to evolving risks can mitigate potential negative ramifications. Further, only if public concerns are entirely understood can they be adequately addressed and health information about the evolving risk communicated effectively in this context. Systematic listening refers to receiving information from and about public concerns and information needs through multiple channels, including the media, social media, and key intermediaries, and then analysing this information and responding appropriately to public needs. According to our findings, essential national andprovincial capacities that explore, track, and monitor the community and high-risk groups' information needs, assess the degree to which available information matches their needs, and provide policymakers and health practitioners with the knowledge needed to implement appropriate and effective strategies that target identified information inequalities and social concerns are a required component for effective health risk communication governance.

Financial resources and technical expertise are fundamental to building such a health RCIM system.In particular, developing health risk communication and infodemic management system capacity is poorly funded in the country andtechnical health risk communication experts in the country are critically limited. There are media and public communication experts. Community-level health promotion experts often work in areas such as communicable diseases, child and maternal health, and other communicable or non-communicable diseases. However, effective RCIM requires personnel with training andexpertise who can be available in the time-limited and high-stress conditions of an epidemic or health emergency. Some capacity-building workshops were held during the COVID-19 pandemic bythe ministry of health and medical universities. Still, these have largely been ad hoc, of short duration (less than a week), and of variable quality. Those trained have often been public health professionals who then move on to other areas of public health. A planned and institutionalised approach to capacity-building is required to have an adequate pool of trained experts for epidemics and health emergencies.Therefore, financial resources and building risk communication expertise are critical priorities for the country. Obtaining both these resources will require the endorsement of senior policy-makers. Advocacy to policy-makers and key decision-makers on the role and impact of RCIM is very important.

RCIM is a broad and multi-disciplinary field involving health communication, health education, public affairs, behavior change communication, and social mobilisation. It is therefore required to build the capacity of key contributors to verify, filter, and curate health information and use diverse communication channels to target public audiences(27). Community-basedorganisations, patient advocacy groups, professional associations, and non-governmental organisations with reputable brands, organisational resources, and a network of relationships can be leveraged to improve health risk communications. Existing evidence demonstrates that by partnering with local public health experts and policymakers to create information hubs and community outreach programs(28), these groups can significantly improve their ability to serve the information needs and concerns of diverse communities while also advocating for policy solutions. Existing evidence demonstrates that involving community members as planners, and attendees in pre-crisis planning activities, leads to increased preparedness

For peer review only - http://bmjopen.bmj.com/site/about/guidelines.xhtml

and response activities. Therefore training in roles and responsibilities, relationship building, and team-buildingare required strategies to facilitate and strengthen the contribution of community-basedorganisations, expert associations, and other relevant partners during epidemics and health emergencies(29).

Our findings also revealed that lack of direct financial resources allocation to health risk communication and infodemic management hindered support of risk communication activities during the pandemic in Iran. However, resource mobilisation and the use of non-governmental resources were reported as strategies to address this critical challenge within the country's national and provincial settings. According to available evidence, shortcomings in financial resources are a common challenge in health risk communication management in most countries. Evidence from south-east Asia(30) revealed that during the COVID-19 pandemic, few countries allocated resources to emergency risk communication. However, some specific areas have budgets, such as information education communication materials.

Developing or sustaining reputed and well-trusted communication channels are critically required to maximise the effectiveness and impact of communication strategies. How the community perceives various epidemics and health emergencies, what they perceive to be their role, how they are influenced, and how their views tally with the biomedical approach, are not entirely investigated in the country. According to our findings, a lack of public trust in mass media and government channels directed Iranian citizens to the wide use of online social networks. Due to the dramatic reduction in social capita, most Iranians distrust governmental information sources, and this fact challenged the community's compliance with preventive behaviors (COVID-19 vaccination) during the COVID-19 pandemic. Lack of trust in the government as a source of information was reported globally inexisting literature. According to recent evidence, only 40% of the European citizens from the Economic Co-operation and Development countries participated in a survey and trusted their governments as sources of information about the Corona Virus(31). False claims about the activities, statistics, or policies of public and government authorities were reported as a major source of disinformation during the COVID-19 pandemic, suggesting that "governments have not always succeeded in providing clear, useful, and trusted information to address pressing public questions" (32). Meanwhile, disinformation and claims may also be falsely attributed to official and governmental sources, amplifying this problem. In this regard, delivering truthful, evidence-informed, and compelling information to various audiences through their preferred channels and understanding behavioral and psychological biases is recommended. This is especially important for young audiences, who tend to access news and information predominantly via social media platforms(33). It is, therefore, a critical issue for health risk communication and infodemic management to ensure key factual messages reach all audiences. It is also important to effectively leverage the channel through which various audiences are relayed since different groups are likelier to trust media outlets that align with their views.

As substantial social, contextual, economic, and geographical diversity exists within the country, health risk communication response to epidemics and health emergencies will also require diverse community-based approaches. Ethnographic and anthropological/social research on epidemics and health emergencies in the country will help to improve understanding of the acceptability of response to emergencies and public health interventions. According to our interviews and expert panel discussion, the community was considered as a missing piece of RCIM strategies in Iran. Information needs and concerns (e.g., disabilities, gender, age, literacy, cultural/ethnic backgrounds, access to technology) of the general Iranian population remained unexplored. In addition, the participatory engagement of citizens in a collective response to the COVID-19 infodemic was not just insufficient at times but also

For peer review only - http://bmjopen.bmj.com/site/about/guidelines.xhtml

discouraged in several instances. During the COVID-19 pandemic, national health authorities and governments in most countries predominantly demonstrated top-down communication strategies (34). Effective RCIM requires a whole-of-society effort to sustain a healthy information ecosystem. Understanding the needs and concerns of vulnerable groups who might experience barriers to accessing accurate health information, care, and support,or be at higher risk of exposure and secondary impacts, such as children and adults with disabilities, is critically important(34). Effective risk communication can save lives during epidemics and health emergencies; however, existing evidence revealed that inadequate risk communication resulted in high exposure and loss of lives, as seen in Iran and Italy in the first wave(34, 35). Training and advising the general population on how to consume and share health information responsibly may be an effective strategy to improve the engagement and participation of public communities in risk communication and infodemic management. Investing in the community's media literacy, health literacy, and critical thinking skills before the crisis can prepare society to mitigate the physical and emotional consequences of false news and disinformation and increase resilience(36). As disinformation and infodemic during epidemics and health emergencies undermine trust, amplify fears and consequently affect countries' responses to the global pandemic, tailored strategies to build and maintain trust among the public community are of utmost importance. Therefore, to be effective and foster public trust in government, any activities conducted in health risk communication and infodemic management must be guided by the principles of transparency, integrity, accountability, and community participation.

We address some limitations of the study. First, as qualitative research is open-ended, respondents have more control over the content of the data collected, and the investigator fails to verify the results objectively. Additionally, investigating causality and replicating the study is rather difficult in qualitative studies. Second, the nature of purposive sampling may generate a sampling bias in our results. Particularly, included provinces may only be a representative sample of some of the public health systemsin Iran. Second, our findings are based on data collected during the last wave of the pandemic. As the RCIM strategies have evolved and improved during different waves of the pandemic, and lessons from the first waves of COVID-19 and other countries' experiences helped all stakeholders to be better prepared for further waves of the pandemic; therefore, challenges and gaps of RCIM activities in previous waves may not extensively be revealed by the study participants. However, challenges and gaps experienced during the first waves of the pandemic may not always be consistent in the future. Therefore, our findings from the last waves of the pandemic provideinformation for building an overall perspective to understand the strengths and limitations of RCIM activities in Iran.

Conclusion

This study focused on how ineffective RCIM impeded the emergency response in Iran's COVID-19 management and discussed principal infrastructures and processes for effective risk communication. Following a qualitative approach, it was found that Iran's government and national public health authorities did not infuse a scientific and strategic RCIM into decision-making.Consequently, access to high-quality and real-time information was extensively restricted, and not publicly available, and the provincial public health settings failed to establish effective community engagements including experts, researchers, professional councils, and NGOs to facilitate knowledge translation and utilisation. Further, the extensiveuse of social media platforms and mass media worsened the circulation of rumours, fake news, and disinformation and led to public distrust. The lessons learned from the outbreak management and response in Iran suggest that RCIM should be an essential component of health

For peer review only - http://bmjopen.bmj.com/site/about/guidelines.xhtml

emergency readiness and response activities. A national RCIM program should be established to support the required infrastructures, personnel, and processes to address communication challenges during epidemics and health emergencies. This should be based on a conceptual model of RCIM to illustrate a collaborative and interdependent context of risk communication activities, implying that any improvements in these areas require an integrated and holistic approach. The government, pharmaceutical industries, experts, and the public should be involved in time, contributing diverse views and fulfilling respective responsibilities. The conceptual model presented here has the potential to be either be implemented or serve as the foundation for the creation of a similar model in other contexts. Sharing experiences, challenges, and leading practices among jurisdictions can further improve the reliability and credibility of guidance and strategies.

Ethics approval and consent to participate

This study was approved by the Research Ethics Committee of Kerman University of Medical Sciences (**IR.KMU.REC.1400.379).** The Declaration of Helsinki was followed and informed consent was obtained from participants before starting the data collection stage.

Availability of data and materials

The datasets used and/or analysed during the current study are available from the corresponding author on reasonable request.

Competing interests

All authors declared that they had no competing interests.

Funding

This study was financially supported by World Health Organization-Regional Office for the Eastern Mediterranean (WHO-EMR) Call for Proposals for Special Grant for COVID-19 Research, 2022 (**WHO Reference: 2022/1291032-0**)

Authors' contributions

AB contributed to the project concept and manuscript design, qualitative data collection and interpretation, critical review of the manuscript writing and discussion of the manuscript. AS worked on data analysis, data interpretation, writing of the manuscript.MSB worked on data analysis, data interpretation, writing of the manuscript. HM worked on literature search, data interpretation, writing of the manuscript. MS worked on data analysis, data interpretation, writing of the manuscript. JMG worked on data analysis, data interpretation, writing of the manuscript. HS worked on the project concept and manuscript design, supervising, critical review of the manuscript writing and discussion of the manuscript. All authors read and approved the final manuscript

Patient or public involvement

No patient or public was involved in the present study.

For peer review only - http://bmjopen.bmj.com/site/about/guidelines.xhtml

Acknowledgements

The authors wish to thank provincial and national public health professionals who contributed to this study for all their scientific support to the research project and team members.

References

1. Muñoz-Sastre D, Rodrigo-Martín L, Rodrigo-Martín I. The Role of Twitter in the WHO's Fight against the Infodemic. International Journal of Environmental Research and Public Health. 2021;18(22):11990.
2. Mheidly N, Fares J. Leveraging media and health communication strategies to overcome the COVID-19 infodemic. Journal of Public Health Policy. 2020;41(4):410-20.
3. The Lancet Infectious D. The COVID-19 infodemic. The Lancet Infectious diseases. 2020;20(8):875-.
4. Health Topics: Infodemic [Internet]. Available from: https://www.who.int/health-topics/infodemic#tab=tab_1.
5. Yang K-C, Pierri F, Hui P-M, Axelrod D, Torres-Lugo C, Bryden J, et al. The covid-19 infodemic: Twitter versus facebook. Big Data & Society. 2021;8(1):20539517211013861.
6. Patel MP, Kute VB, Agarwal SK, Nephrology C-WGoISo. "Infodemic" COVID 19: More Pandemic than the Virus. Indian journal of nephrology. 2020;30(3):188-91.
7. Rathore FA, Farooq FJJPMA. Information overload and infodemic in the COVID-19 pandemic. 2020;70(5):S162-S5.
8. Fernández-Torres MJ, Almansa-Martínez A, Chamizo-Sánchez R. Infodemic and fake news in Spain during the COVID-19 pandemic. International journal of environmental research and public health. 2021;18(4):1781.
9. Vinck P, Pham PN, Bindu KK, Bedford J, Nilles EJ. Institutional trust and misinformation in the response to the 2018–19 Ebola outbreak in North Kivu, DR Congo: a population-based survey. The Lancet Infectious Diseases. 2019;19(5):529-36.
10. Sørensen K, Pelikan JM, Röthlin F, Ganahl K, Slonska Z, Doyle G, et al. Health literacy in Europe: comparative results of the European health literacy survey (HLS-EU). European journal of public health. 2015;25(6):1053-8.
11. Lorini C, Ierardi F, Bachini L, Donzellini M, Gemmi F, Bonaccorsi G. The antecedents and consequences of health literacy in an ecological perspective: results from an experimental analysis. International journal of environmental research and public health. 2018;15(4):798.
12. Carr-Hill R, Currie E. What explains the distribution of doctors and nurses in different countries, and does it matter for health outcomes? Journal of Advanced Nursing. 2013;69(11):2525-37.
13. Zar HJ, Dawa J, Fischer GB, Castro-Rodriguez JA. Challenges of COVID-19 in children in low-and middle-income countries. Paediatric respiratory reviews. 2020;35:70-4.
14. Lohiniva AL, Pensola A, Hyökki S, Sivelä J, Tammi T. COVID-19 risk perception framework of the public: an infodemic tool for future pandemics and epidemics. BMC Public Health. 2022;22(1):1-9.
15. Islam MS, Sarkar T, Khan SH, Kamal A-HM, Hasan SM, Kabir A, et al. COVID-19–related infodemic and its impact on public health: A global social media analysis. The American journal of tropical medicine and hygiene. 2020;103(4):1621.
16. Karabela ŞN, Coşkun F, Hoşgör H. Investigation of the relationships between perceived causes of COVID-19, attitudes towards vaccine and level of trust in information sources from the perspective of Infodemic: the case of Turkey. BMC Public Health. 2021;21(1):1-2.
17. Organization WH. Infodemic management: an overview of infodemic management during COVID-19, January 2020–May 2021. 2021.

For peer review only - http://bmjopen.bmj.com/site/about/guidelines.xhtml

18. Tangcharoensathien V, Calleja N, Nguyen T, Purnat T, D'Agostino M, Garcia-Saiso S, et al. Framework for managing the COVID-19 infodemic: methods and results of an online, crowdsourced WHO technical consultation. Journal of medical Internet research. 2020;22(6):e19659.
19. Lohiniva A-L, Nurzhynska A, Hudi A-h, Anim B. Infodemic Management Using Digital Information and Knowledge Cocreation to Address COVID-19 Vaccine Hesitancy: Case Study From Ghana. JMIR infodemiology. 2022;2(2):e37134.
20. Naeem SB, Bhatti R. The Covid-19 'infodemic': a new front for information professionals. Health Information & Libraries Journal. 2020;37(3):233-9.
21. Eysenbach G. How to fight an infodemic: the four pillars of infodemic management. Journal of medical Internet research. 2020;22(6):e21820.
22. Radu R. <? covid19?> Fighting the 'Infodemic': Legal Responses to COVID-19 Disinformation. Social Media+ Society. 2020;6(3):2056305120948190.
23. Hua J, Shaw R. Corona virus (Covid-19)"infodemic" and emerging issues through a data lens: The case of china. International journal of environmental research and public health. 2020;17(7):2309.
24. Olson CM. Consensus statements: applying structure. JAMA. 1995;273(1):72-3.
25. Geerts JM, Kinnair D, Taheri P, Abraham A, Ahn J, Atun R, et al. Guidance for health care leaders during the recovery stage of the COVID-19 pandemic: a consensus statement. JAMA network open. 2021;4(7):e2120295-e.
26. Eysenbach G. Infodemiology and infoveillance: framework for an emerging set of public health informatics methods to analyze search, communication and publication behavior on the Internet. Journal of medical Internet research. 2009;11(1):e1157.
27. Goulbourne T, Yanovitzky I. The communication infrastructure as a social determinant of health: implications for health Policymaking and practice. The Milbank Quarterly. 2021;99(1):24.
28. Hering JG. Do we need "more research" or better implementation through knowledge brokering? Sustainability Science. 2016;11(2):363-9.
29. World Health Organization. Communicating risk in public health emergencies: a WHO guideline for emergency risk communication (ERC) policy and practice: World Health Organization; 2017.
30. Ofrin R, Buddha N, Htike M, Bhola A, Bezbaruah S. Strengthening risk communication systems for public health emergencies in the WHO South-East Asia Region. WHO South-East Asia Journal of Public Health. 2020;9(1):15-20.
31. Eldman. Eldman Trust Baromter 2020 2020 [Available from: https://www.edelman.com/sites/g/files/aatuss191/files/2020-03/2020%20Edelman%20Trust%20Barometer%20Coronavirus%20Special%20Report_0.pdf.
32. Brennen JS, Simon FM, Howard PN, Nielsen RK. Types, sources, and claims of COVID-19 misinformation: University of Oxford; 2020.
33. Neavel C, Watkins SC, Chavez M. Youth, Social Media, and Telehealth: How COVID-19 Changed Our Interactions. Pediatric Annals. 2022;51(4):e161-e6.
34. Khan S, Mishra J, Ahmed N, Onyige CD, Lin KE, Siew R, et al. Risk communication and community engagement during COVID-19. International Journal of Disaster Risk Reduction. 2022;74:102903.
35. Chakrabarti A. Confusion in Italy, clarity in Singapore — how govts are communicating during coronavirus 2020 [cited 2022 26/11]. Available from: https://theprint.in/world/confusion-in-italy-clarity-in-singapore-how-govts-are-communicating-during-coronavirus/378932/.
36. Xie L, Pinto J, Zhong B. Building community resilience on social media to help recover from the COVID-19 pandemic. Computers in Human Behavior. 2022;134:107294.

For peer review only

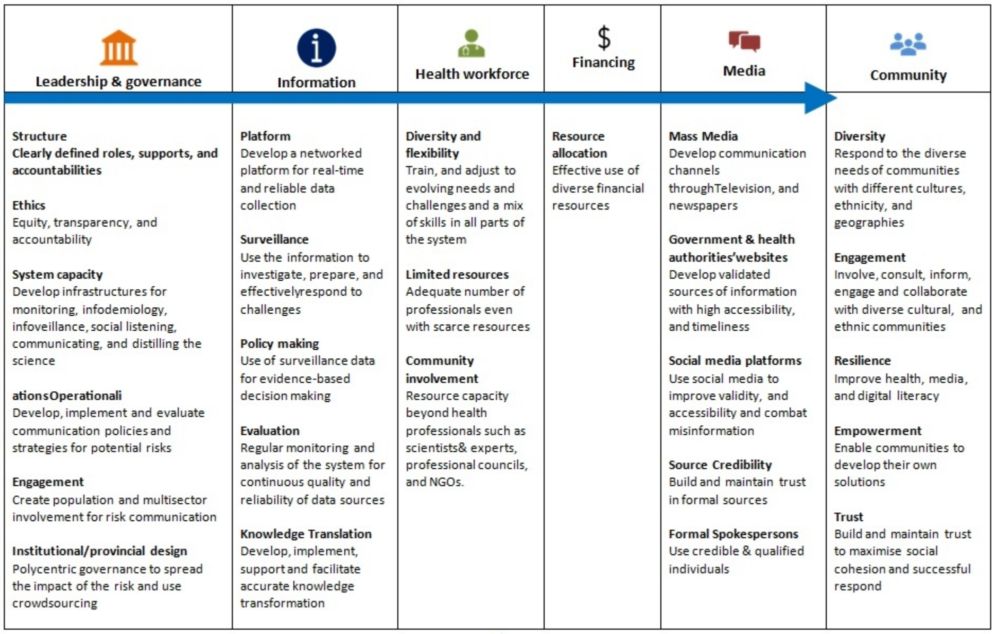

Figure 1

248x158mm (96 x 96 DPI)

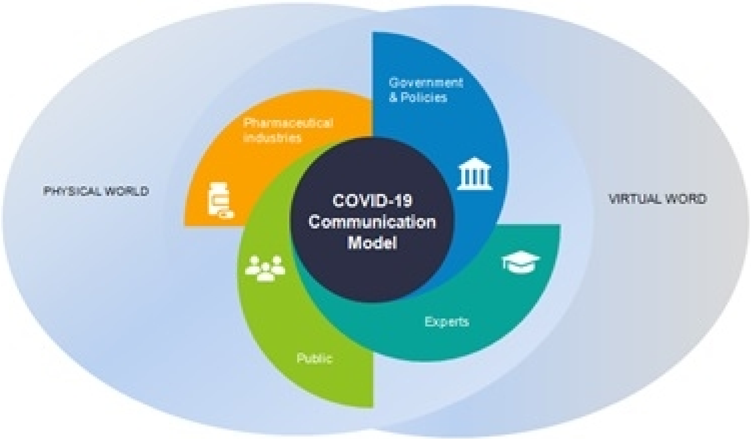

Figure 2

106x77mm (96 x 96 DPI)

Interview guide

Part I: Demographic information

Age ……….

Gender Female / Male

Educational level ……….

Academic discipline ………

What organizational position were you involved in at the time of the Covid 19 epidemic? ………..

Part II: Perceptions, experiences, future directions

The main purpose of the questions in this section is to identify effective strategies and successful experiences in the field of infodemic management related to Covid 19 in Kerman province. Please answer the following questions based on your experiences or field observations.

1- The spread of misleading, inaccurate, and fake information about COVID-19 disease and vaccination has been one of the consequences of the COVID-19 epidemic, which affects the behavior of society and trust in the health system. What experience did you have in managing misinformation? What did you do in a situation in the province where accurate information was not yet available? Can you explain your own experiences in this field?
2- 2. What challenges and obstacles did you face in combating inaccurate information and infodemic management?
3- What did you do in response to the obstacles and challenges?
4- How did you find out about the effectiveness of your interventions and actions?
5- 5- If the pandemic situation is repeated, what is your approach to managing infodemic?

For peer review only

Informed Consent

Hi,

My name is Azam Bazrafshan. My colleague and I are from the Kerman University of Medical Sciences. We interview executives, technical experts, decision-makers, and leaders of public health initiatives who had participated in the provincial, or national COVID-19 prevention and control programs to use the results to improve health interventions during epidemics, pandemics, and global health crises. We are intended to investigate processes, infrastructure, strategies, successful experiences and challenges in the field of infodemic management related to Covid 19 in Kerman province. You are being invited to take part in this research because we feel that your experience as a public health leader can contribute much to our understanding and knowledge of processes and infrastructure of infodemic management during health epidemics.

Your participation in this research is entirely voluntary. It is your choice whether to participate or not. If you choose not to participate all the services you receive at this Centre will continue and nothing will change.

In this interview, I will not ask your name, nor will I need your address. All your answers will be completely confidential. We only use the total responses for statistical survey. During this interview, private questions may also be asked and I have to emphasize that although your honest cooperation is valuable, you can answer any question you think appropriate. The estimated time of interview is about 30 minutes and the interview is recorded by tape recorder.

You do not have to take part in this research if you do not wish to do so, and choosing to participate will not affect your job or job-related evaluations in any way. You may stop participating in the interview at any time that you wish without your job being affected. I will give you an opportunity at the end of the interview to review your remarks, and you can ask to modify or remove portions of those, if you do not agree with my notes or if I did not understand you correctly.

I have read the foregoing information, or it has been read to me. I have had the opportunity to ask questions about it and any questions I have been asked have been answered to my satisfaction. I consent voluntarily to be a participant in this study

Signature of Participant ___________________

Date ___________________________Day/month/year

Signature

For peer review only

Appendix 3. Themes and subthemes related to the challenges and successful activities of infodemic management across provincial/national public health settings in Iran

Theme	Sub-theme	Sample Quotes
**Leadership & governance**		
Ethics	Transparency	When I report falsely on the radio, people would understand. If I have this transparency at the national and university level, people will gain their trust, but this strategy does not exist, it certainly does not exist, neither at the national level, nor at the university or provincial level. (Man, 47 years old)
		There is a level of transparency that the government should have, the officials should have, I would say that it is extremely small considering the structure of our country. (Man, 42 years old)
	Accountability	No one was responsible, a disaster happened, I learned by myself, it was unknown to us, we could have managed this if the ministry had interfered less, the ministry acted badly and this bad behavior spread everywhere.(Man, 55 years old)
	Conflicts of interests	Conflict of interests is one of the most important challenges in the Ministry of Health, which hinders transparency. Profit seeking of companies that produce personal protective equipment, diagnostic and therapeutic equipment, and vaccines, is One of the most important examples of conflicts of interests during the pandemic management. (Man, 70 years old)
Capacity	Infrastructures	We need a quick reaction team that is in contact with academic centers, regularly monitors and examines community's needs and concerns. (Man, 49 years old)<br><br>... and can quickly identify rumours and false information and design an answer to them. (Man, 42 years old)<br><br>A multi-disciplinary risk communication team should be formed to be responsible for informing and making decisions. (Man, 58 years old)
	Rules & regulations	During the pandemic, there were people who published false and contradictory information, and there was no authority to deal with this issue and deal with them judicially, while in many countries, when the issue of people's health is discussed, false information is not allowed to be published. (Man, 70 years old)
	Policies and strategies	There is no specific strategy and program to deal with infodemic and manage risk communication in the country. (Man, 70 years old)
	Role definition	The duties and responsibilities of people in crisis situations should be clear so that everyone does not act and speak as they wish... The goals of the programs should be clear and the responsibilities of each member of the committees should be clearly stated. (Man, 53 years old)
Operationalisation	Timeliness	In order to prepare in crisis conditions, it is necessary to make necessary plans before every crisis. (Man, 45 years old)
	Coordination	One of our most important problems was the lack of coordination between the government and the officials in the matter of information. There were several voices and no coordination between different sources in the health department. (Man, 58 years old)

For peer review only

Institutional/provincial design	Knowledge exchange	In the discussion of pandemic management, the provinces were left to their own devices, and no province was aware of the activities of other provinces in the management of the infodemic. (Man, 55 years old)
**Information**		
**Theme**	**Sub-theme**	**Sample quotes**
Surveillance	Timeliness	No real-time data were available about the mortality of COVID-19 cases. Even now, if we refer to the statistical systems, we will not get a correct and reliable information and coordination so that we can inform the community. (Man, 48 years old)<br><br>Unfortunately, many of the events that happened in the country have not been documented and, for example, we do not know how many patients were infected with Corona, how many died. Even the medical and nursing systems used to come and give general information. It showed that either they don't have this information or they don't want to publish it.(Man, 49 years old)
	Data quality	No consensus data were available as mortality data reported by the hospitals, forensic systems, and cemeteries, were very contradictory. (Man, 48 years old)
	Surveillance systems	Surveillance data were extremely out of date, with low quality and consistency. (Man, 54 years old)<br><br>Our surveillance data were not accurate and real-time. (Man, 48 years old)<br><br>Therefore, it seems that we need a system that collects information from the environmental levels in a database in the form of software that has the power of analysis to give us alarms in different places. Its infrastructure in the country is planned as a syndromic care system, but I don't know if it has actually been implemented. (Man, 49 years old)
Knowledge translation	Evidence-informed policy making	No evidence about the effectiveness of interventions were synthesised and published for decision making. (Man, 54 years old)<br><br>The next problem was that the correct information did not reach those who should manage infodemic, for example, the number of patients at any moment, the number of deaths, what was the cause of death. (Man, 52 years old)
	Knowledge translation Capacity	Most of the statistics and information will be based on taste and subjective and this will cause individual perceptions and people will allow themselves to give any statistics. (Man, 48 years old)
Health workforce		
**Theme**	**Sub-theme**	**Sample Quote**
Capacity building	Diversity and flexibility	That's why we have to find an entry in the educational fields and teach this issue seriously in the form of workshops for groups close to graduation or students in the form of refresher courses regularly and continuously. Let's define a retraining unit for it and implement it operationally, not just theoretically. (Man, 48 years old)
Challenges	Limited resources	One of our most important challenges in risk communication and infodemic management is the lack of trained and expert people in this field. (Man, 70 years old)

For peer review only

Engagement	Community involvement	The non-participation of experts from different scientific fields in the pandemic management and informing the people was a big challenge, which caused people who had good experience or knowledge to be ignored, and therefore, unqualified people were in charge of informing the people. (Man, 42 years old)
**Financial resources**		
**Theme**	**Sub-theme**	**Sample Quote**
Government resources	Planning	Particular financial sources should be allocated to the risk communication activities. (Man, 66 years old)
**Media**		
**Theme**	**Sub-theme**	**Sample Quote**
Communication channels	Mass media	National TV and Radio channels are still the biggest and most influential communication channels in Iran. (Man, 46 years old)
	Government & health authorities' websites	We used both video media such as radio and television, as well as written media such as magazines, newspapers, and government websites, which were very active during the Corona era, to communicate the data related to the incidence of the disease in the province and recommend preventive measures. (Man, 49 years old)
	Social media platforms	We have established a social media platform (Instagram) named Dr+ to communicate with people and held online discussion panels with contribution of clinical physicians to address the community's needs and concerns. (Man, 53 years old)
Trust	Source credibility	Communication channels should be used that are highly credible and people trust to them. Some brand communication channels should be developed to maximize the impact and penetration of information among people. (Man, 47 years old)
spokespersons	Competence	For a person to be a spokesperson and to give information, to know how to give information, not to be too hopeful, not to speak too hopelessly, this is real information when we say not only to report numbers... For example, when the pandemic came, someone said that there is nothing, someone said Wow, we are unfortunate, which one of these people should accept when they look at it? (Man, 58 years old)
**Community**		
**Theme**	**Sub-theme**	**Example Strategies**
Diversity	Social context	The penetration rate of scientific issues in our society is low, which is related to various issues, so if we ever want to increase this penetration rate, we have to approach from different social and cultural aspects. (Man, 54 years old)
Engagement & empowerment	interventions	We have developed a community engagement facility to listen the community's needs and expectations and answer to their concerns and questions interactively. (Man, 55 years old)<br><br>Safiran-e-Salamat was a group of trained volunteers who communicate health information with their families and their neighbourhood. (Man, 55 years old)

For peer review only

Trust	Trust to government and health officials	Public opinion has no trust in the government, especially in matters that are officially announced. (Man, 47 years old)<br><br>Anyone, any scientist, any distinguished person, any accepted person comes and says something, the first time people do not accept it, especially if it is actually what the government says or emphasizes, people will definitely look for the opposite and say that there is something fishy about it. (Man, 53 years old)

For peer review only

Standards for reporting qualitative research (SRQR) checklist

No	Topic	Item	Page number
		Title and abstract	
S1	Title	Concise description of the nature and topic of the study Identifying the study as qualitative or indicating the approach (e.g., ethnography, grounded theory) or data collection methods (e.g., interview, focus group) is recommended	1
S2	Abstract	Summary of key elements of the study using the abstract format of the intended publication; typically includes background, purpose, methods, results, and conclusions	1
		Introduction	
S3	Problem formulation	Description and significance of the problem/phenomenon studied; review of relevant theory and empirical work; problem statement	2
S4	Purpose or research question	Purpose of the study and specific objectives or questions	3
		Methods	
S5	Qualitative approach and research paradigm	Qualitative approach (e.g., ethnography, grounded theory, case study, phenomenology, narrative research) and guiding theory if appropriate; identifying the research paradigm (e.g., postpositivist, constructivist/ interpretivist) is also recommended; rationale	4
S6	Researcher characteristics and reflexivity	Researchers' characteristics that may influence the research, including personal attributes, qualifications/experience, relationship with participants, assumptions, and/or presuppositions; potential or actual interaction between researchers' characteristics and the research questions, approach, methods, results, and/or transferability	-
S7	Context	Setting/site and salient contextual factors; rationale	4
S8	Sampling strategy	How and why research participants, documents, or events were selected; criteria for deciding when no further sampling was necessary (e.g., sampling saturation); rationale	4

For peer review only

S9	Ethical issues pertaining to human subjects	Documentation of approval by an appropriate ethics review board and participant consent, or explanation for lack thereof; other confidentiality and data security issues	4
S10	Data collection methods	Types of data collected; details of data collection procedures including (as appropriate) start and stop dates of data collection and analysis, iterative process, triangulation of sources/methods, and modification of procedures in response to evolving study findings; rationale	4-5
S11	Data collection instruments and technologies	Description of instruments (e.g., interview guides, questionnaires) and devices (e.g., audio recorders) used for data collection; if/how the instrument(s) changed over the course of the study	4-5
S12	Units of study	Number and relevant characteristics of participants, documents, or events included in the study; level of participation (could be reported in results)	NA
S13	Data processing	Methods for processing data prior to and during analysis, including transcription, data entry, data management and security, verification of data integrity, data coding, and anonymization/deidentification of excerpts	4-5
S14	Data analysis	Process by which inferences, themes, etc., were identified and developed, including the researchers involved in data analysis; usually references a specific paradigm or approach; rationale	4-5
S15	Techniques to enhance trustworthiness	Techniques to enhance trustworthiness and credibility of data analysis (e.g., member checking, audit trail, triangulation); rationale	5
Results/findings			
S16	Synthesis and interpretation	Main findings (e.g., interpretations, inferences, and themes); might include development of a theory or model, or integration with prior research or theory	5
S17	Links to empirical data	Evidence (e.g., quotes, field notes, text excerpts, photographs) to substantiate analytic findings	6-appendix 3
Discussion			
S18	Integration with prior work, implications, transferability, and contribution(s) to the field	Short summary of main findings; explanation of how findings and conclusions connect to, support, elaborate on, or challenge conclusions of earlier scholarship; discussion of scope of application/ generalizability; identification of unique contribution(s) to scholarship in a discipline or field	11
S19	Limitations	Trustworthiness and limitations of findings	13
Other			

For peer review only

S20	Conflicts of interest	Potential sources of influence or perceived influence on study conduct and conclusions; how these were managed	14
S21	Funding	Sources of funding and other support; role of funders in data collection, interpretation, and reporting	15

For peer review only

Health risk communication and infodemic management in Iran: Development and validation of a conceptual framework

Journal:	*BMJ Open*
Manuscript ID	bmjopen-2023-072326.R1
Article Type:	Original research
Date Submitted by the Author:	11-May-2023
Complete List of Authors:	Bazrafshan, Azam; Kerman University of Medical Sciences, HIV/STI Surveillance Research Center, and WHO Collaborative Center for HIV Surveillance, Institute for Futures Studies in Health,<br>Sadeghi, Azadeh; Kerman University of Medical Sciences, HIV/STI Surveillance Research Center, and WHO Collaborative Center for HIV Surveillance, Institute for Futures Studies in Health,; Kerman University of Medical Sciences, Deputy of Health, Department of Communicable Diseases<br>Bazrafshan, Maliheh Sadat; Kerman University of Medical Sciences, HIV/STI Surveillance Research Center, and WHO Collaborative Center for HIV Surveillance, Institute for Futures Studies in Health,<br>Mirzaie, Hossein; Kerman University of Medical Sciences, HIV/STI Surveillance Research Center, and WHO Collaborative Center for HIV Surveillance, Institute for Futures Studies in Health,<br>Shafiee, Mehdi; Kerman University of Medical Sciences, HIV/STI Surveillance Research Center, and WHO Collaborative Center for HIV Surveillance, Institute for Futures Studies in Health,; Kerman University of Medical Sciences, Deputy of Health, Department of Communicable Diseases<br>Geerts, Jaason; Canadian College of Health Leaders; City University of London, Bayes Business School<br>Sharifi, Hamid; Kerman University of Medical Sciences, HIV/STI Surveillance Research Center, and WHO Collaborating Center for HIV Surveillance, Institute for Futures Studies in Health,
**Primary Subject Heading**:	Public health
Secondary Subject Heading:	Health policy
Keywords:	COVID-19, Health policy < HEALTH SERVICES ADMINISTRATION & MANAGEMENT, INFECTIOUS DISEASES

SCHOLARONE™
Manuscripts

I, the Submitting Author has the right to grant and does grant on behalf of all authors of the Work (as defined in the below author licence), an exclusive licence and/or a non-exclusive licence for contributions from authors who are: i) UK Crown employees; ii) where BMJ has agreed a CC-BY licence shall apply, and/or iii) in accordance with the terms applicable for US Federal Government officers or employees acting as part of their official duties; on a worldwide, perpetual, irrevocable, royalty-free basis to BMJ Publishing Group Ltd ("BMJ") its licensees and where the relevant Journal is co-owned by BMJ to the co-owners of the Journal, to publish the Work in this journal and any other BMJ products and to exploit all rights, as set out in our licence.

The Submitting Author accepts and understands that any supply made under these terms is made by BMJ to the Submitting Author unless you are acting as an employee on behalf of your employer or a postgraduate student of an affiliated institution which is paying any applicable article publishing charge ("APC") for Open Access articles. Where the Submitting Author wishes to make the Work available on an Open Access basis (and intends to pay the relevant APC), the terms of reuse of such Open Access shall be governed by a Creative Commons licence – details of these licences and which Creative Commons licence will apply to this Work are set out in our licence referred to above.

Other than as permitted in any relevant BMJ Author's Self Archiving Policies, I confirm this Work has not been accepted for publication elsewhere, is not being considered for publication elsewhere and does not duplicate material already published. I confirm all authors consent to publication of this Work and authorise the granting of this licence.

For peer review only

Health risk communication and infodemic management in Iran: development and validation of a conceptual framework

Authors: Azam Bazrafshan[1], Azadeh Sadeghi[1,2], Maliheh Sadat Bazrafshan[1], Hossein Mirzaie[1], Mehdi Shafiee[1,2], Jaason M. Geerts[3,4], Hamid Sharifi[1*]

1 HIV/STI Surveillance Research Center, and WHO Collaborative Center for HIV Surveillance, Kerman University of Medical Sciences

2 Department of Communicable Diseases, Deputy of Health, Kerman University of Medical Sciences, Kerman, Iran

3 Honorary Visiting Fellow, Bayes Business School, University of London, UK.

4 Director of Research and Leadership Development, The Canadian College of Health Leaders, Ottawa, Canada.

*Corresponding author: Hamid Sharifi (Sharifihami@gmail.com)

Abstract

Objective: the COVID-19 pandemic exposed significant gaps in Iran's health systems. The accompanying infodemic undermined policy responses, amplified distrust in government, and reduced adherence to public health recommendations among the Iranian population. This study aimed to develop a conceptual framework for health risk communication and infodemic management (RCIM) during epidemics and health emergencies in Iran that could have potential applications in other contexts.
Design: this study was designed in two phases. Phase 1 involved semi-structured qualitative interviews with key informants to explore effective RCIM strategies across public health settings in Iran and to develop a conceptual framework. Phase 2 involved revising the framework based on feedback from an online expert panel regarding its comprehensiveness and validity.
Setting: provincial/national public health settings in Iran.
Participants: twenty key informants from provincial and national public health authorities who contributed to COVID-19 response programs participated in interviews. Nine experts from diverse academic disciplines, provincial and national settings, and geographical locations participated in an online expert panel.
Results: the conceptual model was created based on qualitative interviews and expert panel discussions and was structured according to four pillars of the World Health Organization (WHO) health system framework: leadership and governance, information, health workforce, and financial resources, along with media and community. Leadership and governance, including trustworthy leaders, were recommended as the foundation for developing RCIM in Iran. Developing an official strategy with information infrastructures, including high-quality surveillance systems, identified personnel and training for specialists among the health workforce, financial resources, communication channels, and community engagement, were recognised as other dimensions for developing health risk communication in Iran.
Conclusion: the proposed framework represents a step toward establishing a national health risk communication strategy in Iran. Further validation of the conceptual framework and experiments on how it could potentially influence policy and practice are recommended.

For peer review only - http://bmjopen.bmj.com/site/about/guidelines.xhtml

Keywords
risk communication, leadership, infodemic, COVID-19, emergency preparedness, crisis response

Strengths and limitations of this study

- This study consolidates insights from the field experiences of public health professionals across provincial and national settings in risk communication and infodemic management (RCIM) during the COVID-19 pandemic in Iran. Investigating the experiences and perceptions of academics, health professionals, and policy-makers enhances the validity of the results by including diverse perspectives on the topic of RCIM, and strengthens the proposed framework's credibility by providing a comprehensive understanding of its applicability in provincial and national public health settings.
- This study presents a novel conceptual framework, validated through full consensus by a panel of experts, for risk communication and infodemic management during epidemics and health emergencies in Iran.
- The qualitative nature of our study and the focus on one country may limit the perceived validity, however, involving two phases of diverse experts increases the potential relevance of the framework to other contexts

Introduction

The COVID-19 pandemic has changed our world, having affected every sector significantly, including health, education, economic, social, cultural, and informational (1). One of the social repercussions of the pandemic has been the constant spread through various media of overwhelming volumes of information, particularly concerning health, public health, government directives, and related issues (1). Much of this has been "misinformation" and "disinformation", both of which refer to incorrect or misleading content, the difference being the intentionality of those engaging in disinformation to cause harm, whereas misinformation is non-malicious but still potentially dangerous (1). Sources of mis- and disinformation range from non-stringently reviewed rapid academic publications with non-credible or flawed methodologies - and thereby dubious conclusions (misinformation), to "fake news" through anonymous social media posts and intentionally misleading messaging by government officials (disinformation) (1).

The COVID-19 pandemic and its accompanying infodemic have globally impacted individual and population health (2,3). In Iran, there is evidence, though limited, that the mis- and disinformation – the infodemic - spread widely through social media during the pandemic was associated with significant COVID-19 vaccine hesitancy rates (2,3), substantial uptake of traditional and complementary medicine products (4), and poor adherence to preventive measures, such as masking, in the general population (5). This escalation reinforces the importance of infodemic management in Iran.

False information, combining accidental and intentional, has contributed significantly to misguided health policies and to a host of deleterious consequences for individual and population health (6,7). This phenomenon is called an "infodemic". The World Health Organization (WHO) defines an infodemic as, "the widespread distribution of false or misleading information in digital and physical environments during a disease outbreak" (8). Without robust systemic safeguards in place, an infodemic can make communities, jurisdictions, and whole populations more vulnerable to disease infection and their side effects, as well as to other related harms (9). Information overload, including the infodemic, during the COVID-19 pandemic has represented a parallel pandemic whose transmission rate is much faster than the disease itself, since rampant erroneous and prejudicial information can trigger the spread of wild

For peer review only - http://bmjopen.bmj.com/site/about/guidelines.xhtml

and accelerated waves of fear and defiance in the general population (10). In Iran, for example, there is evidence, though limited, that the infodemic spread widely through social media during the pandemic was associated with significant COVID-19 vaccine hesitancy rates (2, 3), substantial uptake of traditional and complementary medicine products (4), and poor adherence to preventive measures, such as masking, in the general population (5). This escalation reinforces the importance of infodemic management in Iran.

Before the Internet, one of the main reasons for deaths during epidemics and pandemics was the lack of sufficient information on the prevention, care, and treatment of the disease (11).

But as technology advances, during health emergencies, the profusion of information, which is often conflicting, increases, primarily through social and digital media and instant messaging (12). The potential consequences of this profusion can intensify, particularly when people are in lockdown or isolation (12). Infodemics can severely change a pandemic's course by undermining public health and government recommendations and by diminishing population and community adherence to public health interventions such as, masking, social distancing, and vaccination. In particular, the impact of the infodemic on vaccination is critical because it is key to re-establishing pre-pandemic normalcy (13, 14). Economically disadvantaged countries are at higher risk of infodemics than developed countries, due to a range of inequalities (15). Lower rates of health literacy, limited access to reliable health information, and minimal public trust in public health authorities (15) can make people from underdeveloped and developing countries more susceptible to fake news and misinformation (13, 14). This vulnerability is compounded by further inequalities in terms of comparatively limited healthcare infrastructure and reduced access to healthcare facilities and public health professionals, which make people from these countries more prone to sporadic and ill-advised health and public health behaviours (16). In this context, the spread of misinformation and disinformation as part of the infodemic can pose a greater threat to populations in underdeveloped and developing countries during epidemics and health emergencies (17) by negatively influencing public risk perceptions and by undermining evidence-based policy creation and national and regional emergency responses (18-20). These hindrances can increase the spread and burden of the pandemic and widen global health disparities.

Infodemics have become a global phenomenon, impacting citizens in every country (21, 22). Addressing them is a new challenge and priority in managing and responding to epidemics and health emergencies. To understand and counter the rapidly changing nature of the COVID-19 infodemic and to mitigate its negative effects, such as the further spread of misinformation, several novel strategies and initiatives have been established across public health settings globally. The WHO has been widely respected for developing highly credible guidelines and initiatives to combat misinformation and infodemic management across the world (23). From early in the COVID-19 response, the WHO began to develop international strategies for infodemic management, in cooperation with other organisations, including the US Center for Disease Control and Prevention (CDC) and the Africa Centre for Disease Control and Prevention. To track and address misinformation surrounding COVID-19 and HIV, UNAIDS and the Africa CDC have been operating a rumour management system—software that uses machine learning, combined with human expertise, to collect and analyse rumour data from open-source traditional media (web-based, news broadcasts), as well as social media (Facebook, Twitter, WhatsApp). The system enables the identification of false and misleading information related to COVID-19 and HIV (23). In addition, the WHO developed a framework for infodemic management through crowd-sourcing and

For peer review only - http://bmjopen.bmj.com/site/about/guidelines.xhtml

online consultation with a wide range of global public health professionals (24). Ghana Health Services (GHS), together with the United Nations International Children's Emergency Fund (UNICEF) Country Office, have developed a systematic process that effectively identifies, analyses, and responds to COVID-19 and vaccine-related misinformation in Ghana (25). These initiatives are helpful foundations for further infodemic management strategies.

Risk communication and infodemic management (RCIM) are the core of risk management and effective responses to epidemics and health emergencies (26). According to Eysenbach (2020), there are four pillars of infodemic management: information monitoring, building health and e-health literacy in the general population, consolidating and disseminating credible information, including by accelerating the academic peer-review process, to ensure accurate and timely knowledge translation, and minimising factors, such as political or commercial agendas, that can distort or distract from evidence-based guidance or strategies (27). Combating mis- or disinformation for populations is as critical as ensuring much-needed medical equipment and supplies for health workers are readily available (28). In underdeveloped and developing countries, given their existing health information inequalities and public health vulnerabilities, customized RCIM approaches are needed to combat infodemics and to reduce their effects on population health (29). In particular, engagement and collaboration with local communities and leaders and stricter public health regulations are necessary (29). While some contexts may be more susceptible to the dangerous potential impacts of mis- and disinformation, none is immune, and the consequences of failing to tackle it directly and strategically can be dire.

The purpose of this study was to build on and extend previous conceptualisations of RCIM capacity building by creating a conceptual framework of RCIM in Iran. To achieve this, we applied a systems thinking lens, since the pandemic demonstrated that not only can health emergencies affect all people and sectors, but that addressing infodemics requires more than just public health messaging. Along with potential benefits for other sectors, robust national and regional RCIMs can have a significant positive impact on health systems, those who bear the brunt of health emergencies (30). The WHO describes a health system as a set of interconnected building blocks that are essential to health system functioning. The blocks are: service delivery, health workforce, health information systems, access to essential medicines, financing, and leadership/governance, with the latter being central to all (30). It is essential that each of these interconnected elements are addressed concomitantly in response to changing population health needs and inequalities, and to epidemics and health emergencies (30). This multifaceted understanding of health systems, along with considerations for other related sectors, is vital to effective RCIM strategies, since mis- and disinformation can affect those in all aspects of society. The nature of health emergencies requires that policy and communications strategy recommendations should be gathered from a diverse group of actors with relevant RCIM expertise, including researchers, educators, advocates, practitioners, funders, private sector representatives, community representatives, government officials, policymakers, and various trusted international experts and representatives. Leaders from across sectors should also collaborate with public health and with each other to integrate RCIM strategies effectively to improve the health of all people and communities (31, 32). Applying these diverse perspectives and the systems thinking approach can enhance RCIM policies, strategies, and activities nationally, regionally, and locally and can lead to improved relevant health outcomes during epidemics and health emergencies (33).

For peer review only - http://bmjopen.bmj.com/site/about/guidelines.xhtml

Drawing on the importance of this approach, we involved the perspectives of a diverse set of experts in our study to enhance the quality and reliability of the conceptual framework (34, 35). Our intention was for the framework to have the potential to be applied to build RCIM capacity effectively in Iran and in other underdeveloped and developing countries and beyond.

Methods

This sequential, mixed-methods exploratory study was conducted in two phases from October to December 2022. Phase 1 involved semi-structured interviews with key informants from provincial and national public health authorities to inform the creation of an initial framework of key RCIM components across settings. Phase 2 involved an online panel of experts from relevant scientific domains to validate the conceptual framework's validity, credibility, and transformability (34, 35). We then revised the framework based on the panel's feedback (Figure 1). This study followed the Standards for Reporting Qualitative Research (SRQR) checklist (36).

Phase 1: semi-structured interviews

Phase 1 involved semi-structured interviews with a purposive sample of 20 Iranian public health professionals across provincial and national health authorities. Study participants included stakeholders, academics, decision-makers, and leaders with expertise in community health, epidemiology, public health, social medicine, health communication, and sociology. Participants were from eight pre-specified provinces: Kerman, Tehran, Fars, Isfahan, Mazandaran, West-Azerbaijan, Kermanshah, and Sistan va Baloochestan. These provinces were initially selected to involve a representative sample of the Iranian population with diverse social, geographical, and cultural characteristics. Inclusion criteria were: 1) having at least one year of experience in either COVID-19 prevention and control programs or decision-making in provincial or national public health settings, and 2) willingness to participate in the study.

An interview guide was developed according to previous studies (Appendix 1). The interview guide focused on the processes, infrastructures, challenges encountered, and best practices relevant to RCIM during the COVID-19 pandemic in Iran. The interview guide was assessed beforehand by two expert reviewers. It was subsequently pre-tested with three target population members before the implementation.

The interviews followed a semi-structured design, allowing for variations of the order of the questions and follow-up questions based on participant responses. The objectives and the activities that were involved in the study were explained to the participants. The principal investigator's contact details were provided, and participants' confidentiality was guaranteed. Written consent was sought before the interview, and the participants were asked to email the completed form to the principal investigator (Appendix 2). An experienced interviewer with a background in qualitative research and interviewing expertise conducted the interviews in the Farsi language. Due to COVID-19 social distancing, all interviews were conducted by telephone, audio-recorded, and transcribed verbatim. Interviews ranged between 20–55 minutes (mean = 34 minutes). Interviews lasted until the researchers realised they had reached content saturation.

To analyse the interview data, we applied Braun and Clarke's framework for thematic analysis of qualitative data (37) to the interview transcripts. The authors define thematic analysis as, "the process

For peer review only - http://bmjopen.bmj.com/site/about/guidelines.xhtml

of identifying patterns or themes within qualitative data" (p. 78). Their framework involves six steps: becoming familiar with the data, generating initial codes, searching for themes, reviewing themes, defining themes, and writing up. We used MAXQDA 12 (VERBI GmbH, USA) for manual coding and content analysis.

Based on the themes identified from the qualitative interviews, we created an initial set of 33 key RCIM strategies and organized them according to four of the pillars of the WHO model of the health systems, along with media and community (30). This initial set of components served as the basis for discussion with, and validation by, the expert panel in Phase 2 and consequently, the conceptual model.

Phase 2: expert panel validation

Phase 2 involved a group of nine experts selected through purposive sampling to validate and prioritise key components of the initial RCIM model and to evaluate its completeness and validity (34, 35). The panel included a diverse set of stakeholders, academics, decision-makers, leaders from the various communities, and national public health leaders. The inclusion criteria for this phase were: 1) having at least three years of professional experience or established research expertise in the fields of public health, epidemiology, crisis management, infodemiology, social media studies, or health communication; and 2) willingness to participate in the study. Potential panel members (n = 9) were identified through their academic or professional roles in health risk communication or risk management activities across provincial or national health authorities during the COVID-19 pandemic. Prospective contributors were given a short statement of the study's purpose and design and were invited by email to participate in the panel discussion. During the discussion, panellists engaged based on their assessments of the initial conceptual model and suggested additions, deletions, and modifications, with the aim of informing a highly complete and credible model of essential components of an RCIM model for the country. As mentioned previously, this validation by experts was also intended to augment the quality, reliability, and validity of the model (34, 35).

Following this phase, several revisions were made to the original conceptual model, but no factor was deemed required for exclusion. The required level of consensus for each component in this phase was a minimum of 75% agreement.

Patient and Public Involvement Statement

No patients or community members were involved in this study.

Results

Phase 1: semi-structured interviews

Participants

Most participants were men (n=19, 95%), aged 51-60 years old (n=11, 55%), from medical and public health disciplines (n=17, 85%), who work as a provincial or national health officer (n=14, 70%). Participants were mostly from Tehran (n=7, 35%) and Kerman provinces (n=5, 25%) (Table 1).

Table 1: Demographic characteristics of the participants in the interviews (Phase 1)

For peer review only - http://bmjopen.bmj.com/site/about/guidelines.xhtml

Demographic characteristics	Frequency (%)
Residence at the time of interviews	
Tehran	7(35)
Kerman	5 (25)
Fars	2 (10)
Isfahan	2 (10)
Kermanshah	1 (5)
Mazandaran	1 (5)
Sistan va Baluchestan	1 (5)
West Azerbaijan	1 (5)
Age	
40-49	6 (30)
50-59	11 (55)
60≥	3 (15)
Gender	
Men	19 (95)
Women	1 (5)
Academic Discipline	
Epidemiology	6 (30)
General medicine	4 (20)
Sociology	3 (15)
Health policy	2 (10)
Infectious disease	2 (10)
Social medicine	2 (10)
Health education & promotion	1 (5)

The analysis of the qualitative data collected during the key informant interviews revealed 948 open codes and 84 sub-themes. Sub-themes were subsequently classified into 33 components (Appendix 3).

The next step involved organising these components according to six categories representing a combination of the WHO model (24) and key aspects of the Iranian health system: leadership and governance, information, health workforce, financial resources, media, and community. The results formed the initial RCIM conceptual model.

Theme 1: Leadership and Governance

Leadership and governance are at the heart of the WHO model of health systems (30) and Dr. Tedros Ghebreyesus, Director-General of the WHO, said in the early months of the pandemic, “The greatest threat we face now is not the virus itself, it's the lack of global solidarity and global leadership" (38). Similarly, in an international study of crisis leadership featuring 32 co-authors from 17 countries, Geerts et al. (2021) highlighted that effective leadership, trust in leaders through transparent decision-making, communication, and accountability are vital to successful public health strategies (34, 35).

These examples reinforce the finding in our study that every respondent mentioned leadership and governance as essential foundations for the RCIM model. Seven respondents emphasised transparency in decision-making, effective communication, and accountability as important characteristics of effective leadership and governance. According to these respondents, a lack of transparency and

For peer review only - http://bmjopen.bmj.com/site/about/guidelines.xhtml

accountability among Iranian health officials and government authorities were among the country's substantial weaknesses in risk communication and had adverse consequences. Thirteen respondents suggested that senior public health officials intentionally caused non-transparent information communication during the COVID-19 pandemic, motivated by financial and other competing interests, which, they suggested, eroded public trust significantly. Similarly, regarding sources of false messaging, seven respondents indicated that pharmaceutical companies were a major source of spreading misinformation during the pandemic. These respondents suggested that public health officials to allow their financial and competing interests, including those related to pharmaceutical companies, to deter them from spreading credible information about the efficacy of some new and underdeveloped medications and vaccines. One respondent expanded a perception that many health officials were among shareholders of the pharmaceutical industry, they advertised some drugs or public health products and subsequently caused a fake and unrealistic demand among the population.

Almost all respondents (n =18) emphasised that the health system needs a robust risk communication strategy and increased infodemic management capacity by developing infrastructures for monitoring the public's risk perception, knowledge and attitudes, communicating with the public, and providing clear guidance through various media based on the best available science. Increased RCIM capacity would enable early detection of outbreaks of potentially harmful mis- and disinformation, and quick responses to counter falsehoods with facts or other reliable information in a targeted way for each audience. One respondent suggested that building capacity should involve designing an infodemic management system that defines national and provincial responsibilities based on lessons learned from credible global guidelines, national and regional successful strategies, challenges, and failures, as well as leading practices, locally and elsewhere. This respondent added that the system should include a national independent core rapid response team with clear roles, protocols, and accountabilities to collaborate with communities to screen and identify their needs, concerns, and misinformation sources, to lead quick responses the potential risks, and to prevent or mitigate the viral spread of mis- and disinformation across the communities. Similarly, six respondents proposed developing, implementing, evaluating, and revising communication policies and strategies to confront potential risks. According to these respondents, the lack of national and provincial policies and programs for RCIM severely inhibited the national COVID-19 control and management efforts. Four respondents argued that the government's poor management of the COVID-19 infodemic, poor communication with the public and other stakeholders, and a lack of national and provincial strategies to address misinformation were major shortcomings of risk communication and infodemic management in Iran.

To optimize RCIM strategies, it is crucial to involve representatives from multiple sectors and the community representatives. Eleven respondents indicated that top-down public health initiatives that lacked community-based customisation and approaches were among the major barriers to acceptance of COVID-19 prevention and control interventions during the pandemic. Multi-sector and community involvement could also potentially improve community members' motivation to participate actively in information communication and management of infodemics. For example, one respondent described how social influencers in community-based approaches, such as that in Safiran-e-Salamat, Tehran, served as facilitators for effective risk communication and infodemic management across provincial settings.

For peer review only - http://bmjopen.bmj.com/site/about/guidelines.xhtml

Ineffective use of institutional and provincial infrastructures and capacities and lack of crowdsourcing were cited by four participants as major barriers to effective infodemic management during the COVID-19 pandemic. These respondents elaborated that medical universities and faculty within the provinces were isolated from the national health authorities and not supported by the Ministry of Health in planning and decision-making. These two respondents recommended establishing official networks of experts in diverse areas and practitioners to share experiences, challenges, and best practices of information communication during the potential risks and increase capacity.

Along with trustworthy public health guidance and recommendations, two respondents stated that providing all people with tools for filtering, assessing, and fact-checking information is essential to combat misinformation during the pandemic and health emergencies. Five respondents believed that using a well-known and reliable communication channel and technology-based interventions would maximise the spread of valid information and impact communication efforts and strategies.

Theme 2: Information

Developing a network platform to systematically collect, analyse, and interpret epidemiologic data from the community and quickly disseminate the key findings was considered an important characteristic of risk communication by fourteen respondents. These respondents emphasised that a lack of access to real-time, valid, and high-quality data about the incidence, mortality, and burden of the COVID-19 disease in different provinces intensified the potential risk and spread of misinformation among the population.

Similarly, seven respondents indicated that a lack of access to high-quality surveillance data for research activities and to inform responses to potential and emergent challenges reduced the reliability of information and recommendations and transparency of government decisions. Consequently, it raised dramatic social concerns about the government's ability to estimate the spread of the disease and to anticipate and evaluate the effect of specific policies on population health.

In addition to data quality issues, two respondents suggested that the lack of substantial resources to handle the multiplication of data sources and information producers, to monitor disease trends regularly, and to appraise the quality of data sources were major barriers to the effective use of surveillance data for decision-making during the pandemic.

One respondent stated that some politicians, health officials, and media misinterpreted and selectively reported data according to their own financial, commercial, and political interests, which he considered a major source of misinformation during the pandemic. Two respondents argued that effective knowledge translation of high-quality data is required to minimise the spread of misinformation across different sectors and communities, since people's political, commercial, and financial interests can lead them to distort scientific messages.

Finally, three respondents reported that these data issues contributed to a lack of evidence-based policies and practices, which severely inhibited effective risk communication and infodemic management.

For peer review only - http://bmjopen.bmj.com/site/about/guidelines.xhtml

Theme 3: Health workforce
All respondents highlighted the need for well-trained specialists in various organisations with a mix of skills that can contribute to risk communication and infodemic management activities, as well as additional training for all health workers.

Six respondents promoted the benefits of involving public health agencies, epidemiologists, data scientists, and sociologists who have unique expertise and credibility to guide policies, strategies, and risk communication and infodemic management, in collaboration with health workers. However, all respondents agreed that the Iranian scientists and experts have not helped substantially to prevent misinformation and to mitigate the effects of the infodemic. Further, three respondents suggested that, in some cases, scientists and academic experts in infodemic management were considered sources of misinformation, seen as contributing to the infodemic trends by publishing low-quality scientific papers and providing non-credible, sensational, or exaggerated information about new treatments.

To gather relevant data and to disseminate evidence-based guidance, twelve respondents highlighted the need to involve professional councils, NGOs, interested experts, and health volunteers as additional workforce sources to improve the speed and effectiveness of the response to the infodemic.

Three respondents identified a key gap in RCIM: a lack of qualified and well-trained spokespersons in public health and health organisations, which, they suggest, greatly diminished the quality of risk communication activities during the pandemic. Four respondents referenced a range of competencies necessary to improve the quality of the health workforce education and practice, which can be used to select potential candidates for RCIM roles and to design educational courses and curricula to enhance their ability to support health emergency response effectively.

Theme 4: Financing
Three respondents highlighted the importance of effective financial resource allocation to risk communication and infodemic management to support data collection and analysis and communication strategies. One respondent argued that multi-sector collaborations could reduce the risk of underfunding communication responses. Using technology-based interventions, such as text-messaging approaches, could improve the cost-effectiveness of communication strategies.

Theme 5: Media
All respondents mentioned characteristics related to media. Two respondents believed that given the broad coverage and penetration of radio and television (TV) as dominant communication channels in most parts of the country, involvement of trustworthy spokespersons in, and collaboration with, mass media, could improve the effectiveness of risk communication strategies. However, the respondents elaborated that the weak contribution of these media in RCIM was an obstacle to preventing misinformation. Even worse, nine respondents argued that TV and other mass media actually contributed to the COVID-19 infodemic. According to these respondents, broadcasting news reports that included misleading and low-value information, interviews with non-experts, and flagrant criticisms or debates about the performance of public health agencies reduced public trust and prompted many people and communities to rely more on informal and social media channels.

Three respondents added the need to improve government and health authorities' websites to disseminate real-time and high-quality information, since many consider them the source of credible

For peer review only - http://bmjopen.bmj.com/site/about/guidelines.xhtml

information. Additionally, six respondents advocated social media platforms as important communication channels for most communities to aid the acceptance of public health interventions. Three respondents elaborated that reduced public trust in formal and government communication channels caused many people to rely instead on social media platforms, viewing them as more trustworthy. For example, according to two respondents, the dissemination of valid and high-quality data through social media channels influenced the impact of local interventions and improved vaccination coverage for vulnerable and ethnic populations, particularly in Sistan-va-Baluchestan and West Azerbaijan. These respondents explained that, due to higher accessibility, social media platforms were highly utilised by younger adults and geographically distanced locations and, therefore, effective in improving the speed and effectiveness of interventions among members of these populations.

Theme 6: Community

Eight respondents reinforced the importance of involving the community in risk communication and infodemic management in two ways. First, by understanding their diverse demographic, social, economic, and cultural compositions and by identifying their information needs, preferred media, and key influencers. Second, by listening to their concerns, sharing key data and evidence-based recommendations with them, and incorporating their input transparently into important, relevant decisions. However, four respondents suggested that the lack of community-centred approaches reduced the effectiveness of risk communication efforts during the COVID-19 pandemic in most Iranian provincial settings. Three respondents recommended priority training in critical thinking, media, and health literacy for community leaders in risk communication and infodemic management to improve their engagement, active contribution, and effectiveness. According to these respondents, well-informed, engaged, and enabled communities can minimise misinformation and infodemic consequences and develop their own local solutions. One respondent expressed that this kind of respectful, reciprocal relationship with communities could rebuild and maintain public trust in public health agencies, health professionals, and government authorities and could also maximise social cohesion and local capacity successfully respond to potential risks during the crisis.

Phase 2: expert panel validation

In this phase, the completeness and trustworthiness of the proposed conceptual model of RCIM in Iran was discussed by the online expert panel until consensus was achieved by all panel members (100% agreement) (Figure 1).

Figure 1: a conceptual model of components and infrastructures of health risk communication and infodemic management system in Iran

Discussion

This study, conducted during the COVID-19 pandemic, was inspired by an awareness of two aspects of the global experience. The first is the extent to which infodemics can influence the course of large-scale health emergencies, given the global impact that the COVID-19 infodemic has had on individual and population health (2, 3). The term "infodemic" refers to the profusion of recurring waves of information of overwhelming volume and predominantly unclear and/or mixed credibility, including disinformation, messaging intended to deceive. Infodemics can erode the quality and effectiveness of policy and strategy decisions. They can also intensify community and population-level distrust in government and public health officials and experts, including their recommendations, which can drastically undermine national and local efforts to effectively mitigate the spread of the disease. As people's faith in official

For peer review only - http://bmjopen.bmj.com/site/about/guidelines.xhtml

sources diminishes, the likelihood of them being influenced by alternatives increases, and the escalation of rumours and fear exacerbates. Broadcasts of incorrect information through TV, radio, newspapers, and other mainstream and social media, and even through academic publications, can contribute to widespread non-adherence to public health directives, thereby perpetuating the spread, impact, and burden of a pandemic.

Infodemics can have increasingly devastating effects in economically disadvantaged countries, due to a wide range of inequalities (15), which can make people local populations more susceptible to fake news and misinformation (13, 14). This vulnerability is compounded by further inequalities in terms of healthcare infrastructure, access to healthcare facilities, and health professionals (16). Evidence suggests that, in Iran, the infodemic spread, largely through social media, contributed to several adverse outcomes in the general population (32). The speed, scale, and potential lethal consequences of infodemics are why they are considered parallel pandemics, which require a dedicated, strategic, expertise-informed response to allay.

The second inspiration for the study was an appreciation for the vital mitigating role that effective risk communication information management (RCIM) can play in pandemic and infodemic response. Understanding the sources of mis- and disinformation and rapid, effective government and public health response, in collaboration with multi-sector and community leaders, to evolving risks, along with targeted strategies, can mitigate potential negative ramifications.

The purpose of this research was to support increased national and local RCIM capacity in Iran and beyond by creating a unique conceptual model of evidence-, expert-, and experience-informed strategies for RCIM during epidemics and health emergencies. To create the model, we applied a systems thinking lens, since infodemics and their effects reside within multi-sectoral complex systems involving interactions and actors from all aspects of society. This perspective considers how to most effectively engage with potential audiences and diverse stakeholders, including the community, scientists and experts, government and public health officials, health workforce, pharmaceutical industries (private sector), and others, through physical and virtual communication channels (Figure 2). This comprehensive approach can enhance the potential for sectoral and provincial health authorities to improve RCIM activities and relevant health outcomes during epidemics and health emergencies. Given this perspective, following leading international pandemic research (27), we gathered two stages of input and validation from diverse groups of those with expertise and experience in public health and various related sectors and disciplines.

Figure 2 Components of the COVID-19 risk communication and infodemic management in Iran, source: own production

The model presented here is organised according to four of the pillars of the WHO model of the health systems, along with media and community (30), and it is reinforced by the full consensus of an expert panel in terms of its quality, completeness, and validity. While the model was developed for the Iranian context, the intention was for it to have potential application in other contexts to decrease the spread and burden of future health emergencies and to minimise global health disparities.

For peer review only - http://bmjopen.bmj.com/site/about/guidelines.xhtml

What follows are some insights on, and priority points for, effective RCIM that emerged from the qualitative interviews and expert panel discussions.

Our findings support the vital importance and potential impact of establishing a robust, integrated, evidence-informed national RCIM strategy, with regional applications, to strengthen existing RCIM capacities to explore, track, monitor, respond, and adapt to the needs of each community. Our results also show that effective RCIM requires several essential components: an official RCIM strategy supported by dedicated personnel, infrastructure, financing, and resources, trustworthy leadership and governance, the expertise and capacity to inform policies and to gather, analyse, and communicate the best available information in real-time, effective messaging through mainstream and social media with local support, RCIM training for specialists among the healthcare workforce, and community engagement to maximise local outcomes.

Official RCIM strategy with dedicated personnel, infrastructure, financing, and resources

Effective RCIM requires having an official strategy, based on a credible conceptual framework, which drove this study, and consolidated lessons learned locally and elsewhere. Aspects of the strategy need to evolve and adapt based on changing circumstances and it is essential that consideration is given to roles and customised approaches at the national, regional, and community levels. This should involve an official core national rapid response team with clear roles, protocols, resources, and accountabilities, along with regional chapters.

Second, the strategy needs to be supported by the infrastructure, financing, and resources to operate effectively. Respondents in our study suggested that in Iran, however, funding to enhance RCIM system capacity in terms of infrastructure and personnel is poor and they indicated that the lack of direct funding hindered the risk communication support during the pandemic. Although there are media and public communication experts, the number of those available with expertise and training in responding to major health risks is critically limited. Underfunding RCIM appears to be a common challenge in many countries. Evidence from south-east Asia (39), for example, revealed that during the COVID-19 pandemic, few countries allocated resources to emergency risk communication. However, some specific areas have budgets, such as information education communication materials. Also, resource mobilisation and the use of non-governmental resources were reported as strategies to address this critical challenge within the country's national and provincial settings. Priority areas and optimal mobilisation and use of resources is an important consideration for further exploration.

Leadership and governanc

The COVID-19 pandemic has highlighted the global importance of trustworthy and effective leaders who keep people at the forefront of their decisions, which they make transparently based on the best available evidence from a systems thinking perspective, and hold themselves accountable for outcomes (27). Leadership and governance are also at the heart of the WHO model of health systems (30). Similarly, every respondent in our study reinforced the fundamental importance to effective RCIM of leadership and governance.

Leadership-wise, effective RCIM response involves ensuring that the official RCIM strategy, personnel, infrastructure, and resources identified in the previous point are in place. But these are insufficient on their own.

For peer review only - http://bmjopen.bmj.com/site/about/guidelines.xhtml

Effective RCIM leadership and governance depends on government officials and public health and other leaders earning people's trust through their integrity and public- versus self-interest. If either of these are considered compromised, RCIM efforts are vastly undermined, as was seen during the pandemic in Iran. Leaders also earn trust by instilling confidence that, in a timely manner, they have the expertise and capacity to access and interpret the most credible information, operationalise an evidence-informed strategy and adapt it when necessary, and make and communicate transparent decisions, along with their rationale. Credible information should be actively gathered from many sources, including international, national, and local experts, leaders in all related sectors, and community leaders and representatives. Effective leaders understand that tailored, two-way communication according to an accurate understanding of each stakeholder's and community's preferences is crucial. This communication involves asking important questions, active listening, sharing information, providing clear recommendations, tools, and customised messaging, and engaging local support to lead RCIM. Finally, respondents indicated that leaders need to hold themselves publicly accountable for outcomes.

Leaders' ability to deliver on their responsibilities requires the aforementioned strategy, personnel, infrastructure, and resources, as well as developing a network of diverse international, national, and local experts in various relevant disciplines, leaders from all sectors, RCIM specialists within the health workforce, and community leaders.

Information

Effective RCIM relies on three approaches to information. The first is the expertise and capacity to, in a timely manner, proficiently screen, monitor, and verify the validity, relevance, and potential impact of available information from official and unofficial sources. The second is the ability to actively gather information from those with relevant expertise related to pandemic response and to RCIM strategies. The third is to communicate the most credible information to inform policymakers, government officials, public health, community leaders, and health and healthcare practitioners to equip them with the knowledge to create, implement, and adapt appropriate and effective strategies.

Media and communications

Combatting infodemics hinges on credible and strategic messaging through official sources, including government and public health websites, as well as through mainstream and social media, in collaboration with local representatives. The collaborative contribution of the government, public health, leaders in various sectors, experts, and community leaders in circulating health information is a key strategy to counter mis- or disinformation during health emergencies. Understanding the needs, perceptions, priorities, and concerns of key stakeholders across public and private settings and identifying different opportunities and strategies for their involvement are critical steps to developing and implementing risk communication policies and strategies.

Developing or sustaining reputed and well-trusted communication channels is critically required to maximise the effectiveness and impact of communication strategies. How the community perceives various epidemics and health emergencies, what they perceive to be their role, how they are influenced, and how their views tally with the biomedical approach, are not entirely investigated in the country.

For peer review only - http://bmjopen.bmj.com/site/about/guidelines.xhtml

For peer review only

According to our findings, a lack of public trust in mass media and government channels directed Iranian citizens to the wide use of online social networks. Due to the dramatic reduction in social capita, most Iranians distrust governmental information sources, and this fact challenged the community's compliance with preventive behaviours (COVID-19 vaccination) during the COVID-19 pandemic. Lack of trust in the government as a source of information was reported globally in the existing literature. According to recent evidence, only 40% of the European citizens from the Economic Co-operation and Development countries participated in a survey and trusted their governments as sources of information about the Corona Virus (40). False claims about the activities, statistics, or policies of public and government authorities were reported as a major source of disinformation during the COVID-19 pandemic, suggesting that "governments have not always succeeded in providing clear, useful, and trusted information to address pressing public questions" (41). Meanwhile, disinformation and claims may also be falsely attributed to official and governmental sources, amplifying this problem. In this regard, delivering truthful, evidence-informed, and compelling information to various audiences through their preferred channels and understanding behavioural and psychological biases is recommended. This is especially important for young audiences, who tend to access news and information predominantly via social media platforms(42). It is, therefore, a critical issue for health risk communication and infodemic management to ensure key factual messages reach all audiences. It is also important to effectively leverage the channel through which various audiences are relayed since different groups are likelier to trust media outlets that align with their views.

RCIM training for health workforce

While some capacity-building workshops for health professionals were held during the COVID-19 pandemic by the Ministry of Health and medical universities, they were largely been ad hoc, of short duration (less than a week), and of variable quality. Those trained have often been public health professionals who then move on to other areas of public health. A planned and institutionalised approach to capacity-building is required to have an adequate pool of trained experts for epidemics and health emergencies. Therefore, financial resources and building risk communication expertise are critical priorities for the country. Obtaining both these resources will require the endorsement of senior policymakers. Advocacy to policy-makers and key decision-makers on the role and impact of RCIM is very important.

Training

RCIM is a broad and multi-disciplinary field involving health communication, health education, public affairs, behaviour change communication, and social mobilisation. It is therefore required to build the capacity of key contributors to verify, filter, and curate health information and use diverse communication channels to target public audiences (43). Community-based organisations, patient advocacy groups, professional associations, and non-governmental organisations with reputable brands, organisational resources, and a network of relationships can be leveraged to improve health risk communications. Existing evidence demonstrates that by partnering with local public health experts and policymakers to create information hubs and community outreach programs (44), these groups can significantly improve their ability to serve the information needs and concerns of diverse communities while also advocating for policy solutions. Existing evidence demonstrates that involving community members as planners, and attendees in pre-crisis planning activities, leads to increased preparedness and response activities. Therefore training in roles and responsibilities, relationship building, and team-

For peer review only - http://bmjopen.bmj.com/site/about/guidelines.xhtml

building are required strategies to facilitate and strengthen the contribution of community-based organisations, expert associations, and other relevant partners during epidemics and health emergencies (45).

Community engagement

Effective RCIM depends on engaging with communities to share information and to understand their unique concerns, experiences, wisdom, available resources, and preferred forms of communication, as well as to earn the support of community leaders as key intermediaries in response. These measures can maximise community collaboration and receptivity to ensuing recommendations. Given the social, contextual, economic, and geographical diversity that exists within countries, customised, community-based approaches essential for RCIM and health emergency response. Ethnographic and anthropological/social research on epidemics and health emergencies in the country could also help to improve understanding of the acceptability of response to emergencies and public health interventions. According to our interviews and expert panel discussion, the community was considered a missing piece in RCIM strategies in Iran. Information needs and concerns (e.g., disabilities, gender, age, literacy, cultural/ethnic backgrounds, access to technology) of the general Iranian population remained unexplored. In addition, the participatory engagement of citizens in a collective response to the COVID-19 infodemic was not only insufficient, but rather, at times, it was discouraged.

During the COVID-19 pandemic, national health authorities and governments in most countries predominantly demonstrated top-down communication strategies (46). Effective RCIM requires a whole-of-society effort to sustain a healthy information ecosystem. Understanding the needs and concerns of vulnerable groups who might experience barriers to accessing accurate health information, care, and support, or be at higher risk of exposure and secondary impacts, such as children and adults with disabilities, is critically important(46). Effective risk communication can save lives during epidemics and health emergencies; however, existing evidence revealed that inadequate risk communication resulted in high exposure and loss of lives, as seen in Iran and Italy in the first wave (46, 47). Training and advising the general population on how to consume and share health information responsibly may be an effective strategy to improve the engagement and participation of public communities in risk communication and infodemic management. Investing in the community's media literacy, health literacy, and critical thinking skills before the crisis can prepare society to mitigate the physical and emotional consequences of false news and disinformation and increase resilience (48). As disinformation and infodemic during epidemics and health emergencies undermine trust, amplify fears, and consequently affect countries' responses to the global pandemic, tailored strategies to build and maintain trust among the public community are of utmost importance. Therefore, to be effective and foster public trust in government, any activities conducted in health risk communication and infodemic management must be guided by the principles of transparency, integrity, accountability, and community participation.

Limitations

We address some limitations of the study. First, given that our study and the novel conceptual framework presented here are the first to address comprehensively the RCIM needs of, and strategies for, the Iranian health system context, further research and validation of its completeness and reliability, particularly after attempts to implement it, would be useful. Similarly, investigating causality and replicating the study with identical results can be challenging with qualitative studies of complex

For peer review only - http://bmjopen.bmj.com/site/about/guidelines.xhtml

phenomena. However, involving diverse sets of respondents with experience and expertise in leading RCIM in two phases of research before reaching total consensus heightens the potential for the framework to be considered credible and effective in being applied in the Iranian context. Further research could focus on applying best practices in RCIM, ecosystem mapping and analysis, and strengthening data collection and analysis for monitoring, evaluation, and learning. Investigating specific methods for evaluating RCIM activities are also important and critically recommended. Second, by focusing on the Iranian context, the transformability of the framework to other contexts remains yet untested. However, the high-level results echo leading international research on effective pandemic response and even if regional customisation would be beneficial, the current framework could potentially represent a well-informed basis for discussion, for further research, and for the creation of local versions.

Conclusion

This study was inspired by an appreciation for the extent to which the COVID-19 infodemic is reported to have impacted the spread and burden of the disease globally, and of the role that an effective risk communication and information management (RCIM) strategy can play in mitigating the impact of infodemics. The purpose of this research was to support increased RCIM capacity in Iran and beyond through the creation of a unique conceptual model of evidence-, expert-, and experience-informed strategies for RCIM during epidemics and health emergencies. Our findings suggest that ineffective RCIM impeded the emergency response in Iran’s COVID-19 management, which is partly attributable to Iran’s government and national public health authorities failing to infuse an evidence-informed and strategic RCIM into policy- and decision-making. Consequently, access to high-quality and real-time information was extensively restricted and not publicly available, and the provincial public health settings failed to establish effective community relationships with experts, researchers, professional councils, and NGOs to facilitate knowledge translation and utilisation. Further, the extensive use of social media platforms and mass media worsened the circulation of rumours, fake news, and disinformation and led to public distrust. The lessons learned from the outbreak management and response in Iran suggest that RCIM should be an essential component of health emergency readiness and response activities. This begins with trustworthy leaders at all levels who have integrity and make credible, transparent decisions, and hold themselves accountable for outcomes. A national RCIM program should be established to support the required infrastructures, personnel, and processes to address communication challenges during epidemics and health emergencies. This should be based on a conceptual model of RCIM to illustrate a collaborative and interdependent context of risk communication activities, implying that any improvements in these areas requires an integrated and holistic approach. The government, private sector and pharmaceutical industries, experts, and the public should be involved in time, contributing diverse views and fulfilling respective responsibilities. The conceptual model presented here has the potential to be either implemented or serve as the foundation for the creation of a similar model in other contexts. Sharing experiences, challenges, and leading practices among jurisdictions can further improve the reliability and credibility of guidance and strategies.

For peer review only

For peer review only - http://bmjopen.bmj.com/site/about/guidelines.xhtml

Ethics approval and consent to participate

This study was approved by the Research Ethics Committee of the Kerman University of Medical Sciences (**IR.KMU.REC.1400.379).** The Declaration of Helsinki was followed and informed consent was obtained from participants before starting the data collection stage.

Availability of data and materials

The datasets used and/or analysed during the current study are available from the corresponding author upon reasonable request.

Competing interests

All authors declared that they had no competing interests.

Funding
This study was financially supported by World Health Organization-Regional Office for the Eastern Mediterranean (WHO-EMR) Call for Proposals for Special Grant for COVID-19 Research, 2022 (**WHO Reference: 2022/1291032-0**)

Authors' contributions

AB contributed to the project concept and manuscript design, qualitative data collection and interpretation, critical review of the manuscript writing, and discussion of the manuscript. AS worked on data analysis, data interpretation, and writing of the manuscript. MSB worked on data analysis, data interpretation, and writing of the manuscript. HM worked on literature search, data interpretation, and writing of the manuscript. MS worked on data analysis, data interpretation, and writing of the manuscript. JMG worked on data analysis, data interpretation, and writing and revising of the manuscript. HS worked on the project concept and manuscript design, supervising, critically review of the manuscript writing, and discussion the manuscript. All authors read and approved the final manuscript.

Patient or public involvement

No patients or members of the public were involved in the present study.

Acknowledgments
The authors wish to thank the provincial and national public health professionals who contributed to this study, the WHO for their funding support, and the *BMJ Open* editor and peer reviewers for their valuable insights.

References

1. Muñoz-Sastre D, Rodrigo-Martín L, Rodrigo-Martín I. The Role of Twitter in the WHO's Fight against the Infodemic. International Journal of Environmental Research and Public Health. 2021;18(22):11990.
2. Maharlouei N, Hosseinpour P, Erfani A, Shahriarirad R, Raeisi Shahrakie H, Rezaianzadeh A, et al. Factors associated with reluctancy to acquire COVID-19 vaccination: A cross-sectional study in Shiraz, Iran, 2022. Plos one. 2022;17(12):e0278967.

For peer review only - http://bmjopen.bmj.com/site/about/guidelines.xhtml

For peer review only

3. Charkazi A, Allah Kalteh E, Yatimparvar G, Rahimzadeh H, Koochaki G, Shahini N, et al. Prevalence of COVID-19 Vaccination Hesitancy and its Associated Factors based on the Health Belief Model among Iranian People in 2021. Health Education Health Promotion. 2022;10(4):679-85.
4. Mirzaie A, Halaji M, Dehkordi FS, Ranjbar R, Noorbazargan H. A narrative literature review on traditional medicine options for treatment of corona virus disease 2019 (COVID-19). Complementary therapies in clinical practice. 2020;40:101214.
5. Khaniki H, Rasi Tehrani H. Theorizing for Covid 19 infodemic in Iran social media. Quarterly of Social Studies Research in Iran. 2022:933-50.
6. Mheidly N, Fares J. Leveraging media and health communication strategies to overcome the COVID-19 infodemic. Journal of public health policy. 2020;41(4):410-20.
7. The Lancet Infectious D. The COVID-19 infodemic. The Lancet Infectious diseases. 2020;20(8):875-.
8. Health Topics: Infodemic [Internet]. Available from: https://www.who.int/health-topics/infodemic#tab=tab_1.
9. Yang K-C, Pierri F, Hui P-M, Axelrod D, Torres-Lugo C, Bryden J, et al. The covid-19 infodemic: Twitter versus facebook. Big Data & Society. 2021;8(1):20539517211013861.
10. Patel MP, Kute VB, Agarwal SK, Nephrology C-WGoISo. "Infodemic" COVID 19: More Pandemic than the Virus. Indian journal of nephrology. 2020;30(3):188-91.
11. Rathore FA, Farooq FJJPMA. Information overload and infodemic in the COVID-19 pandemic. 2020;70(5):S162-S5.
12. Fernández-Torres MJ, Almansa-Martínez A, Chamizo-Sánchez R. Infodemic and fake news in Spain during the COVID-19 pandemic. International journal of environmental research and public health. 2021;18(4):1781.
13. Sørensen K, Pelikan JM, Röthlin F, Ganahl K, Slonska Z, Doyle G, et al. Health literacy in Europe: comparative results of the European health literacy survey (HLS-EU). European journal of public health. 2015;25(6):1053-8.
14. Lorini C, Ierardi F, Bachini L, Donzellini M, Gemmi F, Bonaccorsi G. The antecedents and consequences of health literacy in an ecological perspective: results from an experimental analysis. International journal of environmental research and public health. 2018;15(4):798.
15. Vinck P, Pham PN, Bindu KK, Bedford J, Nilles EJ. Institutional trust and misinformation in the response to the 2018–19 Ebola outbreak in North Kivu, DR Congo: a population-based survey. The Lancet Infectious Diseases. 2019;19(5):529-36.
16. Carr-Hill R, Currie E. What explains the distribution of doctors and nurses in different countries, and does it matter for health outcomes? Journal of Advanced Nursing. 2013;69(11):2525-37.
17. Zar HJ, Dawa J, Fischer GB, Castro-Rodriguez JA. Challenges of COVID-19 in children in low-and middle-income countries. Paediatric respiratory reviews. 2020;35:70-4.
18. Geldsetzer P. Knowledge and perceptions of COVID-19 among the general public in the United States and the United Kingdom: a cross-sectional online survey. Annals of internal medicine. 2020;173(2):157-60.
19. Islam MS, Sarkar T, Khan SH, Kamal A-HM, Hasan SM, Kabir A, et al. COVID-19–related infodemic and its impact on public health: A global social media analysis. The American journal of tropical medicine and hygiene. 2020;103(4):1621.
20. Kim HK, Ahn J, Atkinson L, Kahlor LA. Effects of COVID-19 misinformation on information seeking, avoidance, and processing: A multicountry comparative study. Science Communication. 2020;42(5):586-615.
21. Lockyer B, Islam S, Rahman A, Dickerson J, Pickett K, Sheldon T, et al. Understanding COVID-19 misinformation and vaccine hesitancy in context: Findings from a qualitative study involving citizens in Bradford, UK. Health Expectations. 2021;24(4):1158-67.
22. Mirhashemi SMH. Infodemic in the countries around the world after corona virus outbreak; Necessity of Media literacy and information literacy improvement for citizens. Applied Politics. 2022;3(1):239-50.

For peer review only - http://bmjopen.bmj.com/site/about/guidelines.xhtml

23. World Health Organization. Infodemic management: an overview of infodemic management during COVID-19, January 2020–May 2021. 2021.
24. Tangcharoensathien V, Calleja N, Nguyen T, Purnat T, D'Agostino M, Garcia-Saiso S, et al. Framework for managing the COVID-19 infodemic: methods and results of an online, crowdsourced WHO technical consultation. Journal of medical Internet research. 2020;22(6):e19659.
25. Lohiniva A-L, Nurzhynska A, Hudi A-h, Anim B. Infodemic Management Using Digital Information and Knowledge Cocreation to Address COVID-19 Vaccine Hesitancy: Case Study From Ghana. JMIR infodemiology. 2022;2(2):e37134.
26. Naeem SB, Bhatti R. The Covid-19 'infodemic': a new front for information professionals. Health Information & Libraries Journal. 2020;37(3):233-9.
27. Eysenbach G. How to fight an infodemic: the four pillars of infodemic management. Journal of medical Internet research. 2020;22(6):e21820.
28. Radu R. <? covid19?> Fighting the 'Infodemic': Legal Responses to COVID-19 Disinformation. Social Media+ Society. 2020;6(3):2056305120948190.
29. Hua J, Shaw R. Corona virus (Covid-19)"infodemic" and emerging issues through a data lens: The case of china. International journal of environmental research and public health. 2020;17(7):2309.
30. Organization WH. Everybody's business: strengthening health systems to improve health outcomes: WHO's framework for action. Geneva: WHO; 2007. 2018.
31. Bradley EH. Intersectoral collaboration: what works and what doesn't. International Journal of Health Policy Management. 2023.
32. Hiller M, Bracht H, Schroeder S. One year with the COVID-19 pandemic– Lessons learnt? Intersectoral collaboration measures established during the crisis could benefit capacity and patient flow management in daily clinical practice. Journal of Health Organization Management. 2022;36(2):141-8.
33. White D. Application of systems thinking to risk management:: a review of the literature. Management Decision. 1995.
34. Olson CM. Consensus statements: applying structure. JAMA. 1995;273(1):72-3.
35. Geerts JM, Kinnair D, Taheri P, Abraham A, Ahn J, Atun R, et al. Guidance for health care leaders during the recovery stage of the COVID-19 pandemic: a consensus statement. JAMA network open. 2021;4(7):e2120295-e.
36. Dossett LA, Kaji AH, Cochran A. SRQR and COREQ reporting guidelines for qualitative studies. JAMA surgery. 2021;156(9):875-6.
37. Braun V, Clarke V. Using thematic analysis in psychology. Qualitative research in psychology. 2006;3(2):77-101.
38. Press TA. WHO chief warns world leaders against "politicizing" pandemic. CBC News. 2020.
39. Ofrin R, Buddha N, Htike M, Bhola A, Bezbaruah S. Strengthening risk communication systems for public health emergencies in the WHO South-East Asia Region. WHO South-East Asia Journal of Public Health. 2020;9(1):15-20.
40. Eldman. Eldman Trust Baromter 2020 2020 [Available from: https://www.edelman.com/sites/g/files/aatuss191/files/2020-03/2020%20Edelman%20Trust%20Barometer%20Coronavirus%20Special%20Report_0.pdf.
41. Brennen JS, Simon FM, Howard PN, Nielsen RK. Types, sources, and claims of COVID-19 misinformation: University of Oxford; 2020.
42. Neavel C, Watkins SC, Chavez M. Youth, social media, and telehealth: How COVID-19 changed our interactions. Pediatric Annals. 2022;51(4):e161-e6.
43. Goulbourne T, Yanovitzky I. The communication infrastructure as a social determinant of health: implications for health Policymaking and practice. The Milbank Quarterly. 2021;99(1):24.
44. Hering JG. Do we need "more research" or better implementation through knowledge brokering? Sustainability Science. 2016;11(2):363-9.
45. World Health Organization. Communicating risk in public health emergencies: a WHO guideline for emergency risk communication (ERC) policy and practice: World Health Organization; 2017.

For peer review only - http://bmjopen.bmj.com/site/about/guidelines.xhtml

46. Khan S, Mishra J, Ahmed N, Onyige CD, Lin KE, Siew R, et al. Risk communication and community engagement during COVID-19. International Journal of Disaster Risk Reduction. 2022;74:102903.
47. Chakrabarti A. Confusion in Italy, clarity in Singapore — how govts are communicating during coronavirus 2020 [cited 2022 26/11]. Available from: https://theprint.in/world/confusion-in-italy-clarity-in-singapore-how-govts-are-communicating-during-coronavirus/378932/.
48. Xie L, Pinto J, Zhong B. Building community resilience on social media to help recover from the COVID-19 pandemic. Computers in Human Behavior. 2022;134:107294.

For peer review only

For peer review only - http://bmjopen.bmj.com/site/about/guidelines.xhtml

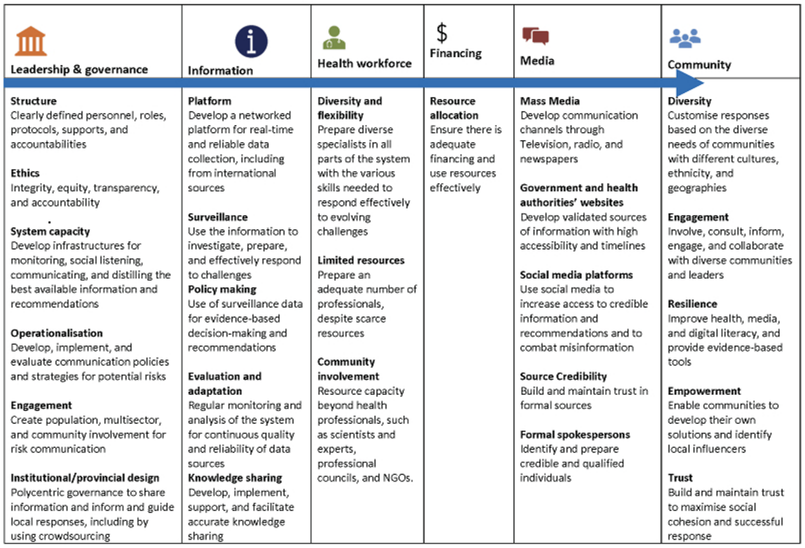

Figure 1

668x471mm (38 x 38 DPI)

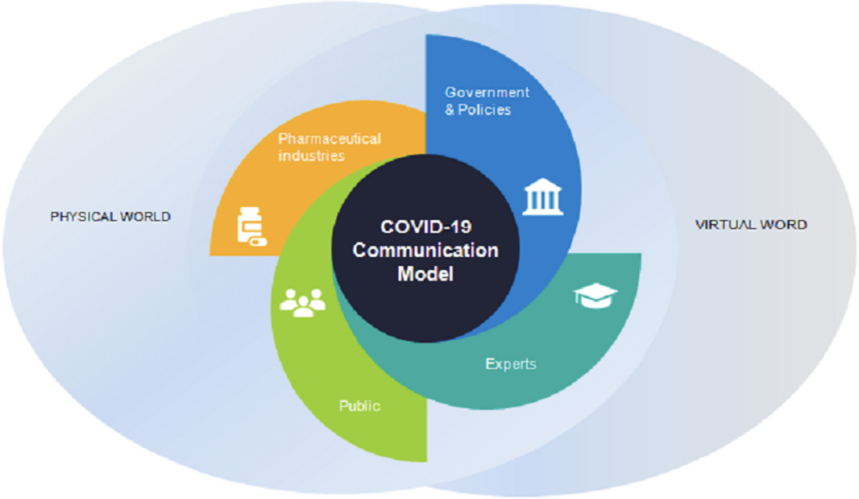

Figure 2

235x136mm (108 x 108 DPI)

Interview guide

Part I: Demographic information

Age ……….

Gender Female / Male

Educational level ……….

Academic discipline ………

What organizational position were you involved in at the time of the Covid 19 epidemic? ………..

Part II: Perceptions, experiences, future directions

The main purpose of the questions in this section is to identify effective strategies and successful experiences in the field of infodemic management related to Covid 19 in Kerman province. Please answer the following questions based on your experiences or field observations.

1- The spread of misleading, inaccurate, and fake information about COVID-19 disease and vaccination has been one of the consequences of the COVID-19 epidemic, which affects the behavior of society and trust in the health system. What experience did you have in managing misinformation? What did you do in a situation in the province where accurate information was not yet available? Can you explain your own experiences in this field?
2- 2. What challenges and obstacles did you face in combating inaccurate information and infodemic management?
3- What did you do in response to the obstacles and challenges?
4- How did you find out about the effectiveness of your interventions and actions?
5- 5- If the pandemic situation is repeated, what is your approach to managing infodemic?

For peer review only

Informed Consent

Hi,

My name is Azam Bazrafshan. My colleague and I are from the Kerman University of Medical Sciences. We interview executives, technical experts, decision-makers, and leaders of public health initiatives who had participated in the provincial, or national COVID-19 prevention and control programs to use the results to improve health interventions during epidemics, pandemics, and global health crises. We are intended to investigate processes, infrastructure, strategies, successful experiences and challenges in the field of infodemic management related to Covid 19 in Kerman province. You are being invited to take part in this research because we feel that your experience as a public health leader can contribute much to our understanding and knowledge of processes and infrastructure of infodemic management during health epidemics.

Your participation in this research is entirely voluntary. It is your choice whether to participate or not. If you choose not to participate all the services you receive at this Centre will continue and nothing will change.

In this interview, I will not ask your name, nor will I need your address. All your answers will be completely confidential. We only use the total responses for statistical survey. During this interview, private questions may also be asked and I have to emphasize that although your honest cooperation is valuable, you can answer any question you think appropriate. The estimated time of interview is about 30 minutes and the interview is recorded by tape recorder.

You do not have to take part in this research if you do not wish to do so, and choosing to participate will not affect your job or job-related evaluations in any way. You may stop participating in the interview at any time that you wish without your job being affected. I will give you an opportunity at the end of the interview to review your remarks, and you can ask to modify or remove portions of those, if you do not agree with my notes or if I did not understand you correctly.

I have read the foregoing information, or it has been read to me. I have had the opportunity to ask questions about it and any questions I have been asked have been answered to my satisfaction. I consent voluntarily to be a participant in this study

Signature of Participant ___________________

Date ___________________________Day/month/year

Signature

For peer review only

Appendix 3. Themes and subthemes related to the challenges and successful activities of infodemic management across provincial/national public health settings in Iran

Theme	Sub-theme	Sample Quotes
**Leadership & governance**		
Ethics	Transparency	When I report falsely on the radio, people would understand. If I have this transparency at the national and university level, people will gain their trust, but this strategy does not exist, it certainly does not exist, neither at the national level, nor at the university or provincial level. (Man, 47 years old)
		There is a level of transparency that the government should have, the officials should have, I would say that it is extremely small considering the structure of our country. (Man, 42 years old)
	Accountability	No one was responsible, a disaster happened, I learned by myself, it was unknown to us, we could have managed this if the ministry had interfered less, the ministry acted badly and this bad behavior spread everywhere.(Man, 55 years old)
	Conflicts of interests	Conflict of interests is one of the most important challenges in the Ministry of Health, which hinders transparency. Profit seeking of companies that produce personal protective equipment, diagnostic and therapeutic equipment, and vaccines, is One of the most important examples of conflicts of interests during the pandemic management. (Man, 70 years old)
Capacity	Infrastructures	We need a quick reaction team that is in contact with academic centers, regularly monitors and examines community's needs and concerns. (Man, 49 years old)<br><br>... and can quickly identify rumours and false information and design an answer to them. (Man, 42 years old)<br><br>A multi-disciplinary risk communication team should be formed to be responsible for informing and making decisions. (Man, 58 years old)
	Rules & regulations	During the pandemic, there were people who published false and contradictory information, and there was no authority to deal with this issue and deal with them judicially, while in many countries, when the issue of people's health is discussed, false information is not allowed to be published. (Man, 70 years old)
	Policies and strategies	There is no specific strategy and program to deal with infodemic and manage risk communication in the country. (Man, 70 years old)
	Role definition	The duties and responsibilities of people in crisis situations should be clear so that everyone does not act and speak as they wish... The goals of the programs should be clear and the responsibilities of each member of the committees should be clearly stated. (Man, 53 years old)
Operationalisation	Timeliness	In order to prepare in crisis conditions, it is necessary to make necessary plans before every crisis. (Man, 45 years old)
	Coordination	One of our most important problems was the lack of coordination between the government and the officials in the matter of information. There were several voices and no coordination between different sources in the health department. (Man, 58 years old)

For peer review only

Institutional/provincial design	Knowledge exchange	In the discussion of pandemic management, the provinces were left to their own devices, and no province was aware of the activities of other provinces in the management of the infodemic. (Man, 55 years old)
**Information**		
**Theme**	**Sub-theme**	**Sample quotes**
Surveillance	Timeliness	No real-time data were available about the mortality of COVID-19 cases. Even now, if we refer to the statistical systems, we will not get a correct and reliable information and coordination so that we can inform the community. (Man, 48 years old)<br><br>Unfortunately, many of the events that happened in the country have not been documented and, for example, we do not know how many patients were infected with Corona, how many died. Even the medical and nursing systems used to come and give general information. It showed that either they don't have this information or they don't want to publish it.(Man, 49 years old)
	Data quality	No consensus data were available as mortality data reported by the hospitals, forensic systems, and cemeteries, were very contradictory. (Man, 48 years old)
	Surveillance systems	Surveillance data were extremely out of date, with low quality and consistency. (Man, 54 years old)<br><br>Our surveillance data were not accurate and real-time. (Man, 48 years old)<br><br>Therefore, it seems that we need a system that collects information from the environmental levels in a database in the form of software that has the power of analysis to give us alarms in different places. Its infrastructure in the country is planned as a syndromic care system, but I don't know if it has actually been implemented. (Man, 49 years old)
Knowledge translation	Evidence-informed policy making	No evidence about the effectiveness of interventions were synthesised and published for decision making. (Man, 54 years old)<br><br>The next problem was that the correct information did not reach those who should manage infodemic, for example, the number of patients at any moment, the number of deaths, what was the cause of death. (Man, 52 years old)
	Knowledge translation Capacity	Most of the statistics and information will be based on taste and subjective and this will cause individual perceptions and people will allow themselves to give any statistics. (Man, 48 years old)
Health workforce		
**Theme**	**Sub-theme**	**Sample Quote**
Capacity building	Diversity and flexibility	That's why we have to find an entry in the educational fields and teach this issue seriously in the form of workshops for groups close to graduation or students in the form of refresher courses regularly and continuously. Let's define a retraining unit for it and implement it operationally, not just theoretically. (Man, 48 years old)
Challenges	Limited resources	One of our most important challenges in risk communication and infodemic management is the lack of trained and expert people in this field. (Man, 70 years old)

For peer review only

Engagement	Community involvement	The non-participation of experts from different scientific fields in the pandemic management and informing the people was a big challenge, which caused people who had good experience or knowledge to be ignored, and therefore, unqualified people were in charge of informing the people. (Man, 42 years old)
**Financial resources**		
**Theme**	**Sub-theme**	**Sample Quote**
Government resources	Planning	Particular financial sources should be allocated to the risk communication activities. (Man, 66 years old)
**Media**		
**Theme**	**Sub-theme**	**Sample Quote**
Communication channels	Mass media	National TV and Radio channels are still the biggest and most influential communication channels in Iran. (Man, 46 years old)
	Government & health authorities' websites	We used both video media such as radio and television, as well as written media such as magazines, newspapers, and government websites, which were very active during the Corona era, to communicate the data related to the incidence of the disease in the province and recommend preventive measures. (Man, 49 years old)
	Social media platforms	We have established a social media platform (Instagram) named Dr+ to communicate with people and held online discussion panels with contribution of clinical physicians to address the community's needs and concerns. (Man, 53 years old)
Trust	Source credibility	Communication channels should be used that are highly credible and people trust to them. Some brand communication channels should be developed to maximize the impact and penetration of information among people. (Man, 47 years old)
spokespersons	Competence	For a person to be a spokesperson and to give information, to know how to give information, not to be too hopeful, not to speak too hopelessly, this is real information when we say not only to report numbers... For example, when the pandemic came, someone said that there is nothing, someone said Wow, we are unfortunate, which one of these people should accept when they look at it? (Man, 58 years old)
**Community**		
**Theme**	**Sub-theme**	**Example Strategies**
Diversity	Social context	The penetration rate of scientific issues in our society is low, which is related to various issues, so if we ever want to increase this penetration rate, we have to approach from different social and cultural aspects. (Man, 54 years old)
Engagement & empowerment	interventions	We have developed a community engagement facility to listen the community's needs and expectations and answer to their concerns and questions interactively. (Man, 55 years old)<br><br>Safiran-e-Salamat was a group of trained volunteers who communicate health information with their families and their neighbourhood. (Man, 55 years old)

For peer review only

Trust	Trust to government and health officials	Public opinion has no trust in the government, especially in matters that are officially announced. (Man, 47 years old)<br><br>Anyone, any scientist, any distinguished person, any accepted person comes and says something, the first time people do not accept it, especially if it is actually what the government says or emphasizes, people will definitely look for the opposite and say that there is something fishy about it. (Man, 53 years old)

For peer review only

Standards for reporting qualitative research (SRQR) checklist

No	Topic	Item	Page number
		Title and abstract	
S1	Title	Concise description of the nature and topic of the study Identifying the study as qualitative or indicating the approach (e.g., ethnography, grounded theory) or data collection methods (e.g., interview, focus group) is recommended	1
S2	Abstract	Summary of key elements of the study using the abstract format of the intended publication; typically includes background, purpose, methods, results, and conclusions	1
		Introduction	
S3	Problem formulation	Description and significance of the problem/phenomenon studied; review of relevant theory and empirical work; problem statement	2
S4	Purpose or research question	Purpose of the study and specific objectives or questions	3
		Methods	
S5	Qualitative approach and research paradigm	Qualitative approach (e.g., ethnography, grounded theory, case study, phenomenology, narrative research) and guiding theory if appropriate; identifying the research paradigm (e.g., postpositivist, constructivist/ interpretivist) is also recommended; rationale	4
S6	Researcher characteristics and reflexivity	Researchers' characteristics that may influence the research, including personal attributes, qualifications/experience, relationship with participants, assumptions, and/or presuppositions; potential or actual interaction between researchers' characteristics and the research questions, approach, methods, results, and/or transferability	-
S7	Context	Setting/site and salient contextual factors; rationale	4
S8	Sampling strategy	How and why research participants, documents, or events were selected; criteria for deciding when no further sampling was necessary (e.g., sampling saturation); rationale	4

For peer review only

S9	Ethical issues pertaining to human subjects	Documentation of approval by an appropriate ethics review board and participant consent, or explanation for lack thereof; other confidentiality and data security issues	4
S10	Data collection methods	Types of data collected; details of data collection procedures including (as appropriate) start and stop dates of data collection and analysis, iterative process, triangulation of sources/methods, and modification of procedures in response to evolving study findings; rationale	4-5
S11	Data collection instruments and technologies	Description of instruments (e.g., interview guides, questionnaires) and devices (e.g., audio recorders) used for data collection; if/how the instrument(s) changed over the course of the study	4-5
S12	Units of study	Number and relevant characteristics of participants, documents, or events included in the study; level of participation (could be reported in results)	NA
S13	Data processing	Methods for processing data prior to and during analysis, including transcription, data entry, data management and security, verification of data integrity, data coding, and anonymization/deidentification of excerpts	4-5
S14	Data analysis	Process by which inferences, themes, etc., were identified and developed, including the researchers involved in data analysis; usually references a specific paradigm or approach; rationale	4-5
S15	Techniques to enhance trustworthiness	Techniques to enhance trustworthiness and credibility of data analysis (e.g., member checking, audit trail, triangulation); rationale	5
Results/findings			
S16	Synthesis and interpretation	Main findings (e.g., interpretations, inferences, and themes); might include development of a theory or model, or integration with prior research or theory	5
S17	Links to empirical data	Evidence (e.g., quotes, field notes, text excerpts, photographs) to substantiate analytic findings	6-appendix 3
Discussion			
S18	Integration with prior work, implications, transferability, and contribution(s) to the field	Short summary of main findings; explanation of how findings and conclusions connect to, support, elaborate on, or challenge conclusions of earlier scholarship; discussion of scope of application/ generalizability; identification of unique contribution(s) to scholarship in a discipline or field	11
S19	Limitations	Trustworthiness and limitations of findings	13
Other			

For peer review only

S20	Conflicts of interest	Potential sources of influence or perceived influence on study conduct and conclusions; how these were managed	14
S21	Funding	Sources of funding and other support; role of funders in data collection, interpretation, and reporting	15

For peer review only

BMJ Open

Health risk communication and infodemic management in Iran: Development and validation of a conceptual framework

Journal:	*BMJ Open*
Manuscript ID	bmjopen-2023-072326.R2
Article Type:	Original research
Date Submitted by the Author:	01-Jul-2023
Complete List of Authors:	Bazrafshan, Azam; Kerman University of Medical Sciences, HIV/STI Surveillance Research Center, and WHO Collaborative Center for HIV Surveillance, Institute for Futures Studies in Health,<br>Sadeghi, Azadeh; Kerman University of Medical Sciences, HIV/STI Surveillance Research Center, and WHO Collaborative Center for HIV Surveillance, Institute for Futures Studies in Health,; Kerman University of Medical Sciences, Deputy of Health, Department of Communicable Diseases<br>Bazrafshan, Maliheh Sadat; Kerman University of Medical Sciences, HIV/STI Surveillance Research Center, and WHO Collaborative Center for HIV Surveillance, Institute for Futures Studies in Health,<br>Mirzaie, Hossein; Kerman University of Medical Sciences, HIV/STI Surveillance Research Center, and WHO Collaborative Center for HIV Surveillance, Institute for Futures Studies in Health,<br>Shafiee, Mehdi; Kerman University of Medical Sciences, HIV/STI Surveillance Research Center, and WHO Collaborative Center for HIV Surveillance, Institute for Futures Studies in Health,; Kerman University of Medical Sciences, Deputy of Health, Department of Communicable Diseases<br>Geerts, Jaason; Canadian College of Health Leaders; City University of London, Bayes Business School<br>Sharifi, Hamid; Kerman University of Medical Sciences, HIV/STI Surveillance Research Center, and WHO Collaborating Center for HIV Surveillance, Institute for Futures Studies in Health,
**Primary Subject Heading**:	Public health
Secondary Subject Heading:	Health policy
Keywords:	COVID-19, Health policy < HEALTH SERVICES ADMINISTRATION & MANAGEMENT, INFECTIOUS DISEASES

SCHOLARONE™
Manuscripts

I, the Submitting Author has the right to grant and does grant on behalf of all authors of the Work (as defined in the below author licence), an exclusive licence and/or a non-exclusive licence for contributions from authors who are: i) UK Crown employees; ii) where BMJ has agreed a CC-BY licence shall apply, and/or iii) in accordance with the terms applicable for US Federal Government officers or employees acting as part of their official duties; on a worldwide, perpetual, irrevocable, royalty-free basis to BMJ Publishing Group Ltd ("BMJ") its licensees and where the relevant Journal is co-owned by BMJ to the co-owners of the Journal, to publish the Work in this journal and any other BMJ products and to exploit all rights, as set out in our licence.

The Submitting Author accepts and understands that any supply made under these terms is made by BMJ to the Submitting Author unless you are acting as an employee on behalf of your employer or a postgraduate student of an affiliated institution which is paying any applicable article publishing charge ("APC") for Open Access articles. Where the Submitting Author wishes to make the Work available on an Open Access basis (and intends to pay the relevant APC), the terms of reuse of such Open Access shall be governed by a Creative Commons licence – details of these licences and which Creative Commons licence will apply to this Work are set out in our licence referred to above.

Other than as permitted in any relevant BMJ Author's Self Archiving Policies, I confirm this Work has not been accepted for publication elsewhere, is not being considered for publication elsewhere and does not duplicate material already published. I confirm all authors consent to publication of this Work and authorise the granting of this licence.

For peer review only

Health risk communication and infodemic management in Iran: development and validation of a conceptual framework

Authors: Azam Bazrafshan[1], Azadeh Sadeghi[1,2], Maliheh Sadat Bazrafshan[1], Hossein Mirzaie[1], Mehdi Shafiee[1,2], Jaason M. Geerts[3,4], Hamid Sharifi[1*]

1 HIV/STI Surveillance Research Center, and WHO Collaborative Center for HIV Surveillance, Kerman University of Medical Sciences

2 Department of Communicable Diseases, Deputy of Health, Kerman University of Medical Sciences, Kerman, Iran

3 Honorary Visiting Fellow, Bayes Business School, University of London, UK.

4 Director of Research and Leadership Development, The Canadian College of Health Leaders, Ottawa, Canada.

*Corresponding author: Hamid Sharifi (Sharifihami@gmail.com)

Abstract

Objective: the COVID-19 pandemic exposed significant gaps in Iran's and other health systems' risk communication. The accompanying infodemic undermined policy responses, amplified distrust in government, and reduced adherence to public health recommendations among the Iranian population. This study aimed to develop a conceptual framework for health risk communication and infodemic management (RCIM) during epidemics and health emergencies in Iran that could have potential applications in other contexts.
Design: this study was designed in two phases. Phase 1 involved semi-structured qualitative interviews with key informants to explore effective RCIM strategies across public health settings in Iran and to develop a conceptual framework. Phase 2 involved revising the framework based on feedback from an online expert panel regarding its comprehensiveness and validity.
Setting: provincial/national public health settings in Iran.
Participants: twenty key informants from provincial and national public health authorities who contributed to COVID-19 response programs participated in interviews. Nine experts from diverse academic disciplines, provincial and national settings, and geographical locations participated in an online expert panel.
Results: the conceptual model was created based on qualitative interviews and expert panel discussions and was structured according to four pillars of the World Health Organization (WHO) health system framework: leadership and governance, information, health workforce, and financial resources, along with media and community. Leadership and governance, including trustworthy leaders, were recommended as the foundation for developing RCIM in Iran. Developing an official strategy with information infrastructures, including high-quality surveillance systems, identified personnel and training for specialists among the health workforce, financial resources, communication channels, and community engagement, were recognised as other dimensions for developing health risk communication in Iran.
Conclusion: the proposed framework represents a step toward establishing a national health risk communication strategy in Iran. Further validation of the conceptual framework and experiments on how it could potentially influence policy and practice are recommended. This model has the potential to be applied in other contexts in its current form or as the foundation for customised local versions.

For peer review only - http://bmjopen.bmj.com/site/about/guidelines.xhtml

Keywords
risk communication, leadership, infodemic, COVID-19, emergency preparedness, crisis response

Strengths and limitations of this study

- This study consolidates insights from the field experiences of public health professionals across provincial and national settings in risk communication and infodemic management (RCIM) during the COVID-19 pandemic in Iran. Investigating the experiences and perceptions of academics, health professionals, and policy-makers enhances the validity of the results by including diverse perspectives on the topic of RCIM, and strengthens the proposed framework's credibility by providing a comprehensive understanding of its applicability in provincial and national public health settings.
- This study presents a novel conceptual framework, validated through full consensus by a panel of experts, for risk communication and infodemic management during epidemics and health emergencies in Iran.
- The qualitative nature of our study and the focus on one country may limit the perceived validity, however, involving two phases of diverse experts increases the potential relevance of the framework to other contexts

Introduction

The COVID-19 pandemic has changed our world, having affected every sector significantly, including health, education, economic, social, cultural, and informational. One of the social repercussions of the pandemic has been the constant spread through various media of overwhelming volumes of information, particularly concerning health, public health, government directives, and related issues. Much of this has been "misinformation" and "disinformation", both of which refer to incorrect or misleading content, the difference being the intentionality of those engaging in disinformation to cause harm, whereas misinformation is non-malicious but still potentially dangerous. Sources of mis- and disinformation range from non-stringently reviewed rapid academic publications with non-credible or flawed methodologies - and thereby dubious conclusions (misinformation), to "fake news" through anonymous social media posts and intentionally misleading messaging by government officials (disinformation) (1).

False information, combining accidental and intentional, has contributed significantly to misguided health policies and to a host of deleterious consequences for individual and population health (2, 3). This phenomenon is called an "infodemic". The World Health Organization (WHO) defines an infodemic as, "the widespread distribution of false or misleading information in digital and physical environments during a disease outbreak" (4). Without robust systemic safeguards in place, an infodemic can make communities, jurisdictions, and whole populations more vulnerable to disease infection and their side effects, as well as to other related harms (5). Information overload, including the infodemic, during the COVID-19 pandemic, has represented a parallel pandemic whose transmission rate is much faster than the disease itself, since rampant erroneous and prejudicial information can trigger the spread of wild and accelerated waves of fear and defiance in the general population (6). In Iran, for example, there is evidence, though limited, that the infodemic spread widely through social media during the pandemic was associated with significant COVID-19 vaccine hesitancy rates (7, 8), substantial uptake of traditional and complementary medicine products (9), and poor adherence to preventive measures, such as masking, in the general population (10). This escalation reinforces the importance of infodemic management in Iran.

Infodemics can severely change a pandemic's course by undermining public health and government recommendations and by diminishing population and community adherence to public health interventions such as, masking, social distancing, and vaccination.. Economically disadvantaged countries are at higher risk of infodemics than developed countries, due to a range of inequalities (11). Lower rates of health literacy, limited

For peer review only - http://bmjopen.bmj.com/site/about/guidelines.xhtml

access to reliable health information, and minimal public trust in public health authorities (11) can make people from underdeveloped and developing countries more susceptible to fake news and misinformation (12, 13). This vulnerability is compounded by further inequalities in terms of comparatively limited healthcare infrastructure and reduced access to healthcare facilities and public health professionals, which make people from these countries more prone to sporadic and ill-advised health and public health behaviours (14). In this context, the infodemic can pose a greater threat to populations in underdeveloped and developing countries during epidemics and health emergencies (15) by negatively influencing public risk perceptions and by undermining evidence-based policy creation and national and regional emergency responses (16-18). These hindrances can increase the spread and burden of the pandemic and widen global health disparities.

Infodemics have become a global phenomenon, impacting citizens in every country (19, 20). Addressing them is a new challenge and priority in managing and responding to epidemics and health emergencies. To understand and counter the rapidly changing nature of the COVID-19 infodemic and to mitigate its negative effects, such as the further spread of misinformation, several novel strategies and initiatives have been established across public health settings globally. The WHO has been widely respected for developing highly credible guidelines and initiatives to combat misinformation and infodemic management across the world (21). From early in the COVID-19 response, the WHO began to develop international strategies for infodemic management, in cooperation with other organisations, including the US Center for Disease Control and Prevention (CDC) and the Africa Centre for Disease Control and Prevention. To track and address misinformation surrounding COVID-19 and HIV, UNAIDS and the Africa CDC have been operating a rumour management system—software that uses machine learning, combined with human expertise, to collect and analyse rumour data from open-source traditional media (web-based, news broadcasts), as well as social media (Facebook, Twitter, WhatsApp). The system enables the identification of false and misleading information related to COVID-19 and HIV (21). In addition, the WHO developed a framework for infodemic management through crowd-sourcing and online consultation with a wide range of global public health professionals (22). Multiple countries like Ghana have taken steps to identify, analyse, and respond to COVID-19 and vaccine-related misinformation(23). These initiatives are helpful foundations for further infodemic management strategies.

Risk communication and infodemic management (RCIM) are the core of risk management and effective responses to epidemics and health emergencies (24). According to Eysenbach (2020), there are four pillars of infodemic management: information monitoring, building health and e-health literacy in the general population, consolidating and disseminating credible information, including by accelerating the academic peer-review process, to ensure accurate and timely knowledge translation, and minimising factors, such as political or commercial agendas, that can distort or distract from evidence-based guidance or strategies (25). Combating mis- or disinformation for populations is as critical as ensuring much-needed medical equipment and supplies for health workers are readily available (26). In underdeveloped and developing countries, given their existing health information inequalities and public health vulnerabilities, customized RCIM approaches are needed to combat infodemics and to reduce their effects on population health (27). In particular, engagement and collaboration with local communities and leaders and stricter public health regulations are necessary (27). While some contexts may be more susceptible to the dangerous potential impacts of mis- and disinformation, none is immune, and the consequences of failing to tackle it directly and strategically can be dire.

The purpose of this study was to build on and extend previous conceptualisations of RCIM capacity building by creating a conceptual framework of RCIM in Iran. To achieve this, we applied a systems thinking lens, since the pandemic demonstrated that not only can health emergencies affect all people and sectors, but that addressing

For peer review only - http://bmjopen.bmj.com/site/about/guidelines.xhtml

infodemics requires more than just public health messaging. Along with potential benefits for other sectors, robust national and regional RCIMs can have a significant positive impact on health systems, those who bear the brunt of health emergencies. The WHO describes a health system as a set of interconnected building blocks that are essential to health system functioning. The blocks are: service delivery, health workforce, health information systems, access to essential medicines, financing, and leadership/governance, with the latter being central to all. It is essential that each of these interconnected elements are addressed concomitantly in response to changing population health needs and inequalities, and to epidemics and health emergencies (28). This multifaceted understanding of health systems, along with considerations for other related sectors, is vital to effective RCIM strategies, since mis- and disinformation can affect those in all aspects of society. The nature of health emergencies requires that policy and communications strategy recommendations should be gathered from a diverse group of actors with relevant RCIM expertise, including researchers, educators, advocates, practitioners, funders, private sector representatives, community representatives, government officials, policymakers, and various trusted international experts and representatives. Leaders from across sectors should also collaborate with public health and with each other to integrate RCIM strategies effectively to improve the health of all people and communities (29, 30). Applying these diverse perspectives and the systems thinking approach can enhance RCIM policies, strategies, and activities nationally, regionally, and locally and can lead to improved relevant health outcomes during epidemics and health emergencies (31).

Methods

This sequential, mixed-methods exploratory study was conducted in two phases from October to December 2022. Phase 1 involved semi-structured interviews with key informants from provincial and national public health authorities to inform the creation of an initial framework of key RCIM components across settings. Phase 2 involved an online panel of experts from relevant scientific domains to validate the conceptual framework's validity, credibility, and transformability (32, 33). We then revised the framework based on the panel's feedback (Figure 1). This study followed the Standards for Reporting Qualitative Research (SRQR) checklist (34).

Phase 1: semi-structured interviews

Phase 1 involved semi-structured interviews with a purposive sample of 20 Iranian public health professionals across provincial and national health authorities. Study participants included stakeholders, academics, decision-makers, and leaders with expertise in community health, epidemiology, public health, social medicine, health communication, and sociology. Participants were from eight pre-specified provinces: Kerman, Tehran, Fars, Isfahan, Mazandaran, West-Azerbaijan, Kermanshah, and Sistan va Baloochestan. These provinces were initially selected to involve a representative sample of the Iranian population with diverse social, geographical, and cultural characteristics. Inclusion criteria were: 1) having at least one year of experience in either COVID-19 prevention and control programs or decision-making in provincial or national public health settings, and 2) willingness to participate in the study.

An interview guide was developed according to previous studies (Appendix 1). The interview guide focused on the processes, infrastructures, challenges encountered, and best practices relevant to RCIM during the COVID-19 pandemic in Iran. The interview guide was assessed beforehand by two expert reviewers. It was subsequently pre-tested with three target population members before the implementation.

The interviews followed a semi-structured design, allowing for variations of the order of the questions and follow-up questions based on participant responses. The objectives and the activities that were involved in the study were explained to the participants. The principal investigator's contact details were provided, and participants'

For peer review only - http://bmjopen.bmj.com/site/about/guidelines.xhtml

confidentiality was guaranteed. Written consent was sought before the interview, and the participants were asked to email the completed form to the principal investigator (Appendix 2). An experienced interviewer with a background in qualitative research and interviewing expertise conducted the interviews in the Farsi language. Due to COVID-19 social distancing, all interviews were conducted by telephone, audio-recorded, and transcribed verbatim. Interviews ranged between 20–55 minutes (mean = 34 minutes). Interviews lasted until the researchers realised they had reached content saturation.

To analyse the interview data, all interviews were transcribed verbatim. Then one of co-authors extracted concepts and open codes using Braun and Clarke's framework for thematic analysis of qualitative data (35) to the interview transcripts. The authors define thematic analysis as, "the process of identifying patterns or themes within qualitative data" (p. 78). Their framework involves six steps: becoming familiar with the data, generating initial codes, searching for themes, reviewing themes, defining themes, and writing up.
The initial set of open codes, themes, and sub-themes was discussed by participants and subsequently reviewed by the entire research team to improve the credibility and trustworthiness of the qualitative study. We used MAXQDA 12 (VERBI GmbH, USA) for manual coding and content analysis.

Based on the themes identified from the qualitative interviews and subsequent inspections, we created an initial set of 33 key RCIM strategies and organized them according to four of the pillars of the WHO model of the health systems, along with media and community (28). This initial set of components served as the basis for discussion with, and validation by, the expert panel in Phase 2 and consequently, the conceptual model.

Phase 2: expert panel validation

Phase 2 involved a group of nine experts selected through purposive sampling to validate and prioritise key components of the initial RCIM model and to evaluate its completeness and validity (32, 33). The panel included a diverse set of stakeholders, academics, decision-makers, leaders from the various communities, and national public health leaders. The inclusion criteria for this phase were: 1) having at least three years of professional experience or established research expertise in the fields of public health, epidemiology, crisis management, infodemiology, social media studies, or health communication; and 2) willingness to participate in the study. Potential panel members (n = 9) were identified through their academic or professional roles in health risk communication or risk management activities across provincial or national health authorities during the COVID-19 pandemic. Prospective contributors were given a short statement of the study's purpose and design and were invited by email to participate in the panel discussion. During the discussion, panellists engaged based on their assessments of the initial conceptual model and suggested additions, deletions, and modifications, with the aim of informing a highly complete and credible model of essential components of an RCIM model for the country. As mentioned previously, this validation by experts was also intended to augment the quality, reliability, and validity of the model (32, 33).

Following this phase, several revisions were made to the original conceptual model, but no factor was deemed required for exclusion. The required level of consensus for each component in this phase was a minimum of 75% agreement.

Patient and Public Involvement Statement

No patients or community members were involved in this study.

For peer review only - http://bmjopen.bmj.com/site/about/guidelines.xhtml

Results

Phase 1: semi-structured interviews

Participants

Most participants were men (n=19, 95%), aged 51-60 years old (n=11, 55%), from medical and public health disciplines (n=17, 85%), who work as a provincial or national health officer (n=14, 70%). Participants were mostly from Tehran (n=7, 35%) and Kerman provinces (n=5, 25%) (Table 1).

Table 1: Demographic characteristics of the participants in the interviews (Phase 1)

Demographic characteristics	Frequency (%)
Residence at the time of interviews	
Tehran	7(35)
Kerman	5 (25)
Fars	2 (10)
Isfahan	2 (10)
Kermanshah	1 (5)
Mazandaran	1 (5)
Sistan va Baluchestan	1 (5)
West Azerbaijan	1 (5)
Age	
40-49	6 (30)
50-59	11 (55)
60≥	3 (15)
Gender	
Men	19 (95)
Women	1 (5)
Academic Discipline	
Epidemiology	6 (30)
General medicine	4 (20)
Sociology	3 (15)
Health policy	2 (10)
Infectious disease	2 (10)
Social medicine	2 (10)
Health education & promotion	1 (5)

The analysis of the qualitative data collected during the key informant interviews revealed 948 open codes and 84 sub-themes. Sub-themes were subsequently classified into 33 components (Appendix 3).

The next step involved organising these components according to six categories representing a combination of the WHO model (24) and key aspects of the Iranian health system: leadership and governance, information, health workforce, financial resources, media, and community. The results formed the initial RCIM conceptual model.

For peer review only - http://bmjopen.bmj.com/site/about/guidelines.xhtml

Theme 1: Leadership and Governance

Leadership and governance are at the heart of the WHO model of health systems (28) and Dr. Tedros Ghebreyesus, Director-General of the WHO, said in the early months of the pandemic, “The greatest threat we face now is not the virus itself, it's the lack of global solidarity and global leadership" (36). Similarly, in an international study of crisis leadership featuring 32 co-authors from 17 countries, Geerts et al. (2021) highlighted that effective leadership, trust in leaders through transparent decision-making, communication, and accountability are vital to successful public health strategies (32, 33).

These examples reinforce the finding in our study that every respondent mentioned leadership and governance as essential foundations for the RCIM model. Seven respondents emphasised transparency in decision-making, effective communication, and accountability as important characteristics of effective leadership and governance. According to these respondents, a lack of transparency and accountability among Iranian health officials and government authorities were among the country's substantial weaknesses in risk communication and had adverse consequences. Thirteen respondents suggested that senior public health officials intentionally caused non-transparent information communication during the COVID-19 pandemic, motivated by financial and other competing interests, which, they suggested, eroded public trust significantly. Similarly, regarding sources of false messaging, seven respondents indicated that pharmaceutical companies were a major source of spreading misinformation during the pandemic. These respondents suggested that public health officials to allow their financial and competing interests, including those related to pharmaceutical companies, to deter them from spreading credible information about the efficacy of some new and underdeveloped medications and vaccines. One respondent expanded a perception that many health officials were among shareholders of the pharmaceutical industry, they advertised some drugs or public health products and subsequently caused a fake and unrealistic demand among the population.

Almost all respondents (n =18) emphasised that the health system needs a robust risk communication strategy and increased infodemic management capacity by developing infrastructures for monitoring the public’s risk perception, knowledge and attitudes, communicating with the public, and providing clear guidance through various media based on the best available science. Increased RCIM capacity would enable early detection of outbreaks of potentially harmful mis- and disinformation, and quick responses to counter falsehoods with facts or other reliable information in a targeted way for each audience. One respondent suggested that building capacity should involve designing an infodemic management system that defines national and provincial responsibilities based on lessons learned from credible global guidelines, national and regional successful strategies, challenges, and failures, as well as leading practices, locally and elsewhere. This respondent added that the system should include a national independent core rapid response team with clear roles, protocols, and accountabilities to collaborate with communities to screen and identify their needs, concerns, and misinformation sources, to lead quick responses the potential risks, and to prevent or mitigate the viral spread of mis- and disinformation across the communities. Similarly, six respondents proposed developing, implementing, evaluating, and revising communication policies and strategies to confront potential risks. According to these respondents, the lack of national and provincial policies and programs for RCIM severely inhibited the national COVID-19 control and management efforts. Four respondents argued that the government’s poor management of the COVID-19 infodemic, poor communication with the public and other stakeholders, and a lack of national and provincial strategies to address misinformation were major shortcomings of risk communication and infodemic management in Iran.

For peer review only - http://bmjopen.bmj.com/site/about/guidelines.xhtml

To optimize RCIM strategies, it is crucial to involve representatives from multiple sectors and the community representatives. Eleven respondents indicated that top-down public health initiatives that lacked community-based customisation and approaches were among the major barriers to acceptance of COVID-19 prevention and control interventions during the pandemic. Multi-sector and community involvement could also potentially improve community members' motivation to participate actively in information communication and management of infodemics. For example, one respondent described how social influencers in community-based approaches, such as that in Safiran-e-Salamat, Tehran, served as facilitators for effective risk communication and infodemic management across provincial settings.

Ineffective use of institutional and provincial infrastructures and capacities and lack of crowdsourcing were cited by four participants as major barriers to effective infodemic management during the COVID-19 pandemic. These respondents elaborated that medical universities and faculty within the provinces were isolated from the national health authorities and not supported by the Ministry of Health in planning and decision-making. These two respondents recommended establishing official networks of experts in diverse areas and practitioners to share experiences, challenges, and best practices of information communication during the potential risks and increase capacity.

Along with trustworthy public health guidance and recommendations, two respondents stated that providing all people with tools for filtering, assessing, and fact-checking information is essential to combat misinformation during the pandemic and health emergencies. Five respondents believed that using a well-known and reliable communication channel and technology-based interventions would maximise the spread of valid information and impact communication efforts and strategies.

Theme 2: Information

Developing a network platform to systematically collect, analyse, and interpret epidemiologic data from the community and quickly disseminate the key findings was considered an important characteristic of risk communication by fourteen respondents. These respondents emphasised that a lack of access to real-time, valid, and high-quality data about the incidence, mortality, and burden of the COVID-19 disease in different provinces intensified the potential risk and spread of misinformation among the population.

Similarly, seven respondents indicated that a lack of access to high-quality surveillance data for research activities and to inform responses to potential and emergent challenges reduced the reliability of information and recommendations and transparency of government decisions. Consequently, it raised dramatic social concerns about the government's ability to estimate the spread of the disease and to anticipate and evaluate the effect of specific policies on population health.

In addition to data quality issues, two respondents suggested that the lack of substantial resources to handle the multiplication of data sources and information producers, to monitor disease trends regularly, and to appraise the quality of data sources were major barriers to the effective use of surveillance data for decision-making during the pandemic.

One respondent stated that some politicians, health officials, and media misinterpreted and selectively reported data according to their own financial, commercial, and political interests, which he considered a major source of misinformation during the pandemic. Two respondents argued that effective knowledge translation of high-quality data is required to minimise the spread of misinformation across different sectors and communities, since people's political, commercial, and financial interests can lead them to distort scientific messages.

For peer review only - http://bmjopen.bmj.com/site/about/guidelines.xhtml

Finally, three respondents reported that these data issues contributed to a lack of evidence-based policies and practices, which severely inhibited effective risk communication and infodemic management.

Theme 3: Health workforce

All respondents highlighted the need for well-trained specialists in various organisations with a mix of skills that can contribute to risk communication and infodemic management activities, as well as additional training for all health workers.

Six respondents promoted the benefits of involving public health agencies, epidemiologists, data scientists, and sociologists who have unique expertise and credibility to guide policies, strategies, and risk communication and infodemic management, in collaboration with health workers. However, all respondents agreed that the Iranian scientists and experts have not helped substantially to prevent misinformation and to mitigate the effects of the infodemic. Further, three respondents suggested that, in some cases, scientists and academic experts in infodemic management were considered sources of misinformation, seen as contributing to the infodemic trends by publishing low-quality scientific papers and providing non-credible, sensational, or exaggerated information about new treatments.

To gather relevant data and to disseminate evidence-based guidance, twelve respondents highlighted the need to involve professional councils, NGOs, interested experts, and health volunteers as additional workforce sources to improve the speed and effectiveness of the response to the infodemic.

Three respondents identified a key gap in RCIM: a lack of qualified and well-trained spokespersons in public health and health organisations, which, they suggest, greatly diminished the quality of risk communication activities during the pandemic. Four respondents referenced a range of competencies necessary to improve the quality of the health workforce education and practice, which can be used to select potential candidates for RCIM roles and to design educational courses and curricula to enhance their ability to support health emergency response effectively.

Theme 4: Financing

Three respondents highlighted the importance of effective financial resource allocation to risk communication and infodemic management to support data collection and analysis and communication strategies. One respondent argued that multi-sector collaborations could reduce the risk of underfunding communication responses. Using technology-based interventions, such as text-messaging approaches, could improve the cost-effectiveness of communication strategies.

Theme 5: Media

All respondents mentioned characteristics related to media. Two respondents believed that given the broad coverage and penetration of radio and television (TV) as dominant communication channels in most parts of the country, involvement of trustworthy spokespersons in, and collaboration with, mass media, could improve the effectiveness of risk communication strategies. However, the respondents elaborated that the weak contribution of these media in RCIM was an obstacle to preventing misinformation. Even worse, nine respondents argued that TV and other mass media actually contributed to the COVID-19 infodemic. According to these respondents, broadcasting news reports that included misleading and low-value information, interviews with non-experts, and flagrant criticisms or debates about the performance of public health agencies reduced public trust and prompted many people and communities to rely more on informal and social media channels.

For peer review only - http://bmjopen.bmj.com/site/about/guidelines.xhtml

For peer review only

Three respondents added the need to improve government and health authorities' websites to disseminate real-time and high-quality information, since many consider them the source of credible information. Additionally, six respondents advocated social media platforms as important communication channels for most communities to aid the acceptance of public health interventions. Three respondents elaborated that reduced public trust in formal and government communication channels caused many people to rely instead on social media platforms, viewing them as more trustworthy. For example, according to two respondents, the dissemination of valid and high-quality data through social media channels influenced the impact of local interventions and improved vaccination coverage for vulnerable and ethnic populations, particularly in Sistan-va-Baluchestan and West Azerbaijan. These respondents explained that, due to higher accessibility, social media platforms were highly utilised by younger adults and geographically distanced locations and, therefore, effective in improving the speed and effectiveness of interventions among members of these populations.

Theme 6: Community

Eight respondents reinforced the importance of involving the community in risk communication and infodemic management in two ways. First, by understanding their diverse demographic, social, economic, and cultural compositions and by identifying their information needs, preferred media, and key influencers. Second, by listening to their concerns, sharing key data and evidence-based recommendations with them, and incorporating their input transparently into important, relevant decisions. However, four respondents suggested that the lack of community-centred approaches reduced the effectiveness of risk communication efforts during the COVID-19 pandemic in most Iranian provincial settings. Three respondents recommended priority training in critical thinking, media, and health literacy for community leaders in risk communication and infodemic management to improve their engagement, active contribution, and effectiveness. According to these respondents, well-informed, engaged, and enabled communities can minimise misinformation and infodemic consequences and develop their own local solutions. One respondent expressed that this kind of respectful, reciprocal relationship with communities could rebuild and maintain public trust in public health agencies, health professionals, and government authorities and could also maximise social cohesion and local capacity successfully respond to potential risks during the crisis.

Phase 2: expert panel validation

In this phase, the completeness and trustworthiness of the proposed conceptual model of RCIM in Iran was discussed by the online expert panel until consensus was achieved by all panel members (100% agreement) (Figure 1).

Figure 1: a conceptual model of components and infrastructures of health risk communication and infodemic management system in Iran

Discussion

This study, conducted during the COVID-19 pandemic, was inspired by an awareness of two aspects of the global experience. The first is the extent to which infodemics can influence the course of large-scale health emergencies, given the global impact that the COVID-19 infodemic has had on individual and population health (2, 3). The term "infodemic" refers to the profusion of recurring waves of information of overwhelming volume and predominantly unclear and/or mixed credibility, including disinformation, messaging intended to deceive. Infodemics can erode the quality and effectiveness of policy and strategy decisions. They can also intensify community and population-level distrust in government and public health officials and experts, including their recommendations, which can drastically undermine national

For peer review only - http://bmjopen.bmj.com/site/about/guidelines.xhtml

and local efforts to effectively mitigate the spread of the disease. As people's faith in official sources diminishes, the likelihood of them being influenced by alternatives increases, and the escalation of rumours and fear exacerbates. Broadcasts of incorrect information through TV, radio, newspapers, and other mainstream and social media, and even through academic publications, can contribute to widespread non-adherence to public health directives, thereby perpetuating the spread, impact, and burden of a pandemic.

Infodemics can have increasingly devastating effects in economically disadvantaged countries, due to a wide range of inequalities (11), which can make people local populations more susceptible to fake news and misinformation (12, 13). This vulnerability is compounded by further inequalities in terms of healthcare infrastructure, access to healthcare facilities, and health professionals (14). Evidence suggests that, in Iran, the infodemic spread, largely through social media, contributed to several adverse outcomes in the general population (32). The speed, scale, and potential lethal consequences of infodemics are why they are considered parallel pandemics, which require a dedicated, strategic, expertise-informed response to allay.

The second inspiration for the study was an appreciation for the vital mitigating role that effective risk communication information management (RCIM) can play in pandemic and infodemic response. Understanding the sources of mis- and disinformation and rapid, effective government and public health response, in collaboration with multi-sector and community leaders, to evolving risks, along with targeted strategies, can mitigate potential negative ramifications.

The purpose of this research was to support increased national and local RCIM capacity in Iran and beyond by creating a unique conceptual model of evidence-, expert-, and experience-informed strategies for RCIM during epidemics and health emergencies. To create the model, we applied a systems thinking lens, since infodemics and their effects reside within multi-sectoral complex systems involving interactions and actors from all aspects of society. This perspective considers how to most effectively engage with potential audiences and diverse stakeholders, including the community, scientists and experts, government and public health officials, health workforce, pharmaceutical industries (private sector), and others, through physical and virtual communication channels (Figure 2). This comprehensive approach can enhance the potential for sectoral and provincial health authorities to improve RCIM activities and relevant health outcomes during epidemics and health emergencies. Given this perspective, following leading international pandemic research (27), we gathered two stages of input and validation from diverse groups of those with expertise and experience in public health and various related sectors and disciplines.

Figure 2 Components of the COVID-19 risk communication and infodemic management in Iran, source: own production

The model presented here is organised according to four of the pillars of the WHO model of the health systems, along with media and community (28), and it is reinforced by the full consensus of an expert panel in terms of its quality, completeness, and validity. While the model was developed for the Iranian context, the intention was for it to have potential application in other contexts to decrease the spread and burden of future health emergencies and to minimise global health disparities.

For peer review only - http://bmjopen.bmj.com/site/about/guidelines.xhtml

What follows are some insights on, and priority points for, effective RCIM that emerged from the qualitative interviews and expert panel discussions.

Our findings support the vital importance and potential impact of establishing a robust, integrated, evidence-informed national RCIM strategy, with regional applications, to strengthen existing RCIM capacities to explore, track, monitor, respond, and adapt to the needs of each community. Our results also show that effective RCIM requires several essential components: an official RCIM strategy supported by dedicated personnel, infrastructure, financing, and resources, trustworthy leadership and governance, the expertise and capacity to inform policies and to gather, analyse, and communicate the best available information in real-time, effective messaging through mainstream and social media with local support, RCIM training for specialists among the healthcare workforce, and community engagement to maximise local outcomes.

Official RCIM strategy with dedicated personnel, infrastructure, financing, and resources
Effective RCIM requires having an official strategy, based on a credible conceptual framework, which drove this study, and consolidated lessons learned locally and elsewhere. Aspects of the strategy need to evolve and adapt based on changing circumstances and it is essential that consideration is given to roles and customised approaches at the national, regional, and community levels. This should involve an official core national rapid response team with clear roles, protocols, resources, and accountabilities, along with regional chapters.

Second, the strategy needs to be supported by the infrastructure, financing, and resources to operate effectively. Respondents in our study suggested that in Iran, however, funding to enhance RCIM system capacity in terms of infrastructure and personnel is poor and they indicated that the lack of direct funding hindered the risk communication support during the pandemic. Although there are media and public communication experts, the number of those available with expertise and training in responding to major health risks is critically limited. Underfunding RCIM appears to be a common challenge in many countries. Evidence from south-east Asia (37), for example, revealed that during the COVID-19 pandemic, few countries allocated resources to emergency risk communication. However, some specific areas have budgets, such as information education communication materials. Also, resource mobilisation and the use of non-governmental resources were reported as strategies to address this critical challenge within the country's national and provincial settings. Priority areas and optimal mobilisation and use of resources is an important consideration for further exploration.

Leadership and governanc
The COVID-19 pandemic has highlighted the global importance of trustworthy and effective leaders who keep people at the forefront of their decisions, which they make transparently based on the best available evidence from a systems thinking perspective, and hold themselves accountable for outcomes (27). Leadership and governance are also at the heart of the WHO model of health systems (28). Similarly, every respondent in our study reinforced the fundamental importance to effective RCIM of leadership and governance.

For peer review only - http://bmjopen.bmj.com/site/about/guidelines.xhtml

Leadership-wise, effective RCIM response involves ensuring that the official RCIM strategy, personnel, infrastructure, and resources identified in the previous point are in place. But these are insufficient on their own.

Effective RCIM leadership and governance depends on government officials and public health and other leaders earning people's trust through their integrity and public- versus self-interest. If either of these are considered compromised, RCIM efforts are vastly undermined, as was seen during the pandemic in Iran. Leaders also earn trust by instilling confidence that, in a timely manner, they have the expertise and capacity to access and interpret the most credible information, operationalise an evidence-informed strategy and adapt it when necessary, and make and communicate transparent decisions, along with their rationale. Credible information should be actively gathered from many sources, including international, national, and local experts, leaders in all related sectors, and community leaders and representatives. Effective leaders understand that tailored, two-way communication according to an accurate understanding of each stakeholder's and community's preferences is crucial. This communication involves asking important questions, active listening, sharing information, providing clear recommendations, tools, and customised messaging, and engaging local support to lead RCIM. Finally, respondents indicated that leaders need to hold themselves publicly accountable for outcomes.

Leaders' ability to deliver on their responsibilities requires the aforementioned strategy, personnel, infrastructure, and resources, as well as developing a network of diverse international, national, and local experts in various relevant disciplines, leaders from all sectors, RCIM specialists within the health workforce, and community leaders.

Information

Effective RCIM relies on three approaches to information. The first is the expertise and capacity to, in a timely manner, proficiently screen, monitor, and verify the validity, relevance, and potential impact of available information from official and unofficial sources. The second is the ability to actively gather information from those with relevant expertise related to pandemic response and to RCIM strategies. The third is to communicate the most credible information to inform policymakers, government officials, public health, community leaders, and health and healthcare practitioners to equip them with the knowledge to create, implement, and adapt appropriate and effective strategies.

Media and communications

Combatting infodemics hinges on credible and strategic messaging through official sources, including government and public health websites, as well as through mainstream and social media, in collaboration with local representatives. The collaborative contribution of the government, public health, leaders in various sectors, experts, and community leaders in circulating health information is a key strategy to counter mis- or disinformation during health emergencies. Understanding the needs, perceptions, priorities, and concerns of key stakeholders across public and private settings and identifying different opportunities and strategies for their involvement are critical steps to developing and implementing risk communication policies and strategies.

Developing or sustaining reputed and well-trusted communication channels is critically required to maximise the effectiveness and impact of communication strategies. How the community perceives various epidemics and health emergencies, what they perceive to be their role, how they are influenced, and how their views tally with the biomedical approach, are not entirely investigated in the country.

For peer review only - http://bmjopen.bmj.com/site/about/guidelines.xhtml

According to our findings, a lack of public trust in mass media and government channels directed Iranian citizens to the wide use of online social networks. Due to the dramatic reduction in social capita, most Iranians distrust governmental information sources, and this fact challenged the community's compliance with preventive behaviours (COVID-19 vaccination) during the COVID-19 pandemic. Lack of trust in the government as a source of information was reported globally in the existing literature. According to recent evidence, only 40% of the European citizens from the Economic Co-operation and Development countries participated in a survey and trusted their governments as sources of information about the Corona Virus (38). False claims about the activities, statistics, or policies of public and government authorities were reported as a major source of disinformation during the COVID-19 pandemic, suggesting that "governments have not always succeeded in providing clear, useful, and trusted information to address pressing public questions" (39). Meanwhile, disinformation and claims may also be falsely attributed to official and governmental sources, amplifying this problem. In this regard, delivering truthful, evidence-informed, and compelling information to various audiences through their preferred channels and understanding behavioural and psychological biases is recommended. This is especially important for young audiences, who tend to access news and information predominantly via social media platforms(40). It is, therefore, a critical issue for health risk communication and infodemic management to ensure key factual messages reach all audiences. It is also important to effectively leverage the channel through which various audiences are relayed since different groups are likelier to trust media outlets that align with their views.

RCIM training for health workforce

While some capacity-building workshops for health professionals were held during the COVID-19 pandemic by the Ministry of Health and medical universities, they were largely been ad hoc, of short duration (less than a week), and of variable quality. Those trained have often been public health professionals who then move on to other areas of public health. A planned and institutionalised approach to capacity-building is required to have an adequate pool of trained experts for epidemics and health emergencies. Therefore, financial resources and building risk communication expertise are critical priorities for the country. Obtaining both these resources will require the endorsement of senior policymakers. Advocacy to policy-makers and key decision-makers on the role and impact of RCIM is very important.

Training

RCIM is a broad and multi-disciplinary field involving health communication, health education, public affairs, behaviour change communication, and social mobilisation. It is therefore required to build the capacity of key contributors to verify, filter, and curate health information and use diverse communication channels to target public audiences (41). Community-based organisations, patient advocacy groups, professional associations, and non-governmental organisations with reputable brands, organisational resources, and a network of relationships can be leveraged to improve health risk communications. Existing evidence demonstrates that by partnering with local public health experts and policymakers to create information hubs and community outreach programs (42), these groups can significantly improve their ability to serve the information needs and concerns of diverse communities while also advocating for policy solutions. Existing evidence demonstrates that involving community members as planners, and attendees in pre-crisis planning activities, leads to increased preparedness and response activities. Therefore training in roles and responsibilities, relationship building, and team-building are required

For peer review only - http://bmjopen.bmj.com/site/about/guidelines.xhtml

strategies to facilitate and strengthen the contribution of community-based organisations, expert associations, and other relevant partners during epidemics and health emergencies (43).

Community engagement

Effective RCIM depends on engaging with communities to share information and to understand their unique concerns, experiences, wisdom, available resources, and preferred forms of communication, as well as to earn the support of community leaders as key intermediaries in response. These measures can maximise community collaboration and receptivity to ensuing recommendations. Given the social, contextual, economic, and geographical diversity that exists within countries, customised, community-based approaches essential for RCIM and health emergency response. Ethnographic and anthropological/social research on epidemics and health emergencies in the country could also help to improve understanding of the acceptability of response to emergencies and public health interventions. According to our interviews and expert panel discussion, the community was considered a missing piece in RCIM strategies in Iran. Information needs and concerns (e.g., disabilities, gender, age, literacy, cultural/ethnic backgrounds, access to technology) of the general Iranian population remained unexplored. In addition, the participatory engagement of citizens in a collective response to the COVID-19 infodemic was not only insufficient, but rather, at times, it was discouraged.

During the COVID-19 pandemic, national health authorities and governments in most countries predominantly demonstrated top-down communication strategies (44). Effective RCIM requires a whole-of-society effort to sustain a healthy information ecosystem. Understanding the needs and concerns of vulnerable groups who might experience barriers to accessing accurate health information, care, and support, or be at higher risk of exposure and secondary impacts, such as children and adults with disabilities, is critically important(44). Effective risk communication can save lives during epidemics and health emergencies; however, existing evidence revealed that inadequate risk communication resulted in high exposure and loss of lives, as seen in Iran and Italy in the first wave (44, 45). Training and advising the general population on how to consume and share health information responsibly may be an effective strategy to improve the engagement and participation of public communities in risk communication and infodemic management. Investing in the community's media literacy, health literacy, and critical thinking skills before the crisis can prepare society to mitigate the physical and emotional consequences of false news and disinformation and increase resilience (46). As disinformation and infodemic during epidemics and health emergencies undermine trust, amplify fears, and consequently affect countries' responses to the global pandemic, tailored strategies to build and maintain trust among the public community are of utmost importance. Therefore, to be effective and foster public trust in government, any activities conducted in health risk communication and infodemic management must be guided by the principles of transparency, integrity, accountability, and community participation.

Limitations

We address some limitations of the study. First, given that our study and the novel conceptual framework presented here are the first to address comprehensively the RCIM needs of, and strategies for, the Iranian health system context, further research and validation of its completeness and reliability, particularly after attempts to implement it, would be useful. Similarly, investigating causality and replicating the study with identical results can be challenging with qualitative studies of complex phenomena. However, involving diverse sets of respondents with experience and expertise in leading RCIM in two phases of research before reaching total consensus heightens the potential for the framework to be considered

For peer review only - http://bmjopen.bmj.com/site/about/guidelines.xhtml

credible and effective in being applied in the Iranian context. Further research could focus on applying best practices in RCIM, ecosystem mapping and analysis, and strengthening data collection and analysis for monitoring, evaluation, and learning. Investigating specific methods for evaluating RCIM activities are also important and critically recommended. Second, by focusing on the Iranian context, the transformability of the framework to other contexts remains yet untested. However, the high-level results echo leading international research on effective pandemic response and even if regional customisation would be beneficial, the current framework could potentially represent a well-informed basis for discussion, for further research, and for the creation of local versions.

Conclusion

This study was inspired by an appreciation for the extent to which the COVID-19 infodemic is reported to have impacted the spread and burden of the disease globally, and of the role that an effective risk communication and information management (RCIM) strategy can play in mitigating the impact of infodemics. The purpose of this research was to support increased RCIM capacity in Iran and beyond through the creation of a unique conceptual model of evidence-, expert-, and experience-informed strategies for RCIM during epidemics and health emergencies. Our findings suggest that ineffective RCIM impeded the emergency response in Iran's COVID-19 management, which is partly attributable to Iran's government and national public health authorities failing to infuse an evidence-informed and strategic RCIM into policy- and decision-making. Consequently, access to high-quality and real-time information was extensively restricted and not publicly available, and the provincial public health settings failed to establish effective community relationships with experts, researchers, professional councils, and NGOs to facilitate knowledge translation and utilisation. Further, the extensive use of social media platforms and mass media worsened the circulation of rumours, fake news, and disinformation and led to public distrust. The lessons learned from the outbreak management and response in Iran suggest that RCIM should be an essential component of health emergency readiness and response activities. This begins with trustworthy leaders at all levels who have integrity and make credible, transparent decisions, and hold themselves accountable for outcomes. A national RCIM program should be established to support the required infrastructures, personnel, and processes to address communication challenges during epidemics and health emergencies. This should be based on a conceptual model of RCIM to illustrate a collaborative and interdependent context of risk communication activities, implying that any improvements in these areas requires an integrated and holistic approach. The government, private sector and pharmaceutical industries, experts, and the public should be involved in time, contributing diverse views and fulfilling respective responsibilities. The conceptual model presented here has the potential to be either implemented or serve as the foundation for the creation of a similar model in other contexts. Sharing experiences, challenges, and leading practices among jurisdictions can further improve the reliability and credibility of guidance and strategies.

Ethics approval and consent to participate

This study was approved by the Research Ethics Committee of the Kerman University of Medical Sciences (**IR.KMU.REC.1400.379).** The Declaration of Helsinki was followed and informed consent was obtained from participants before starting the data collection stage.

For peer review only - http://bmjopen.bmj.com/site/about/guidelines.xhtml

Availability of data and materials

The datasets used and/or analysed during the current study are available from the corresponding author upon reasonable request.

Competing interests

All authors declared that they had no competing interests.

Funding
This study was financially supported by World Health Organization-Regional Office for the Eastern Mediterranean (WHO-EMR) Call for Proposals for Special Grant for COVID-19 Research, 2022 (**WHO Reference: 2022/1291032-0**)

Authors' contributions

AB contributed to the project concept and manuscript design, qualitative data collection and interpretation, critical review of the manuscript writing, and discussion of the manuscript. AS worked on data analysis, data interpretation, and writing of the manuscript. MSB worked on data analysis, data interpretation, and writing of the manuscript. HM worked on literature search, data interpretation, and writing of the manuscript. MS worked on data analysis, data interpretation, and writing of the manuscript. JMG worked on data analysis, data interpretation, and writing and revising of the manuscript. HS worked on the project concept and manuscript design, supervising, critically review of the manuscript writing, and discussion the manuscript. All authors read and approved the final manuscript.

Patient or public involvement

No patients or members of the public were involved in the present study.

Acknowledgments
The authors wish to thank the provincial and national public health professionals who contributed to this study, the WHO for their funding support, and the *BMJ Open* editor and peer reviewers for their valuable insights.

References

1. Muñoz-Sastre D, Rodrigo-Martín L, Rodrigo-Martín I. The Role of Twitter in the WHO's Fight against the Infodemic. International Journal of Environmental Research and Public Health. 2021;18(22):11990.
2. Mheidly N, Fares J. Leveraging media and health communication strategies to overcome the COVID-19 infodemic. Journal of public health policy. 2020;41(4):410-20.
3. The Lancet Infectious D. The COVID-19 infodemic. The Lancet Infectious diseases. 2020;20(8):875-.
4. Health Topics: Infodemic [Internet]. Available from: https://www.who.int/health-topics/infodemic#tab=tab_1.
5. Yang K-C, Pierri F, Hui P-M, Axelrod D, Torres-Lugo C, Bryden J, et al. The covid-19 infodemic: Twitter versus facebook. Big Data & Society. 2021;8(1):20539517211013861.
6. Patel MP, Kute VB, Agarwal SK, Nephrology C-WGoISo. "Infodemic" COVID 19: More Pandemic than the Virus. Indian journal of nephrology. 2020;30(3):188-91.

For peer review only - http://bmjopen.bmj.com/site/about/guidelines.xhtml

7. Maharlouei N, Hosseinpour P, Erfani A, Shahriarirad R, Raeisi Shahrakie H, Rezaianzadeh A, et al. Factors associated with reluctancy to acquire COVID-19 vaccination: A cross-sectional study in Shiraz, Iran, 2022. Plos one. 2022;17(12):e0278967.
8. Charkazi A, Allah Kalteh E, Yatimparvar G, Rahimzadeh H, Koochaki G, Shahini N, et al. Prevalence of COVID-19 Vaccination Hesitancy and its Associated Factors based on the Health Belief Model among Iranian People in 2021. Health Education Health Promotion. 2022;10(4):679-85.
9. Mirzaie A, Halaji M, Dehkordi FS, Ranjbar R, Noorbazargan H. A narrative literature review on traditional medicine options for treatment of corona virus disease 2019 (COVID-19). Complementary therapies in clinical practice. 2020;40:101214.
10. Khaniki H, Rasi Tehrani H. Theorizing for Covid 19 infodemic in Iran social media. Quarterly of Social Studies Research in Iran. 2022:933-50.
11. Vinck P, Pham PN, Bindu KK, Bedford J, Nilles EJ. Institutional trust and misinformation in the response to the 2018–19 Ebola outbreak in North Kivu, DR Congo: a population-based survey. The Lancet Infectious Diseases. 2019;19(5):529-36.
12. Sørensen K, Pelikan JM, Röthlin F, Ganahl K, Slonska Z, Doyle G, et al. Health literacy in Europe: comparative results of the European health literacy survey (HLS-EU). European journal of public health. 2015;25(6):1053-8.
13. Lorini C, Ierardi F, Bachini L, Donzellini M, Gemmi F, Bonaccorsi G. The antecedents and consequences of health literacy in an ecological perspective: results from an experimental analysis. International journal of environmental research and public health. 2018;15(4):798.
14. Carr-Hill R, Currie E. What explains the distribution of doctors and nurses in different countries, and does it matter for health outcomes? Journal of Advanced Nursing. 2013;69(11):2525-37.
15. Zar HJ, Dawa J, Fischer GB, Castro-Rodriguez JA. Challenges of COVID-19 in children in low-and middle-income countries. Paediatric respiratory reviews. 2020;35:70-4.
16. Geldsetzer P. Knowledge and perceptions of COVID-19 among the general public in the United States and the United Kingdom: a cross-sectional online survey. Annals of internal medicine. 2020;173(2):157-60.
17. Islam MS, Sarkar T, Khan SH, Kamal A-HM, Hasan SM, Kabir A, et al. COVID-19–related infodemic and its impact on public health: A global social media analysis. The American journal of tropical medicine and hygiene. 2020;103(4):1621.
18. Kim HK, Ahn J, Atkinson L, Kahlor LA. Effects of COVID-19 misinformation on information seeking, avoidance, and processing: A multicountry comparative study. Science Communication. 2020;42(5):586-615.
19. Lockyer B, Islam S, Rahman A, Dickerson J, Pickett K, Sheldon T, et al. Understanding COVID-19 misinformation and vaccine hesitancy in context: Findings from a qualitative study involving citizens in Bradford, UK. Health Expectations. 2021;24(4):1158-67.
20. Mirhashemi SMH. Infodemic in the countries around the world after corona virus outbreak; Necessity of Media literacy and information literacy improvement for citizens. Applied Politics. 2022;3(1):239-50.
21. World Health Organization. Infodemic management: an overview of infodemic management during COVID-19, January 2020–May 2021. 2021.
22. Tangcharoensathien V, Calleja N, Nguyen T, Purnat T, D'Agostino M, Garcia-Saiso S, et al. Framework for managing the COVID-19 infodemic: methods and results of an online, crowdsourced WHO technical consultation. Journal of medical Internet research. 2020;22(6):e19659.
23. Lohiniva A-L, Nurzhynska A, Hudi A-h, Anim B. Infodemic Management Using Digital Information and Knowledge Cocreation to Address COVID-19 Vaccine Hesitancy: Case Study From Ghana. JMIR infodemiology. 2022;2(2):e37134.
24. Naeem SB, Bhatti R. The Covid-19 'infodemic': a new front for information professionals. Health Information & Libraries Journal. 2020;37(3):233-9.
25. Eysenbach G. How to fight an infodemic: the four pillars of infodemic management. Journal of medical Internet research. 2020;22(6):e21820.

For peer review only - http://bmjopen.bmj.com/site/about/guidelines.xhtml

26. Radu R. <? covid19?> Fighting the 'Infodemic': Legal Responses to COVID-19 Disinformation. Social Media+ Society. 2020;6(3):2056305120948190.
27. Hua J, Shaw R. Corona virus (Covid-19)"infodemic" and emerging issues through a data lens: The case of china. International journal of environmental research and public health. 2020;17(7):2309.
28. Organization WH. Everybody's business: strengthening health systems to improve health outcomes: WHO's framework for action. Geneva: WHO; 2007. 2018.
29. Bradley EH. Intersectoral collaboration: what works and what doesn't. International Journal of Health Policy Management. 2023.
30. Hiller M, Bracht H, Schroeder S. One year with the COVID-19 pandemic– Lessons learnt? Intersectoral collaboration measures established during the crisis could benefit capacity and patient flow management in daily clinical practice. Journal of Health Organization Management. 2022;36(2):141-8.
31. White D. Application of systems thinking to risk management:: a review of the literature. Management Decision. 1995.
32. Olson CM. Consensus statements: applying structure. JAMA. 1995;273(1):72-3.
33. Geerts JM, Kinnair D, Taheri P, Abraham A, Ahn J, Atun R, et al. Guidance for health care leaders during the recovery stage of the COVID-19 pandemic: a consensus statement. JAMA network open. 2021;4(7):e2120295-e.
34. Dossett LA, Kaji AH, Cochran A. SRQR and COREQ reporting guidelines for qualitative studies. JAMA surgery. 2021;156(9):875-6.
35. Braun V, Clarke V. Using thematic analysis in psychology. Qualitative research in psychology. 2006;3(2):77-101.
36. Press TA. WHO chief warns world leaders against "politicizing" pandemic. CBC News. 2020.
37. Ofrin R, Buddha N, Htike M, Bhola A, Bezbaruah S. Strengthening risk communication systems for public health emergencies in the WHO South-East Asia Region. WHO South-East Asia Journal of Public Health. 2020;9(1):15-20.
38. Eldman. Eldman Trust Baromter 2020 2020 [Available from: https://www.edelman.com/sites/g/files/aatuss191/files/2020-03/2020%20Edelman%20Trust%20Barometer%20Coronavirus%20Special%20Report_0.pdf.
39. Brennen JS, Simon FM, Howard PN, Nielsen RK. Types, sources, and claims of COVID-19 misinformation: University of Oxford; 2020.
40. Neavel C, Watkins SC, Chavez M. Youth, social media, and telehealth: How COVID-19 changed our interactions. Pediatric Annals. 2022;51(4):e161-e6.
41. Goulbourne T, Yanovitzky I. The communication infrastructure as a social determinant of health: implications for health Policymaking and practice. The Milbank Quarterly. 2021;99(1):24.
42. Hering JG. Do we need "more research" or better implementation through knowledge brokering? Sustainability Science. 2016;11(2):363-9.
43. World Health Organization. Communicating risk in public health emergencies: a WHO guideline for emergency risk communication (ERC) policy and practice: World Health Organization; 2017.
44. Khan S, Mishra J, Ahmed N, Onyige CD, Lin KE, Siew R, et al. Risk communication and community engagement during COVID-19. International Journal of Disaster Risk Reduction. 2022;74:102903.
45. Chakrabarti A. Confusion in Italy, clarity in Singapore — how govts are communicating during coronavirus 2020 [cited 2022 26/11]. Available from: https://theprint.in/world/confusion-in-italy-clarity-in-singapore-how-govts-are-communicating-during-coronavirus/378932/.
46. Xie L, Pinto J, Zhong B. Building community resilience on social media to help recover from the COVID-19 pandemic. Computers in Human Behavior. 2022;134:107294.

For peer review only

For peer review only - http://bmjopen.bmj.com/site/about/guidelines.xhtml

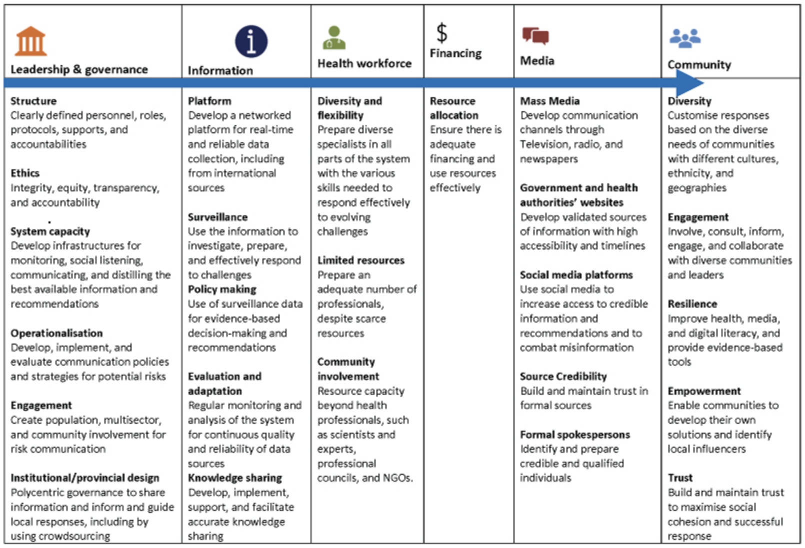

Figure 1

668x471mm (38 x 38 DPI)

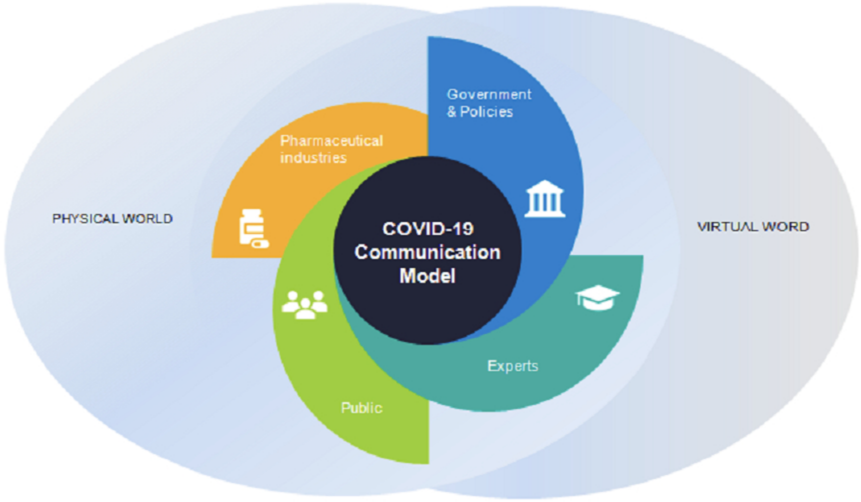

Figure 2

235x136mm (108 x 108 DPI)

Interview guide

Part I: Demographic information

Age ……….

Gender Female / Male

Educational level ……….

Academic discipline ………

What organizational position were you involved in at the time of the Covid 19 epidemic? ………..

Part II: Perceptions, experiences, future directions

The main purpose of the questions in this section is to identify effective strategies and successful experiences in the field of infodemic management related to Covid 19 in Kerman province. Please answer the following questions based on your experiences or field observations.

1- The spread of misleading, inaccurate, and fake information about COVID-19 disease and vaccination has been one of the consequences of the COVID-19 epidemic, which affects the behavior of society and trust in the health system. What experience did you have in managing misinformation? What did you do in a situation in the province where accurate information was not yet available? Can you explain your own experiences in this field?
2- 2. What challenges and obstacles did you face in combating inaccurate information and infodemic management?
3- What did you do in response to the obstacles and challenges?
4- How did you find out about the effectiveness of your interventions and actions?
5- 5- If the pandemic situation is repeated, what is your approach to managing infodemic?

For peer review only

Informed Consent

Hi,

My name is Azam Bazrafshan. My colleague and I are from the Kerman University of Medical Sciences. We interview executives, technical experts, decision-makers, and leaders of public health initiatives who had participated in the provincial, or national COVID-19 prevention and control programs to use the results to improve health interventions during epidemics, pandemics, and global health crises. We are intended to investigate processes, infrastructure, strategies, successful experiences and challenges in the field of infodemic management related to Covid 19 in Kerman province. You are being invited to take part in this research because we feel that your experience as a public health leader can contribute much to our understanding and knowledge of processes and infrastructure of infodemic management during health epidemics.

Your participation in this research is entirely voluntary. It is your choice whether to participate or not. If you choose not to participate all the services you receive at this Centre will continue and nothing will change.

In this interview, I will not ask your name, nor will I need your address. All your answers will be completely confidential. We only use the total responses for statistical survey. During this interview, private questions may also be asked and I have to emphasize that although your honest cooperation is valuable, you can answer any question you think appropriate. The estimated time of interview is about 30 minutes and the interview is recorded by tape recorder.

You do not have to take part in this research if you do not wish to do so, and choosing to participate will not affect your job or job-related evaluations in any way. You may stop participating in the interview at any time that you wish without your job being affected. I will give you an opportunity at the end of the interview to review your remarks, and you can ask to modify or remove portions of those, if you do not agree with my notes or if I did not understand you correctly.

I have read the foregoing information, or it has been read to me. I have had the opportunity to ask questions about it and any questions I have been asked have been answered to my satisfaction. I consent voluntarily to be a participant in this study

Signature of Participant ___________________

Date ___________________________Day/month/year

Signature

For peer review only

Appendix 3. Themes and subthemes related to the challenges and successful activities of infodemic management across provincial/national public health settings in Iran

Theme	Sub-theme	Sample Quotes
**Leadership & governance**		
Ethics	Transparency	When I report falsely on the radio, people would understand. If I have this transparency at the national and university level, people will gain their trust, but this strategy does not exist, it certainly does not exist, neither at the national level, nor at the university or provincial level. (Man, 47 years old)
		There is a level of transparency that the government should have, the officials should have, I would say that it is extremely small considering the structure of our country. (Man, 42 years old)
	Accountability	No one was responsible, a disaster happened, I learned by myself, it was unknown to us, we could have managed this if the ministry had interfered less, the ministry acted badly and this bad behavior spread everywhere.(Man, 55 years old)
	Conflicts of interests	Conflict of interests is one of the most important challenges in the Ministry of Health, which hinders transparency. Profit seeking of companies that produce personal protective equipment, diagnostic and therapeutic equipment, and vaccines, is One of the most important examples of conflicts of interests during the pandemic management. (Man, 70 years old)
Capacity	Infrastructures	We need a quick reaction team that is in contact with academic centers, regularly monitors and examines community's needs and concerns. (Man, 49 years old)<br><br>... and can quickly identify rumours and false information and design an answer to them. (Man, 42 years old)<br><br>A multi-disciplinary risk communication team should be formed to be responsible for informing and making decisions. (Man, 58 years old)
	Rules & regulations	During the pandemic, there were people who published false and contradictory information, and there was no authority to deal with this issue and deal with them judicially, while in many countries, when the issue of people's health is discussed, false information is not allowed to be published. (Man, 70 years old)
	Policies and strategies	There is no specific strategy and program to deal with infodemic and manage risk communication in the country. (Man, 70 years old)
	Role definition	The duties and responsibilities of people in crisis situations should be clear so that everyone does not act and speak as they wish... The goals of the programs should be clear and the responsibilities of each member of the committees should be clearly stated. (Man, 53 years old)
Operational isation	Timeliness	In order to prepare in crisis conditions, it is necessary to make necessary plans before every crisis. (Man, 45 years old)
	Coordination	One of our most important problems was the lack of coordination between the government and the officials in the matter of information. There were several voices and no coordination between different sources in the health department. (Man, 58 years old)

For peer review only

Institutional/provincial design	Knowledge exchange	In the discussion of pandemic management, the provinces were left to their own devices, and no province was aware of the activities of other provinces in the management of the infodemic. (Man, 55 years old)
**Information**		
**Theme**	**Sub-theme**	**Sample quotes**
Surveillance	Timeliness	No real-time data were available about the mortality of COVID-19 cases. Even now, if we refer to the statistical systems, we will not get a correct and reliable information and coordination so that we can inform the community. (Man, 48 years old)<br><br>Unfortunately, many of the events that happened in the country have not been documented and, for example, we do not know how many patients were infected with Corona, how many died. Even the medical and nursing systems used to come and give general information. It showed that either they don't have this information or they don't want to publish it.(Man, 49 years old)
	Data quality	No consensus data were available as mortality data reported by the hospitals, forensic systems, and cemeteries, were very contradictory. (Man, 48 years old)
	Surveillance systems	Surveillance data were extremely out of date, with low quality and consistency. (Man, 54 years old)<br><br>Our surveillance data were not accurate and real-time. (Man, 48 years old)<br><br>Therefore, it seems that we need a system that collects information from the environmental levels in a database in the form of software that has the power of analysis to give us alarms in different places. Its infrastructure in the country is planned as a syndromic care system, but I don't know if it has actually been implemented. (Man, 49 years old)
Knowledge translation	Evidence-informed policy making	No evidence about the effectiveness of interventions were synthesised and published for decision making. (Man, 54 years old)<br><br>The next problem was that the correct information did not reach those who should manage infodemic, for example, the number of patients at any moment, the number of deaths, what was the cause of death. (Man, 52 years old)
	Knowledge translation Capacity	Most of the statistics and information will be based on taste and subjective and this will cause individual perceptions and people will allow themselves to give any statistics. (Man, 48 years old)
Health workforce		
**Theme**	**Sub-theme**	**Sample Quote**
Capacity building	Diversity and flexibility	That's why we have to find an entry in the educational fields and teach this issue seriously in the form of workshops for groups close to graduation or students in the form of refresher courses regularly and continuously. Let's define a retraining unit for it and implement it operationally, not just theoretically. (Man, 48 years old)
Challenges	Limited resources	One of our most important challenges in risk communication and infodemic management is the lack of trained and expert people in this field. (Man, 70 years old)

For peer review only

Engagement	Community involvement	The non-participation of experts from different scientific fields in the pandemic management and informing the people was a big challenge, which caused people who had good experience or knowledge to be ignored, and therefore, unqualified people were in charge of informing the people. (Man, 42 years old)
**Financial resources**		
**Theme**	**Sub-theme**	**Sample Quote**
Government resources	Planning	Particular financial sources should be allocated to the risk communication activities. (Man, 66 years old)
**Media**		
**Theme**	**Sub-theme**	**Sample Quote**
Communication channels	Mass media	National TV and Radio channels are still the biggest and most influential communication channels in Iran. (Man, 46 years old)
	Government & health authorities' websites	We used both video media such as radio and television, as well as written media such as magazines, newspapers, and government websites, which were very active during the Corona era, to communicate the data related to the incidence of the disease in the province and recommend preventive measures. (Man, 49 years old)
	Social media platforms	We have established a social media platform (Instagram) named Dr+ to communicate with people and held online discussion panels with contribution of clinical physicians to address the community's needs and concerns. (Man, 53 years old)
Trust	Source credibility	Communication channels should be used that are highly credible and people trust to them. Some brand communication channels should be developed to maximize the impact and penetration of information among people. (Man, 47 years old)
spokespersons	Competence	For a person to be a spokesperson and to give information, to know how to give information, not to be too hopeful, not to speak too hopelessly, this is real information when we say not only to report numbers... For example, when the pandemic came, someone said that there is nothing, someone said Wow, we are unfortunate, which one of these people should accept when they look at it? (Man, 58 years old)
**Community**		
**Theme**	**Sub-theme**	**Example Strategies**
Diversity	Social context	The penetration rate of scientific issues in our society is low, which is related to various issues, so if we ever want to increase this penetration rate, we have to approach from different social and cultural aspects. (Man, 54 years old)
Engagement & empowerment	interventions	We have developed a community engagement facility to listen the community's needs and expectations and answer to their concerns and questions interactively. (Man, 55 years old)<br><br>Safiran-e-Salamat was a group of trained volunteers who communicate health information with their families and their neighbourhood. (Man, 55 years old)

For peer review only

Trust	Trust to government and health officials	Public opinion has no trust in the government, especially in matters that are officially announced. (Man, 47 years old)<br><br>Anyone, any scientist, any distinguished person, any accepted person comes and says something, the first time people do not accept it, especially if it is actually what the government says or emphasizes, people will definitely look for the opposite and say that there is something fishy about it. (Man, 53 years old)

For peer review only

Standards for reporting qualitative research (SRQR) checklist

No	Topic	Item	Page number
		Title and abstract	
S1	Title	Concise description of the nature and topic of the study Identifying the study as qualitative or indicating the approach (e.g., ethnography, grounded theory) or data collection methods (e.g., interview, focus group) is recommended	1
S2	Abstract	Summary of key elements of the study using the abstract format of the intended publication; typically includes background, purpose, methods, results, and conclusions	1
		Introduction	
S3	Problem formulation	Description and significance of the problem/phenomenon studied; review of relevant theory and empirical work; problem statement	2
S4	Purpose or research question	Purpose of the study and specific objectives or questions	3
		Methods	
S5	Qualitative approach and research paradigm	Qualitative approach (e.g., ethnography, grounded theory, case study, phenomenology, narrative research) and guiding theory if appropriate; identifying the research paradigm (e.g., postpositivist, constructivist/ interpretivist) is also recommended; rationale	4
S6	Researcher characteristics and reflexivity	Researchers' characteristics that may influence the research, including personal attributes, qualifications/experience, relationship with participants, assumptions, and/or presuppositions; potential or actual interaction between researchers' characteristics and the research questions, approach, methods, results, and/or transferability	-
S7	Context	Setting/site and salient contextual factors; rationale	4
S8	Sampling strategy	How and why research participants, documents, or events were selected; criteria for deciding when no further sampling was necessary (e.g., sampling saturation); rationale	4

For peer review only

S9	Ethical issues pertaining to human subjects	Documentation of approval by an appropriate ethics review board and participant consent, or explanation for lack thereof; other confidentiality and data security issues	4
S10	Data collection methods	Types of data collected; details of data collection procedures including (as appropriate) start and stop dates of data collection and analysis, iterative process, triangulation of sources/methods, and modification of procedures in response to evolving study findings; rationale	4-5
S11	Data collection instruments and technologies	Description of instruments (e.g., interview guides, questionnaires) and devices (e.g., audio recorders) used for data collection; if/how the instrument(s) changed over the course of the study	4-5
S12	Units of study	Number and relevant characteristics of participants, documents, or events included in the study; level of participation (could be reported in results)	NA
S13	Data processing	Methods for processing data prior to and during analysis, including transcription, data entry, data management and security, verification of data integrity, data coding, and anonymization/deidentification of excerpts	4-5
S14	Data analysis	Process by which inferences, themes, etc., were identified and developed, including the researchers involved in data analysis; usually references a specific paradigm or approach; rationale	4-5
S15	Techniques to enhance trustworthiness	Techniques to enhance trustworthiness and credibility of data analysis (e.g., member checking, audit trail, triangulation); rationale	5
Results/findings			
S16	Synthesis and interpretation	Main findings (e.g., interpretations, inferences, and themes); might include development of a theory or model, or integration with prior research or theory	5
S17	Links to empirical data	Evidence (e.g., quotes, field notes, text excerpts, photographs) to substantiate analytic findings	6-appendix 3
Discussion			
S18	Integration with prior work, implications, transferability, and contribution(s) to the field	Short summary of main findings; explanation of how findings and conclusions connect to, support, elaborate on, or challenge conclusions of earlier scholarship; discussion of scope of application/ generalizability; identification of unique contribution(s) to scholarship in a discipline or field	11
S19	Limitations	Trustworthiness and limitations of findings	13
Other			

For peer review only

S20	Conflicts of interest	Potential sources of influence or perceived influence on study conduct and conclusions; how these were managed	14
S21	Funding	Sources of funding and other support; role of funders in data collection, interpretation, and reporting	15

For peer review only
